# *Ocepeia* (Middle Paleocene of Morocco): The Oldest Skull of an Afrotherian Mammal

**DOI:** 10.1371/journal.pone.0089739

**Published:** 2014-02-26

**Authors:** Emmanuel Gheerbrant, Mbarek Amaghzaz, Baadi Bouya, Florent Goussard, Charlène Letenneur

**Affiliations:** 1 Centre de Recherches sur la Paléobiodiversité et les Paléoenvironnements, CNRS-MNHN-UPMC, Muséum National d'Histoire Naturelle, Dpnt Histoire de la Terre, Paris, France; 2 Office Chérifien des Phosphates (OCP SA), Centre Minier de Khouribga, Khouribga, Morocco; Team ‘Evo-Devo of Vertebrate Dentition’, France

## Abstract

While key early(iest) fossils were recently discovered for several crown afrotherian mammal orders, basal afrotherians, e.g., early Cenozoic species that comprise sister taxa to Paenungulata, Afroinsectiphilia or Afrotheria, are nearly unknown, especially in Africa. Possible stem condylarth-like relatives of the Paenungulata (hyraxes, sea-cows, elephants) include only *Abdounodus hamdii* and *Ocepeia daouiensis* from the Selandian of Ouled Abdoun Basin, Morocco, both previously only documented by lower teeth. Here, we describe new fossils of *Ocepeia*, including *O.grandis* n. sp., and a sub-complete skull of *O. daouiensis*, the first known before the Eocene for African placentals. *O.daouiensis* skull displays a remarkable mosaic of autapomophic, ungulate-like and generalized eutherian-like characters. Autapomorphies include striking anthropoid-like characters of the rostrum and dentition. Besides having a basically eutherian-like skull construction, *Ocepeia daouiensis* is characterized by ungulate-like, and especially paenungulate-like characters of skull and dentition (e.g., selenodonty). However, some plesiomorphies such as absence of hypocone exclude *Ocepeia* from crown Paenungulata. Such a combination of plesiomorphic and derived characters best fits with a stem position of *Ocepeia* relative to Paenungulata. In our cladistic analyses *Ocepeia* is included in Afrotheria, but its shared derived characters with paenungulates are not optimized as exclusive synapomorphies. Rather, within Afrotheria *Ocepeia* is reconstructed as more closely related to insectivore-like afroinsectiphilians (i.e., aardvarks, sengis, tenrecs, and golden moles) than to paenungulates. This results from conflict with undetected convergences of Paenungulata and Perissodactyla in our cladistic analysis, such as the shared bilophodonty. The selenodont pattern best supports the stem paenungulate position of *Ocepeia*; that, however, needs further support. The remarkable character mosaic of *Ocepeia* makes it the first known “transitional fossil” between insectivore-like and ungulate-like afrotherians. In addition, the autapomorphic family Ocepeiidae supports the old – earliest Tertiary or Cretaceous – endemic evolution of placentals in Africa, in contrast to hypotheses rooting afrotherians in Paleogene Laurasian “condylarths”.

## Introduction

The "condylarths" – or archaic ungulates – are the main and most spectacular radiation of the placental mammals at the beginning of the Tertiary, after the demise of the non-avian dinosaurs. This non-monophyletic group includes various ungulate-grade lineages, some of which became extinct more or less quickly by the Eocene, and some other of which succeeded and gave rise to the extant flourishing ungulate orders such as Perissodactyla and Artiodactyla.

The fossil record of the “condylarths” is heterogeneous, especially geographically. They are well known in the Early Tertiary of the Laurasian continents such as North America, Asia, and Europe, where they diversified in various lineages, including primitive and modern taxa. North American and European “condylarths” were known since the turn of the late 19th century, and have been the subject of several classical studies [1]–[7].

In South Tethyan areas, South America has yielded a diversified Paleogene ungulate fauna that illustrates a remarkable endemic radiation (“Panameriungulata”, “Meridiungulata” [8], [9], [10]); one that did not survive past the Great American Biotic Interchange [12], [11]. India is much more poorly known. The fossil record of Indian ungulate-grade mammals pre-dating the Eocene is restricted to one possible late Cretaceous “condylarth” [13]. Relevant earliest Paleogene mammals from India come from the Early Eocene and include rare “condylarths” such as Quettacyonidae [14]–[17], and basal representatives of modern clades such as Anthracobunidae, Perissodactyla and Artiodactyla [17]–[24].

Together with India, the Arabo-African province is the most poorly known continental center of evolution for ungulate-grade and other placental mammals [25], [26]. This is related to the general problem of the poor Cretaceous and early Paleogene fossil record of Africa. Consequently, our knowledge of the origin and early evolution of the African ungulate-like afrotherians, the paenungulates (elephant, sea cow, hyrax), was based until recently almost entirely on molecular studies [27]–[31], and lacked detailed information about the possible phenotypes present in this group during the earliest Paleogene.

The earliest placental mammal faunas known in Africa have been found in the Paleocene and early Eocene of Morocco, in the Ouarzazate and Ouled Abdoun Basins. The Ouarzazate fauna [32], [33], [34] unfortunately yielded only micromammals that mostly include primitive, insectivoran-grade eutherians. The Ouled Abdoun phosphate sediments have yielded rare but well preserved fossils of what are the earliest known placentals from Africa. The first discoveries were made in the Ypresian of the northern quarries of Grand Daoui that yielded the earliest known fossils of paenungulates, and especially of proboscideans [35]–[38]. The “condylarths” were found later, at the turn of the 21th century, with the discovery of new Paleocene sites in the quarries of Sidi Chennane [39], in the Ouled Abdoun phosphate Basin. The first described remains were scarce lower jaw fragments from unknown local sites that were identified as *Ocepeia daouiensis* and *Abdounodus hamdii* [39]. More recently, an important new fossil material was discovered, especially in well recognized Paleocene strata and sites of the Sidi Chennane quarries; it includes more or less complete lower jaws [26], and some exceptional cranial remains preserving the upper dentition that are described and studied here.

Recent geochemical studies [40], [41] have confirmed the Selandian age (ca. 59–60 ma) of the Paleocene level from Sidi Chennane quarries, Ouled Abdoun basin, that yields mammals such as *Ocepeia daouiensis*.

## Material, Method of Study

### Abbreviations

#### Institutional acronyms and acronyms of paleontological collections

OCP DEK/GE: Collections of the Office Chérifien des Phosphates, Khouribga, Morocco.

CPSGM, Collections Paléontologiques du Service Géologique du Maroc; deposited in the OCP Collections, Khouribga, Morocco.

MNHN.F: Collections of the Muséum National d'Histoire Naturelle (F: Paleontology), Paris, France.

MHNT PAL: Muséum National d'Histoire Naturelle of Toulouse, France, collection of Paleontology.

PM: Phosphate of Morocco, localities of the Ouled Abdoun Basin, Morocco.

TZT, THR and NTG2: material from localities of respectively Talazit, Adrar Mgorn 1 and N'Tagourt 2, Ouarzazate Basin, Morocco; collection of the University Montpellier II, France.

BD, locality of M'Bodione Dadere, Senegal, collections of the University Montpellier II, France.

#### Other abbreviations

Primitive and derived states are abbreviated (p) and (d) in the text; they refer with respect to the generalized eutherian condition, except when mentioned (e.g., placental and paenungulate conditions). The primitive eutherian condition is represented by Cretaceous taxa such as *Eomaia*, *Acristatherium*, *Asioryctes*, *Maelestes*, *Zalambdalestes*, cimolestids, zhelestids, etc… (see Text S1, Part II, Taxa analyzed). These polarized states are established following our preliminary comparisons, as primary homologies for the matrix and before the cladistic analysis that results is the identification of secondary homologies (e.g., synapomorphies). The autapomorphic states are abbreviated (a).

### Field Work

Permission for field work in the Ouled Abdoun OCP quarries was provided by the Office Chérifien des Phosphates (OCP S.A., Morocco) and the Ministère de l'Energie, des Mines, de l'Eau et de l'Environnement (MEMEE, Morocco). Field work (prospects and excavations) was only geological and paleontological, and did not involved endangered or protected species.

### Measurements

Measurements are provided in millimeters (mm). The stapedial ratio was measured following Segall [42] and the cochlear curvature following West [43], and with help of CT scan 3D modelisation.

### CT Scan, 3D modelisation, softwares

MNHN.F PM 45 was subjected to X-ray Computed Tomographic (CT) imaging at the AST-RX platform of the MNHN, using a GE Sensing and Inspection Technologies phoenix|x-ray v|tome|x L240-180 CT scanner. We used the microfocus RX source 240 kV/320 W, detector 400×400 mm with a matrix of 2024 pixels (pixel size: 200×200 µm). Scan parameters: Voltage = 95 kV; Current = 515 µA; Exposure: 200 ms; Isotropic voxel size of 0.04900265 mm. Data were reconstructed using datos|x reconstruction software (Phoenix|x-ray, release 2.0) and then exported into a 16 bits TIFF image stack of 1717 virtual slices in transversal view.

We used MIMICS Innovation Suite software (Materialise, release 16) for the analysis, 3D modelisation and measurements on 3D model.

Corrections of distortions and reconstructions were also made with the help of the software VG studio Max (Volume Graphics, release 2.2) and Cinema 4D (Maxon, release 13).

### Nomenclatural Acts

The electronic edition of this article conforms to the requirements of the amended International Code of Zoological Nomenclature, and hence the new names contained herein are available under that Code from the electronic edition of this article. This published work and the nomenclatural acts it contains have been registered in ZooBank, the online registration system for the ICZN. The ZooBank LSIDs (Life Science Identifiers) can be resolved and the associated information viewed through any standard web browser by appending the LSID to the prefix “http://zoobank.org/”. The LSID for this publication is: urn:lsid:zoobank.org:pub:13D2D3D4-AC74-444E-A943-C069F10294ED. The electronic edition of this work was published in a journal with an ISSN, and has been archived and is available from the following digital repositories: PubMed Central, LOCKSS.

## Results

Systematic Paleontology

Cohort Placentalia Owen, 1837

Supercohort Afrotheria Stanhope, Waddell, Madsen, De Jong, Hedges, Cleven, Kao, Springer, 1998

Superorder ?Paenungulata Simpson, 1945

Order *incertae sedis* (prob. nov.)

*Family* ***Ocepeiidae*** *nov. Gheerbrant*

**Diagnosis**. That of the type and only known genus.

**Type genus**. *Ocepeia* Gheerbrant & Sudre, 2001.

Included **genera**. *Ocepeia*, only know genus.

Distribution. Same as the type genus.

**ZooBank life science identifer (LSID) for family**.urn:lsid:zoobank.org:act:5260AD60-6655-49C8-BD6C-D3C7D577A1EF.

Genus *Ocepeia* Gheerbrant & Sudre, 2001

**Diagnosis** (a: autapomorphies).

Skull (unknown in *O. grandis*): Short and broad rostrum with very robust construction; short frontal with reduced orbito-temporal process; meso- and postero-cranial region elongated, especially the parietal; parietals with two oblique bony ridges diverging anteriorly from the mid part of the sagittal crest; extensive pneumatization of the skull bones, especially of the supraoccipital; middle ear and inner ear (pars cochlearis and pars canalicularis) remarkably small; tegmen tympani large and inflated.

Dentary: Symphysis short and partially fused (a); condyle significantly higher than the tooth row; corpus high and transversely inflated.

Dentition: Dental formula: I3, C, P2, M3. Anterior dentition shortened (a), with P1–2 lost (a), no significant diastemata (a), and lower incisors compressed with root wide and short (a). I_3_ and probably I^3^ vestigial (a). Lower canine stout and anthropoid-like (lingual cingulum present, asymmetrical labio-lingual profile) (a). Postcanine dentition noticeably large relative to the palate extension (megadontia).

Molar pattern bunoselenodont and brachydont with well developed labial shearing crests (associated with semi-lunar shearing wear facets). Molars size increasing slightly from M1 to M3.

Crown of lower premolars and molars inflated labially (a).

P_ 3-4_ simple and trenchant.

Lower molars: paraconid bulbous and lingual in M_1-2_, but median transversely in M_3_; paraconid with small mesial crest; protoconid low, close in height to metaconid and paraconid; postmetacristid and metastylid present; mesoconid large; small entoconulid; hypoconid very large, low and broad (a); entoconid with a short but functional entolophid linking the lingual flank of the hypoconid; hypoconulid reduced and lingual in M_1–2_ and postcristid very long, in relation to the selenodont pattern; short premetacristid; postcristid bearing several cuspules; no labial cingula. M_2_ wider than M_1_ and M_3_; M_3_ at least as long as M_2_, with expanded hypoconulid lobe, and with oblique distal root.

Upper cheek teeth more or less homodont (a): upper premolars extended transversely with well-developed protocone.

Upper molars: parastyle and especially mesostyle large; W-like ectoloph linked to the strong mesostyle; small additional stylar cusps and crest present; hypocone absent; conules and accessory conules present; protocone low, mesio-distally expanded with widely divergent crests at apex; protocone lingual flank strongly canted labially; wide protofossa; very small lingual crest/ridge at the base of the paracone; lingual cingulum thin and continuous.

**Type species**. *Ocepeia daouiensis* Gheerbrant & Sudre, 2001.

**Included species**. *O daouiensis*; *O. grandis* n.sp.

**Locality and age**. Paleocene of the Ouled Abdoun Basin, Morocco.

***Ocepeia daouiensis*** Gheerbrant & Sudre, 2001

(Figs. 1-13, Figs. S1-2, Videos S1-2)

**Figure 1:**
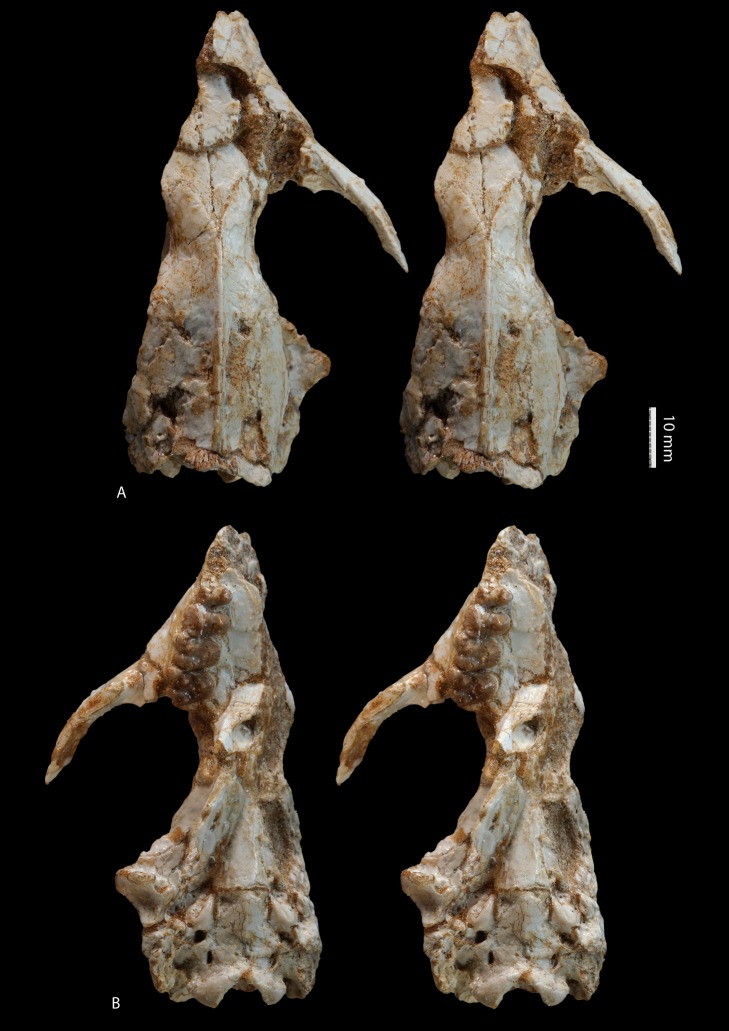
*Ocepeia daouiensis*, Selandian, Phosphate level IIa of Sidi Chennane, Ouled Abdoun Basin, Morocco. **A**. Stereophotographic dorsal view of the skull MNHN PM45. **B**. Stereophotographic ventral view of the skull MNHN PM45. Scale bar: 10 mm.

**Figure 2:**
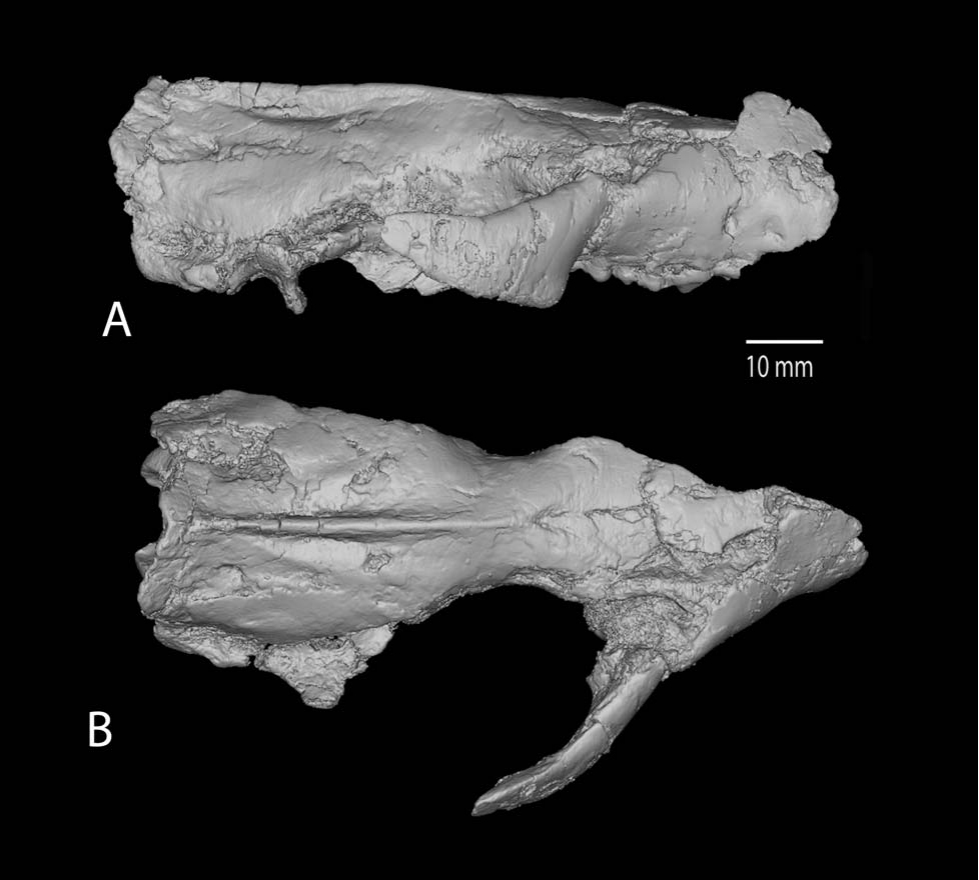
*Ocepeia daouiensis*, Selandian, Phosphate level IIa of Sidi Chennane, Ouled Abdoun Basin, Morocco. 3D CT scan model of the skull MNHN PM45 in lateral (A) and dorsal (B) views. Scale bar: 10 mm.

**Figure 3:**
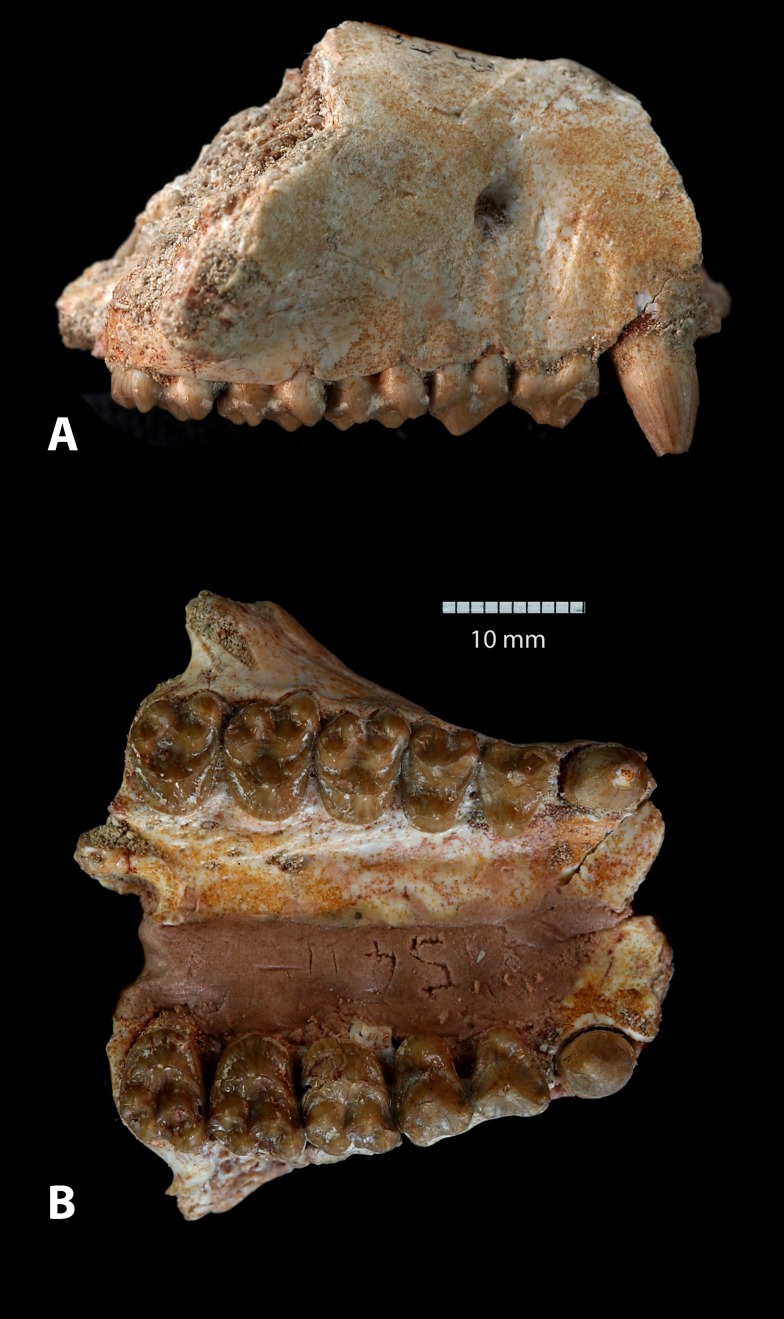
*Ocepeia daouiensis*, Selandian, Phosphate level IIa of Sidi Chennane, Ouled Abdoun Basin, Morocco. MNHN.F PM54-4 maxillary in labial (a) and occlusal view (b). Cheek teeth: right and left C^1^, P^3–4^, M^1–3^. Scale bar: 10 mm.

**Figure 4:**
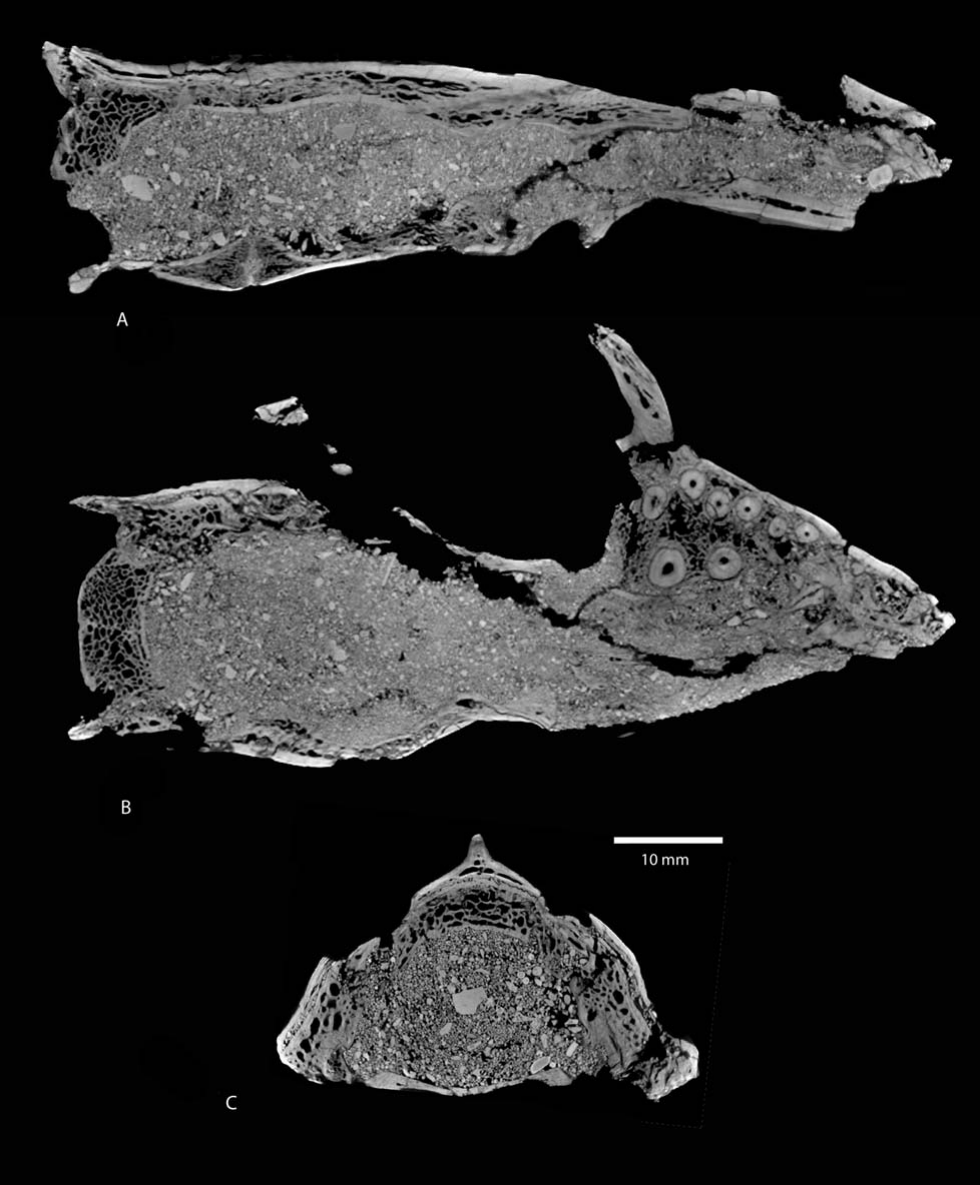
*Ocepeia daouiensis*, Selandian, Phosphate level IIa of Sidi Chennane, Ouled Abdoun Basin, Morocco. CT scan sections of the skull MNHN.F PM45; A. Sagittal section YZ 299; C. Horizontal section B-XZ; C. Transverse section XY 704 B-XZ. Scale bar: 10 mm.

**Figure 5:**
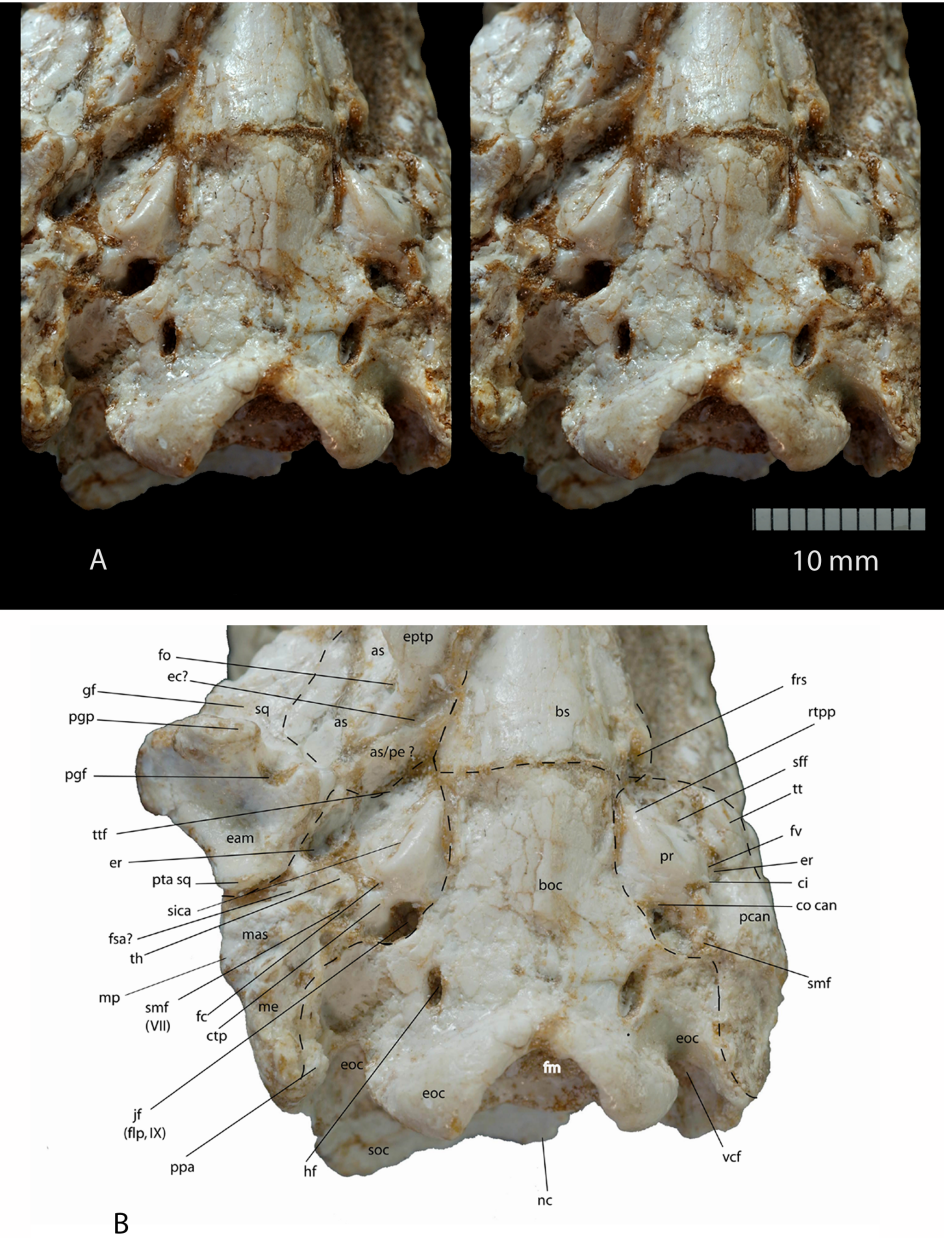
*Ocepeia daouiensis*, Selandian, Phosphate level IIa of Sidi Chennane, Ouled Abdoun Basin, Morocco. Skull MNHN.F PM45, detail of the basicranium in ventral view. A. Stereophotographic ventral view; B. Annotated ventral view. Scale bar: 10 mm. Abbreviations: co can, cochleae canaliculus  =  aquaeductus cochleae; ci, crista interfenestralis; ctp, caudal tympanic process; eam, external auditory meatus; ec, eustachian canal eoc, exoccipital eptp, ectopterygoid process er, epitympanic recess; fc, fenestra cochleae (f. rotunda); fo, foramen ovale; fr, foramen rotundum frs, foramen for superior ramus of stapedial artery; fm, foramen magnum; fsa, foramen for ramus superior artery fv, fenestra vestibuli (f. ovalis); gf, glenoid fossa; hf, hypoglossal foramen; jf, jugular foramen ( =  flp: foramen lacerus posterius, nerve IX); lctpp, lateral section of caudal tympanic process of petrosal; oc, occipital condyle; mas, pars mastoidea me, mastoid exposure; mp, mastoid process pcan, pars canalicularis pgf, postglenoid foramen; pgp, postglenoid process; pr, promontorium; pta sq: posttympanic apophysis of the squamosal; ppa: paroccipital apophysis: rtpp, rostral tympanic process of petrosal; sff, secondary facial foramen; sica, sulcus for internal carotid artery; smf, stylomastoid foramen (facial nerve); sq/pe, suture between squamosal and petrosal; th, tympanohyal; tt, tegmen tympani; ttf, tensor tympani fossa; vcf, ventral condylar fossa.

**Figure 6:**
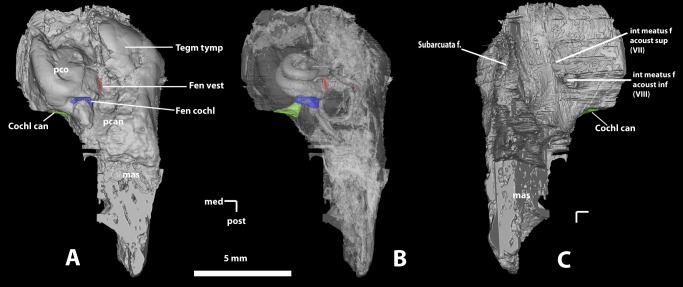
*Ocepeia daouiensis*, Selandian, Phosphate level IIa of Sidi Chennane, Ouled Abdoun Basin, Morocco. MNHN.F PM45, 3 D CT scan modelling of the left periotic in ventral view showing middle ear anatomy (A), transparency showing inner ear anatomy (B) and dorsal (cerebellar) view (C). Abbreviations: cochl can: cochlear canal; fen cochl: fenestra cochleae (f. rotunda); fen vest: fenestra vestibuli (f. ovalis); med: medial; tegm tymp: tegmen tympani; post: posterior; subarcuata foss: subarcuata fossa. Scale bar: 5 mm.

**Figure 7:**
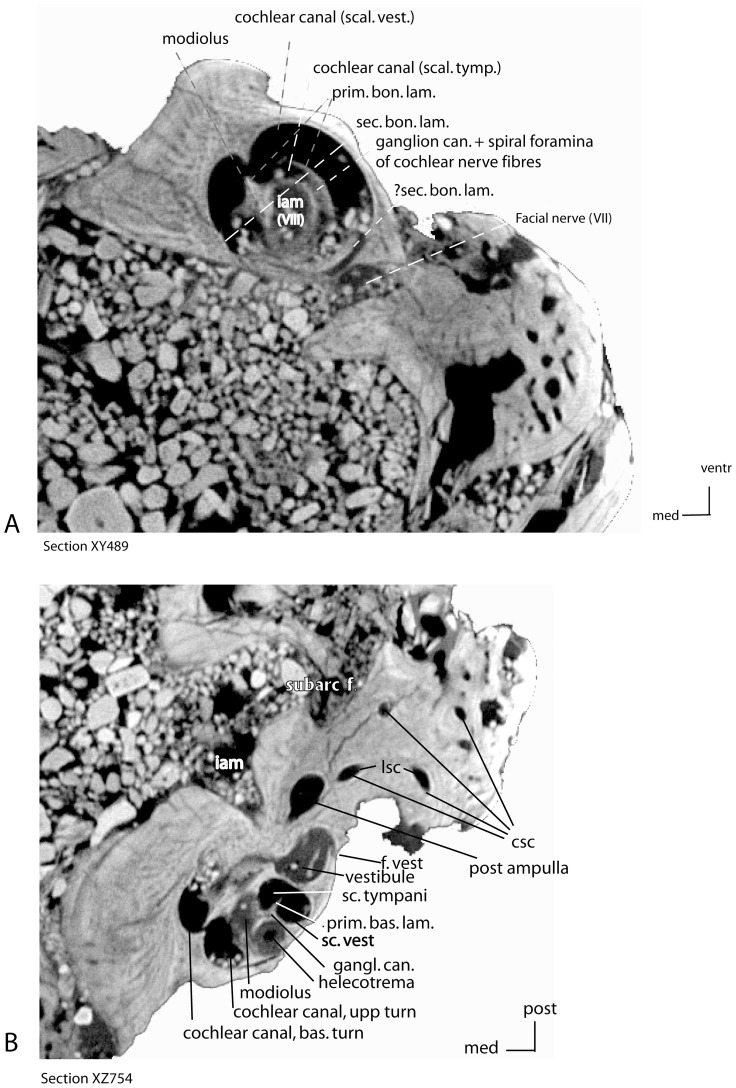
*Ocepeia daouiensis*, Selandian, Phosphate level IIa of Sidi Chennane, Ouled Abdoun Basin, Morocco. Skull MNHN.F PM45, CT scan sections of the left periotic; A. vertical section XY 489 (1); B. horizontal section_ XZ_754.

**Figure 8:**
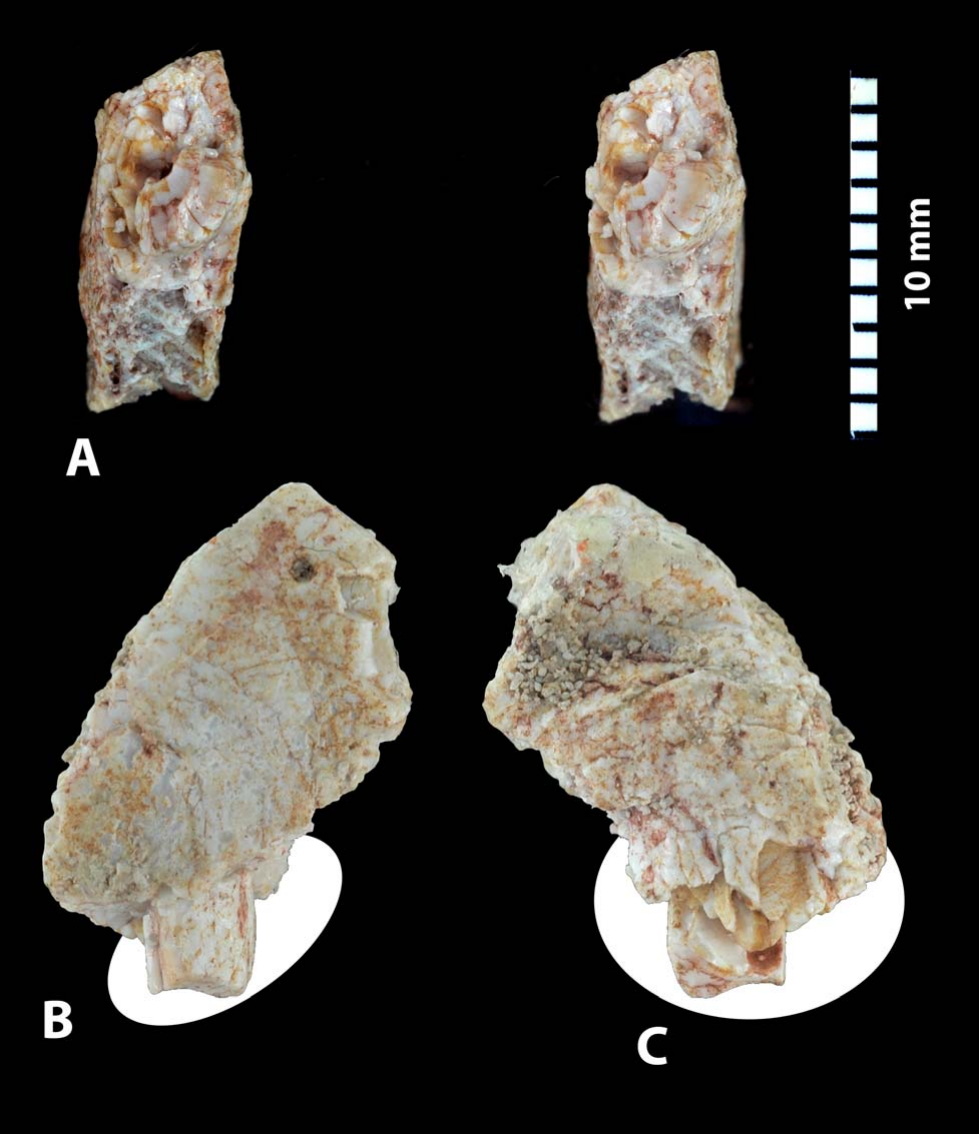
*Ocepeia daouiensis*, Selandian, Phosphate level IIa of Sidi Chennane, Ouled Abdoun Basin, Morocco. Specimen MNHN.F PM54 (male individual), left premaxillary fragment preserving part of I^2^; in occlusal stereophotographic view (A), labial (B) and lingual (C) views. Scale bar: 10 mm.

**Figure 9:**
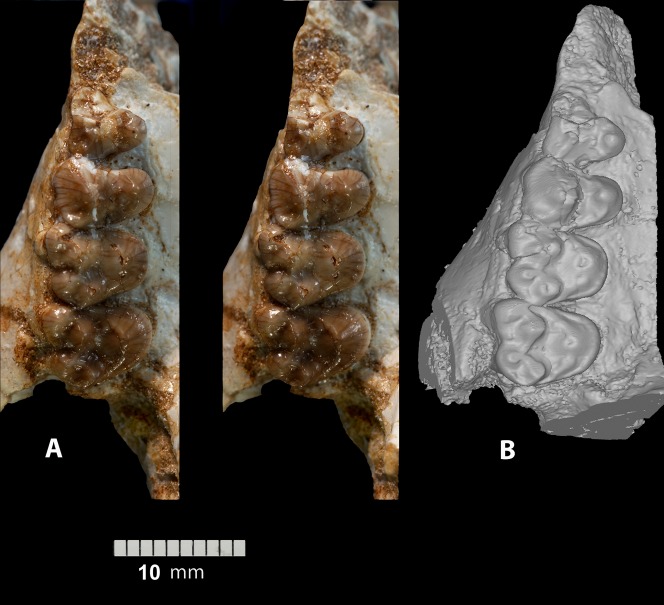
*Ocepeia daouiensis*, Selandian, Phosphate level IIa of Sidi Chennane, Ouled Abdoun Basin, Morocco. Specimen MNHN.F PM45-1 (female individual), right maxillary with P^3–4^, M^1–2^, in occlusal stereophotographic view (A), and 3D modelling from CT scan images (B). Scale bar: 10 mm.

**Figure 10:**
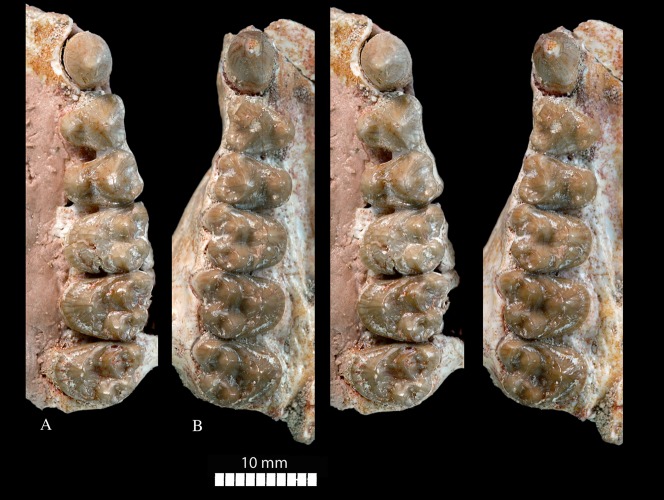
*Ocepeia daouiensis*, Selandian, Phosphate level IIa of Sidi Chennane, Ouled Abdoun Basin, Morocco. *S*pecimen MNHN.F PM54 (male individual), upper right (A) and left (B) tooth rows with C^1^, P^3–4^, M^1–3^, in occlusal setereophotographic view.

**Figure 11:**
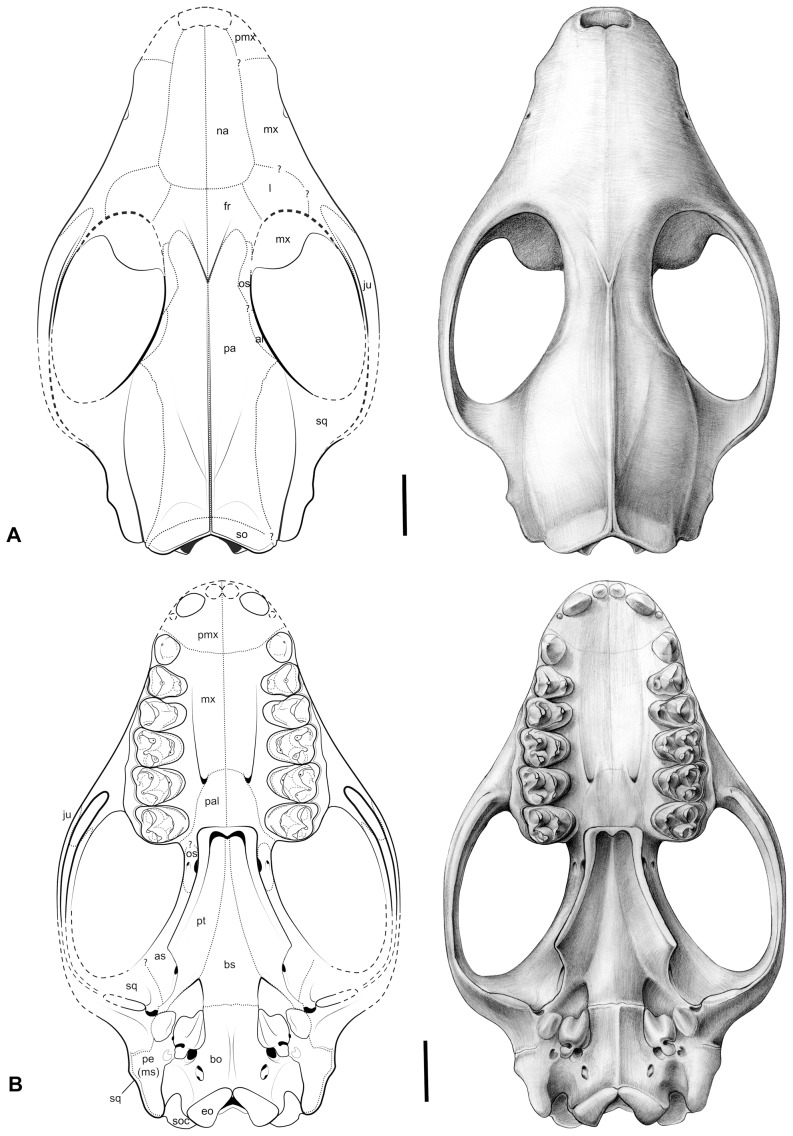
*Ocepeia daouiensis*, Selandian, Phosphate level IIa of Sidi Chennane, Ouled Abdoun Basin, Morocco *O. daouiensis*. Reconstruction of the skull. A. dorsal view; B. Ventral view. This reconstruction of the skull is based on the female specimen MNHN.F PM45. The specimen MNHN.F PM54, a male individual, is distinct from MNHN.F PM45 with a more robust skull morphology and presence of a stronger sagittal crest. Drawings: C. Letenneur (MNHN). Scale bar:10 mm.

**Figure 12:**
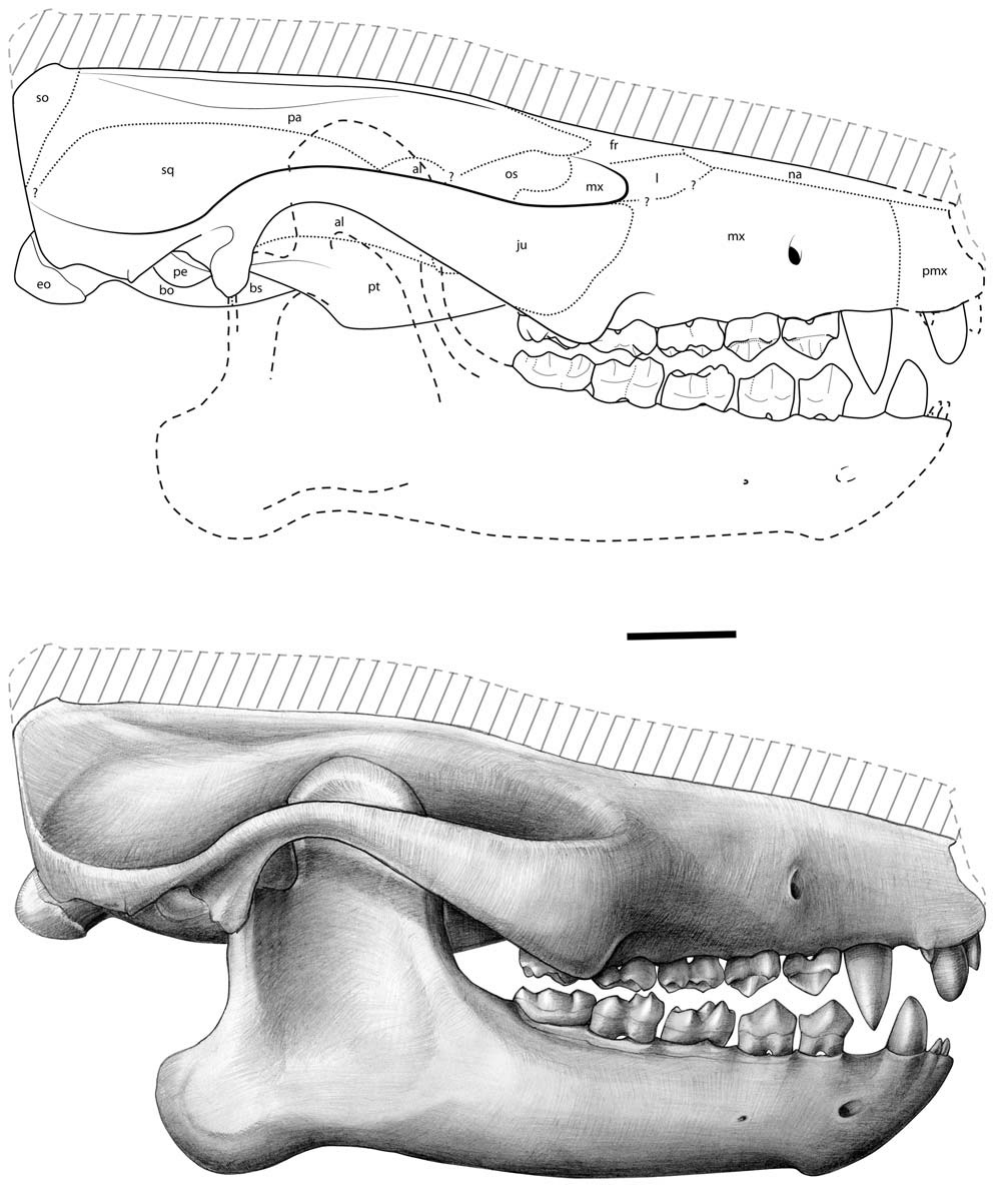
*Ocepeia daouiensis*, Selandian, Phosphate level IIa of Sidi Chennane, Ouled Abdoun Basin, Morocco *O. daouiensis*. Reconstruction of the skull. Lateral view. This reconstruction of the skull is based on the female specimen MNHN.F PM45. The specimen MNHN.F PM54, a male individual, is distinct from MNHN.F PM45 with a more robust skull morphology and presence of a stronger sagittal crest. The dashed line (hatched area) at skull roof corresponds to our estimated correction of the plastic dorso-ventral crushing of MNHN.F PM45 that could not be quantified precisely on this specimen, even with the CT scans (see comments in text, §Reconstruction); the correction is based on comparison with MNHN.F PM54. The lower jaw was reconstructed based on specimen MNHN.F PM41 [26]. Drawings: C. Letenneur (MNHN).

**Figure 13:**
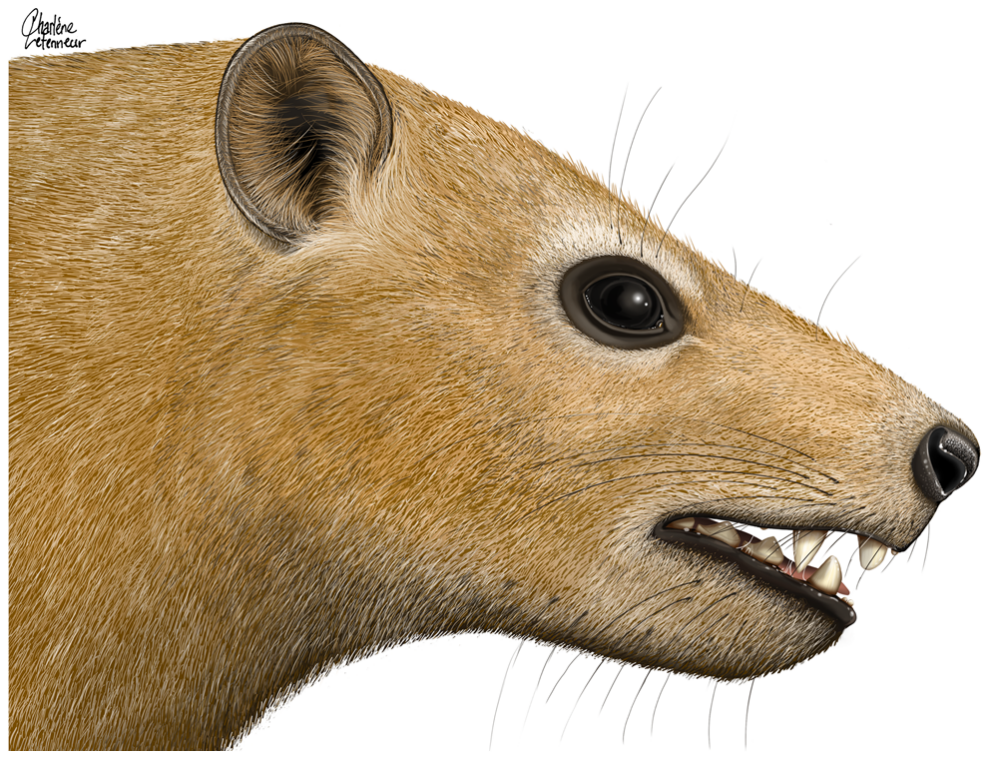
*Ocepeia daouiensis*, Selandian, Phosphate level IIa of Sidi Chennane, Ouled Abdoun Basin, Morocco. Reconstruction of the head. This reconstruction is based on the bony skull reconstruction illustrated Figure 11, including our correction of the plastic dorso-ventral crushing of specimen MNHN.F PM45 (dashed line at skull roof in Figure 11). Drawing: C. Letenneur (MNHN).

**Age and occurrence**. Paleocene of the Ouled Abdoun Basin (quarries of Grand Daoui, Meraa El Arech, Sidi Chennane), Morocco. The new material of this species confirms its Paleocene age. It comes from the same bone bed level located at the base of phosphorite Bed IIa that is dated Selandian [40], [41] and that yields *Eritherium azzouzorum*, *Abdounodus hamdii*, and *Lahimia selloumi*.

***Holotype*** *. CPSGM-MA1, fragment of right dentary with P_4_, M_1_.*

**New referred material**. Most specimens from lower dentition of *O. daouiensis* were reported and described by Gheerbrant et al. [26], [39]. In this paper we refer and describe new specimens representing mostly the skull and its upper dentition that were previously unknown.

MNHN.F PM45, a partial skull broken on the left side (left maxillary and zygomatic arch absent)and bearing the right P^3–4^, M^1–2^); a small individual, possibly a female; Ouled Abdoun basin, quarries of Sidi Chennane, Selandian.

MNHN.F PM54, skull rostrum preserving right and left maxillary with C^1^, P^3–4^, M^1–3^, the frontal and anteriormost part of the parietal, a fragment of left premaxillary (with a large incisor); probably a large male individual; Ouled Abdoun basin, unknown exact locality, Selandian.

MNHN.F PM58, lower canine; Ouled Abdoun basin, unknown exact locality.

MHNT PAL 2006.0.17 (PM71), fragment of right dentary with M_1_ (reconstructed), P_4_; Ouled Abdoun basin, unknown exact locality; collection of the Museum of Toulouse.

MHNT PAL 2006.0.16 (PM72), fragment of left dentary with C_1_ (strongly worn), P_4_, M_1–2_; Ouled Abdoun, unknown exact locality; collection of the Museum of Toulouse.

**Diagnosis**. See Gheerbrant [26] and generic diagnosis; *Ocepeia daouiensis* differs mainly from *O. grandis* n. sp., the only other known species of the genus (see below), by its smaller size.

### Description: Cranium

#### Material

The new material reported here allows the first description of the skull and upper dental morphology of Ocepeia daouiensis. Two partial skulls of Ocepeia daouiensis, MNHN.F PM45 and MNHN.F PM54, are known. They show important differences in size and general robustness related to intraspecific individual variations.

MNHN.F PM45, a relatively complete cranium, is the best preserved specimen. This is the only and best known mammal skull from the Paleocene of Arabo-Africa. It preserves most part of the cranium, except for a small part of left side (left maxillary, palatine, jugal), the premaxillae, the distal part of the right zygomatic arch (jugal) and the right maxillary tuberosity bearing M_3_. MNHN.F PM45 belongs to a small individual with low sagittal crest. The preserved teeth (right P^3–4^, M^1–2^) are unworn and it is possible that M^3^ was still in crypt or just erupting in this specimen, indicating a young individual. However, the sutures are strongly fused and in many cases hardly distinct; together with the low sagittal crest, it might suggest a young adult female.

MNHN.F PM 54 corresponds to most of the rostrum of a cranium preserving the permanent cheek teeth (left and right C^1^, P^2–4^, M^1–3^), the anteriormost part of the parietal, the nasal, the frontal, a fragment of left premaxillary (with a large incisor), most of the right maxillary and the palatine, and part of the left maxillary. It belongs to a larger individual than MNHN.F PM45. It is also characterized by a more robust general construction with much stronger sagittal and temporal crests, and a large canine, all features probably related to sexual dimorphism. These characters suggest that MNHN.F PM54 belongs to a large male adult, in contrast to the individual represented by MNHN.F PM45. The lower jaw PM41 fits better in occlusion with MNHN.F PM 54 than with MNHN.F PM45, which might indicate that it also belongs to a male (young individual).

#### General morphology

Our reconstruction of the cranium of *Ocepeia daouiensis* is based on the specimen MNHN.F PM45 (Figs. 1–2, Fig. S1), with help of 3D digital modeling of tomographic image data. It should be noted that this specimen is most likely female individual with a more gracile morphology than MNHN.F PM54 (Fig. 3). The size of the skull is close to that of *Meniscotherium chamense* (estimated length 90 mm; width  = 65 mm).

The overall cranium shape is noticeably robust, with a broad outline in dorsal view (W/L = 70%). The most remarkable feature is that the rostrum is short and wide, whereas the cranial region (especially mesocranial region) is elongated. This is illustrated by a noticeably high ratio (82%) of the width of the snout at the canine level *versus* the preorbital length. This primate-like robust short and broad snout is autapomorphic in *Ocepeia*. The short rostrum might be related to the occurrence of enlarged upper incisors, as illustrated by specimen MNHN.F PM 54 for I^2^. The face (rostrum) is about 1/3 the total length, and the frontal and nasal are short. A short frontal is known is some Paleocene taxa such as *Meniscotherium* (e.g., Gazin [44], pl. 2) and *Pleuraspidotherium*, but in *Ocepeia* the orbital process of the frontal is unusually reduced. For instance, the orbitosphenoid has an original long suture with the elongated parietal and only a small antero-dorsal suture with the frontal. The elongation of the braincase and the mesocranial region is illustrated by the long basisphenoids and pterygoids, by the choanae located very anterior relative to the skull length, by the very long parietal and sagittal crest, and by the anterior position of the pars cochlearis of the petrosal. The postorbital constriction of the skull is strong as illustrated especially in PM 54; the skull and braincase are narrowest near the mid skull length.

Another remarkable feature of the cranium of *Ocepeia daouiensis*, that is evidenced by the CT scan sections, is the extensive pneumatization of the bones such as the supraoccipital, the parietal, the basisphenoid, the basioccipital and the periotic. This is most striking for the supraoccipital which is considerably thickened and expanded below the parietal, and which shows extensive diploë (Fig. 4). The pars mastoidea of the petrosal, the basisphenoid and the basioccipital are also noticeably pneumatized. This is a probable autapomorphy of *Ocepeia*.

After correction of the post-mortem distortion (based on 3D digital model and drawings; see §4), the zygomatic arches appear not strong flared laterally (see reconstruction and Fig. S2), in contrast to tethytheres, *Phosphatherium*, and also *Namatherium*. There is no postorbital process, so that the orbit is poorly individualized and widely confluent with the temporal fossa. The tooth row does not extend behind the skull mid-length (it extends about 45% of skull length). The skull is low and the dorsal profile is long and straight, as in pantolestids and in contrast to the generalized eutherian construction. The sagittal crest is moderate (MNHN.F PM45) to strong (MNHN.F PM 54), but it does not form a high blade-like structure; the nuchal (lambdoid) crests are strong and salient dorsally and laterally.

#### Dorsal view

The most striking feature is the short rostrum relative to the long cranial region. This is illustrated in dorsal view (Fig. 1) by a very short frontal and a contrastingly very long parietal that extends close to the orbit level and that bears a sagittal crest nearly as long as half the skull length. The anterior extension of the parietal in the skull roof resembles that of *Pleuraspidotherium* [7] and the primate *Adapis*; *Pleuraspidotherium* differs however by having much more developed orbito-temporal process of the frontal and by having the orbits that are more anterior with respect to the parietal; *Adapis* has also more anterior orbits, but it is closer to *Ocepeia* in the somewhat reduced orbito-temporal process of the frontal. The CT scan sections show that in fact the parietal extends anteriorly considerably above the frontal, on an estimated length of 10 mm.

The squamosal (*pars squamosa*) is also elongated. The premaxillary was probably short (see reconstruction). The nasal cavity seen in MNHN.F PM54 is high and wide, as in primitive proboscideans; this is probably a paenungulate trait. The nasals are broadened posteriorly. They do not penetrate deep in the frontals. The naso-frontal suture is transverse; it is located at the level between M^1^ and M^2^, as in the generalized eutherian condition. The maxillae are well-developed, with a high *processus frontalis* as in primitive proboscideans; the maxillo-nasal suture is long. The maxillary tuberosity was small and located close to the anterior rim of the orbit. The frontal is flat; it extends anteriorly from the temporal line, up to about 1 cm in front the maxillary tuberosity (M^3^ location).

The zygomatic process of the maxilla is well-developed laterally and it extends posteriorly on the medial part of the jugal up to the level of the beginning of the sagittal crest. The temporal fossa and zygomatic arches are longer than wide. The lacrimal is broken but its development can be inferred from surrounding preserved bones in MNHN.F PM45. The lacrimal forms the upper rim of the orbit with a large facial process (*pars facialis*), in the generalized eutherian condition. A slight bone inflation might indicate the presence of a lacrimal tubercle close to the jugal. The lacrimal has sutures with frontal and nasal dorsally. It is not clear if the lacrimal has a suture with the jugal or with the maxillary along the orbit rim. However, the jugal is narrowed in its anterior extremity (MNHN.F PM 45) as a short and flat bony blade above the zygomatic process of the maxillary. This suggests that the contact between the lacrimal and jugal was either slight or interrupted by a short intermediate extension of the maxillary onto the orbital rim; in other words, the jugal was slightly withdrawn posteriorly. The condition of *Ocepeia* is not far from that of basal paenungulates such as hyracoids where the lacrimal it is separated from the jugal by a narrow orbital process of the maxillary. The lacrimal foramen is present (MNHN.F PM 54) and located in the orbit above the maxillary foramen of the infraorbital canal. The infraorbital canal extends from the P^3^ to M^2^ levels, above the tooth row.

The postorbital constriction is strong and maximal at the transverse level where the temporal crests meet and unite with the sagittal crest. The parietals are remarkably long, even longer than in *Pleuraspidotherium* and *Hyopsodus*. The fronto-parietal suture follows the temporal crest. The postorbital process of the frontal is strikingly absent, as in *Hyopsodus* and primitive eutherians. The temporal fossa is very large. The zygomatic arches are moderately expanded laterally. There are two discrete but distinct ridges on the posterior part of the parietals that diverges anteriorly on the skull roof from the sagittal crest near the junction with the nuchal crest; this original feature might be autapomorphic. The braincase is well-developed (e.g., wide transversely).

#### Lateral view

The skull roof profile is very straight. The nasal and the muzzle are high. The maxilla extends high above the tooth row. The infraorbital foramen is much smaller than in *Phosphatherium* and in *Eritherium*. It is located not very high above P^3^, in the primitive eutherian condition. The orbit is noticeably posterior, with its anterior rim located at the level of M^2–3^, as in *Hyopsodus* and in contrast to primitive proboscideans (*Eritherium*: P^4^) and even hyracoids (*Dimaitherium*: P^4^; *Saghatherium*: distal part of M^2^). The jugal extends below the orbit but does not contact the lacrimal, because of the presence of a short intermediate process of the maxillary. The maxillary participates in the zygomatic apophysis, as a short medial process extending 1,5 cm posteriorly. There is no submaxillary fossa. The zygomatic apophysis diverges low with respect to the dental row. There is a robust (wide) ventral postorbital process of the zygomatic arch, that is made mostly by the jugal; this is reminiscent of primitive proboscideans such as *Numidotherium*. The jugal extends anteriorly up to the level of M^2^ parastyle. It forms a high and narrow vertical bony blade. The distal part of the zygomatic arch, broken in MNHN.F PM54, remains unknown in *Ocepeia*.

The parietal extends very anteriorly at the expense of the frontal. The area of the orbitotemporal fossa, medial to the zygomatic arch (sphenoid region), is not completely preserved, making difficult the identification of bones and their topographic relations. This is especially true of the orbital process of the frontal, the orbital process of the palatine, the orbitosphenoid, and the alisphenoid that is nearly entirely broken. The orbitosphenoid is small and located anteriorly, as in some primitive eutherians such as *Uchkudukodon nessovi* [45]; it extends dorsally above the postpalatine torus and the choana. The orbital process of the frontal is reduced in relation to the anterior development of the parietal; this is illustrated by a long parietal-orbitosphenoid suture and an anteriorly restricted orbitosphenoid-frontal suture. Among extant afrotherians, the condition of a parietal-orbitosphenoid suture associated with a reduced orbitosphenoid-frontal suture occurs in macroscelidids, and variably in *Orycteropus* [46]. Although broken, the alisphenoid was probably elongated, sharing a long suture with the parietal, in contrast for instance to *Phosphatherium* which has a long suture between the frontal and alisphenoid. Such extended relation of the parietal with at least the alisphenoid and possibly also with the orbitosphenoid is an original construction among the eutherians and the placentals (e.g., [46]).

At the base of the orbitosphenoid, five millimeters behind the choana and above it, there is a large semi-lunar and anteriorly oriented opening which might be either the optic foramen or the sphenorbital fissure (*f. lacerus anterius*), or both. Its large size would agree with a common opening for both the sphenorbital fissure and the optic foramen (and possibly also for the *foramen rotundum*) as in *Phosphatherium*; it resembles in shape that of *Phosphatherium*, but it is located much more anteriorly. According to Cox [46], the optic foramen is located anteriorly on the orbitosphenoid in most extant afrotherians, except in tenrecids. Among extant afrotherians, the fused sphenorbital fissure and optic foramen is a derived condition known in chrysochlorids [46]. A small subcircular foramen is located above the latter foramen, probably the ethmoidal foramen; it is located below an oblique bony crest corresponding to the posterior extension of the crista orbitotemporalis, as in *Phosphatherium*.

The distal part of basicranium is not elevated with respect to the anterior part of the skull (mesocranium), in contrast to *Phosphatherium* and other proboscideans. The squamosal dermal part is elongated, and shares long subhorizontal suture with the parietal. There is no contact between the squamosal and the frontal, as in primitive eutherians, and in contrast to *Phosphatherium*. The alisphenoid canal is probably absent in contrast to *Phosphatherium*; this is the primitive eutherian state. The postglenoid process is well-developed and higher than the posttympanic process of the squamosal which is closely appressed to the mastoid process. The external auditory meatus is widely opened ventrally (not compressed). The nuchal crests are well-developed and salient posteriorly behind the occiput. However, they are not so expanded posteriorly as in many primitive eutherians such as arctocyonids. The nuchal crests are formed by the supraoccipital which extends anteriorly for a short distance (3–5 mm).

#### Ventral view

The palate is wide. The occlusal surface of the cheek tooth series appears relatively large (e.g. breadth extension) with respect to the palatal area, which emphasizes the prominence of the dentition in *Ocepeia daouiensis* and indicates this is a megadont mammal. This is related to the herbivorous diet and this is another primate (anthropoid) convergence. Comparison of predictive equations of body mass based on various dental measurement (length, width, area) shows that the teeth are especially enlarged transversely. This derived morphology is well distinctive from many artocyonids such as *Tricentes* and *Arctocyon*; it is possibly autapomorphic.

The tooth row remains restricted mesially on the skull; it does not extend behind the orbit and the middle skull length. The cheek tooth row is aligned closely parallel to the longitudinal axis. Although broken apart in the known material, the premaxillaries are reconstructed as short and wide, by reference to the occlusion of the lower (anterior) dentition. No palatine fenestra are present in the maxillary. The posterior palatine foramen (*f. palatinus majus*) is located in front of M^2^. From this foramen, a well-developed palatine groove extends anteriorly up to the P^3^ level, similarly to primitive hyraxes; it housed the greater palatine nerve (V2) and blood vessels. The palatines are very short (comparable to the premaxillae) and posterior, in the primitive eutherian condition. They do not extend more anteriorly than M^2^, in contrast to *Eritherium* (M^1^, anterior part), and to *Pleuraspidotherium* (P^3^). They are even slightly more posterior than in *Hyopsodus*. The choana opens at mid-length level of M^3^. Its mesial rim is inflated as a lip-like structure indicating the presence of a postpalatine torus (attachment for *tensor palate*). The zygomatic process of the maxilla is well-developed laterally but short. It diverges between mid-length of M^2^ and M^3^, and it extends as a thin a bony blade medial to the anterior part of the jugal, and well behind M^3^. The temporal fossa and zygomatic arches are wide laterally and also long. The zygomatic process of the squamosal does not diverge very posteriorly, but at the level of the basisphenoid-basioccipital suture (i.e., 27% of skull length).

The pterygoid processes are well-developed, very long (e.g., versus *Pleuraspidotherium*) and widely separated. Consequently, the pterygoid fossa is wide and large, similarly to *Meniscotherium*. The large development of this fossa, which accommodates the medial pterygoid muscle, is in agreement with the enlarged angle of the dentary in the species. Just behind the pterygoid processes, opens the small foramen ovale within a longitudinal groove at the basis of the pterygoid crest. It is located anterior to the basisphenoid-basioccipital suture and to the postglenoid process, in contrast to *Pleuraspidotherium*. The pterygoid, basisphenoid and basioccipital are elongated, resulting in the overall long basicranial region of *Ocepeia*. The basiphenoid is especially long (e.g., versus *Hyopsodus*); it extends posteriorly to the pterygoid process. The basioccipital area is large and long, although wider than long. The presphenoid and vomer are poorly distinct. However, the CT scans suggest that the presphenoid was short, high and pneumatized, and that the vomer is crossed by a large canal.

The glenoid surface of the squamosal is poorly excavated, more extended transversely than longitudinally and opened anteriorly which makes possible wide antero-posterior movements of the dentary in *Ocepeia*. This morphology, known in Paleogene hyracoids and proboscideans, is paenungulate-like. The glenoid fossa is located partly on the braincase, in contrast for instance to *Arctocyon*. The postglenoid process is tall, higher than in *Phosphatherium.* It is mainly posterior to the glenoid fossa. The postglenoid foramen opens medially to the high postglenoid process; it is more medial than in *Phosphatherium*, in the primitive eutherian condition seen in *Daulestes*, *Maelestes*, *Zalambdalestes* [47]; *contra* [37]). The external auditory meatus is large, with a broadly concave suprameatal surface of the squamosal for the auditory tube. The posttympanic process of the squamosal is present but small. It is much smaller and lower than the postglenoid process, in contrast to *Phosphatherium*. It is widely separated from the paroccipital apophysis by the elongated mastoid region that is exposed ventrally (proboscidean feature).

The basioccipital is convex with a ventrally salient median keel which is also seen in several other condylarth-like taxa such as *Meniscotherium* and *Hyoposodus*. The median crest vanishes before joining the occipital condyles, but this is probably related to post-mortem damage. The hypoglossal foramen houses two small openings; it is smaller than the jugular foramen, and it is located more posteriorly from the jugular foramen than in *Phosphatherium*; it is 2.5 mm anterior to the occipital condyle. This eutherian-like construction differs from advanced proboscideans in which the two foramens tend to fuse. The hypoglossal foramen has an oval shape with a long axis parallel to the petrosal-basioccipital suture. The paroccipital process is low and located very posterior (behind the hypoglossal foramen and foramen magnum), as in *Phosphatherium*.

The braincase is wide, especially caudally (up to the alisphenoid-squamosal suture level), in contrast for instance to the arctocyonids and phenacodontids.

Periotic (Figs. 5–6). Both the right and left petrosals are present in MNHN.F PM45, although the structural details in ventral view are poorly preserved. The petrosal of *Ocepeia* is large and especially long: 1) the pars cochlearis is small and very anterior with a strong rostral tympanic process extending to the level of the anterior margin of the postglenoid process; 2) the pars mastoidea is very long and widely exposed ventrally between posttympanic (squamosal) and paroccipital processes (both separated from about 10 mm). The enlarged pars mastoidea is a remarkable feature of *Ocepeia* that is shared with proboscideans. The pars cochlearis and promontorium are ovoid, long, and slightly oblique with respect to the longitudinal axis. The promontorium extends posteriorly to the postglenoid process level, and it is more ventral than the basioccipital. It is inflated and located anteriorly. Its almond shape resembles that of *Hyopsodus* (p). The promontorium has a smooth surface and it bears a stout ridge that is oblique postero-laterally. This ridge is bounded antero-laterally by a sulcus probably for the internal carotid artery (d). The promontorium sulcus for stapedial artery is absent. The rostral tympanic process of the petrosal is salient anteriorly to the promontorium. The epitympanic wing of the periotic medial to the promontorium is absent (p). The fenestra vestibuli (oval window) is very small (area  = 0.312 mm^2^), and much smaller than the fenestra cochleae. The size of the fenestra vestibuli of *Ocepeia daouiensis*, and correlatively of its stapes, is well below the regression line for fenestra vestibuli area versus body mass in modern mammals [48]. In fact, the small size of the fenestra vestibuli of *Ocepeia daouiensis* is in close proportion to *Numidotherium koholense* [49]. This is a possible derived feature shared with paenungulates, although sirenians show an early divergent trend to hypertrophy of the ear ossicles. It should be noted that the small size of the f. vestibuli correlates with that of the promontorium and inner ear (see below). The fenestra vestibuli is elliptical with a high stapedial ratio (2.05) which is generalized in placentals [42], [50].

The fenestra cochleae (round window) is located postero-medial to the fenestra vestibuli, in the eutherian state. It is larger than the fenestra vestibuli, but smaller than the jugular foramen. It is vertical and elongated transversely (p), and it faces posteriorly as in *Phosphatherium* and the primitive eutherian or placental condition. The presence of the cochlear canaliculus (*aquaeductus cochleae*) is evidenced by the CT scan observations. It opens, in the primitive condition, ventro-medially in the jugular fossa, below and more medially than the cochlear fenestra; this differs from *Phosphatherium* where it is more ventral and anterior. This canal is unusually large in section and its opening is dilated transversely. The stylomastoid foramen for *n. faciale* (VII) is distinct postero-laterally to the fenestra cochleae. The jugular foramen (*f. lacerus posterius* for glossopharyngeal nerve, IX) is the largest foramen of the basicranium; it is located just behind the promontorium. Its shape is oval with an oblique postero-lateral to antero-medial long axis. The caudal tympanic process of the petrosal is well-developed, which is generalized in eutherians according to McPhee [51]. However, a large and inflated caudal tympanic process is a trait of the Paenungulata, known for instance in *Phosphatherium* and *Numidotherium*.

The tympanohyal is large. The tegmen tympani is remarkably inflated and pneumatized; it forms a large and robust barrel-like bony structure located antero-lateral to the promontorium, and it partially covers the epitympanic recess. An inflated tegmen tympani is a derived feature among Eutheria [51], [52], [53]. It was considered as an ungulate [53] and a tethytherian feature [54], [55]. The barrel-vault like shape of the tegmen tympani is known in tethytheres. The hyperinflated tegmen tympani is known in *Meniscotherium, Mesonyx*, anthracotheriids, hippopotamids and cetaceans [56], [57]. The tympanohyal is large. No trace of an ossified tympanic bulla is seen in MNHN.F PM45.

In dorsal view (CT scan 3D model: Fig. 6C, Video S2), the internal meatus and the subarcuate fossa are well distinct and comparable in size. The subarcuate fossa is moderately large and deep. The internal meatus shows two distinct openings separated by a short crista transversa: the f. acusticum superius for the facial nerve (VII), and the f. acusticum inferius for the vestibulo-cochlear nerve (VIII). The *f. acusticum inferius* is posterior and circular, and it shows distinct link with the cochlea (cochlear canal) close to f. cochleae. The *f. acusticum superius* is anterior and elongated (bean-like outline). In dorsal to dorso-lateral view, there is a large opening that extends medially as a canal in the inflated tegmen tympani and that might correspond to the foramen for the ramus superior of the stapedial artery. Its opening is as large as that of the internal acoustic meatus.

Inner ear (Figs. 6B, 7). The endocast of the bony labyrinth of *Ocepeia daouiensis* was successfully reconstructed with the help of the computed X-ray micro-tomography analysis of the skull MNHN.F PM45. We reconstructed the whole inner ear endocast of the left petrosal (Fig. 6B), as well as also the semicircular canals of the right petrosal. The perfect alignment of the semicircular canals of the right and left petrosal in lateral view indicates that the bony labyrinth is well preserved and not distorted in the skull MNHN.F PM45. The detailed description of the bony labyrinth system of *Ocepeia daouiensis* will be presented separately with a functional analysis (ms in prep); here we report a comparative description of the main morphological traits of the inner ear that are of systematic and phylogenetic values. The number of coils of the cochlea measured following method of West [43] is 2.12 (765°). Most placentals have at least 2 turns, with some exceptions such as erinaceids and some sirenians [58]; Cretaceous eutherians are plesiomorphic with fewer turns (1 to 1.5 turns) than crown placentals [58], [59]. *Numidotherium* has a smaller number of turns than *Ocepeia* according to Court [60] (1.5) and Benoit et al. [61] (1.62). The spiral plane of the cochlea is much more oblique antero-dorsally with respect to the horizontal plane (50–60°) than in Mesozoic eutherian mammals. The coiling is subplanar as in the primitive eutherian condition seen for instance in the zhelestids [59]. In the coiling plane, the spiral turns are well separated from each other as in primitive Late Cretaceous eutherians such as zhelestids [59]. The ventral coil has a diameter comparable to the dorsal coil, and the apex of the cochlear canal (helecotrema) is large. This is distinct from *Notostylops*, but of unknown polarity among eutherians. The secondary bony spiral lamina is present on the basal coil (340 to 360° from f. vestibuli). In several late Cretaceous eutherians [58] and in *Notostylops* [50], it is primitively restricted to half of the basal coil. However, the secondary bony spiral lamina extends to the whole basal coil in some other primitive Mesozoic eutherians such as zhelestids, as in *Ocepeia*. The anterior semicircular canal is the larger semicircular canal as in most eutherians. The posterior segment of the lateral semi-circular canal is coalescent with the posterior ampula of the posterior semi-circular canal on a short distance from the vestibule. However, the lateral semi-circular canal separates from the posterior ampula before its ends, and the lumen of both lateral semicircular canal and posterior semicircular canal PSC remains distinct, which indicates that there is no true secondary crus commune. The absence of a secondary crus commune is a generalized condition shared by crown placentals in contrast to stem eutherians that have a secondary crus commune [59]. However, it is noticeable that several placentals retain a secondary crus commune, such as the aardvark, some carnivorans, the primitive macroscelidean *Chambius* [61], and the primitive proboscidean *Numidotherium* [62]. The crus commune of the anterior semicircular canal and posterior semicircular canal is less long than in generalized eutherians such as zhelestids and *Zalambdalestes* [59].

#### Occipital view

The supraoccipital is well-developed. Transverse and longitudinal CT sections show that it is very thickened and pneumatized (Fig. 4). It extends anteriorly below the parietal, not far from the level of the basisphenoid-basioccipital suture. The foramen magnum is large, comparable in size to “condylarths” such as *Hyopsodus*; it is wider than high. The occipital condyles are salient posteriorly, and they join ventrally as in *Meniscotherium* (and *Zalambdalestes*). The nuchal crest is salient posteriorly all around the occiput; it does not form the peculiar posterior upper expansion seen in many eutherians and primitive placentals such as arctocyonids, pleuraspidotheriids, pantodonts, pantolestids and others. Below the nuchal crest, the occiput appears slightly inclined anteriorly (eutherian trait).

The occipital surface is depressed between the nuchal crest and the condyles. There is a small condylar foramen. The paroccipital process is small. There is no mastoid exposure in the occipital side (amastoid structure).

*Skull measurements: see* *Table 1* *.*

**Table 1:** *Ocepeia daouiensis,* skull measurements, specimens MNHN.F PM45 and PM54 (mm).

Skull length estimated from reconstruction	∼90
Max length preserved MNHN.F PM45 (premaxillae broken)	83
Length of muzzle MNHN.F PM45	>30
Height of muzzle at anterior border of orbit MNHN.F PM54	25
Length from choana to anterior side MNHN.F PM54	42
Length from choana to posterior side MNHN.F PM45	48
Palate width at M^2^ (paracone) transverse level MNHN.F PM45	22×2 = 44
Palate width at M^2^ (paracone) transverse level MNHN.F PM54	44
Palate width lingual to M^2^ MNHN.F PM45	8×2 = 16
Palate width lingual to M^2^ MNHN.F PM54	15
Maximal width estimated from reconstruction	∼50
Minimal width (mid skull constriction) MNHN.F PM45	16
Nuchal width MNHN.F PM45	30
Nuchal height MNHN.F PM45	23
Length of sagittal crest MNHN.F PM45	40
Length of frontal (dorsal view) MNHN.F PM45	14
Foramen magnum Length x Height MNHN.F PM45	10×9

### Description: Upper dentition (Figs. 3, 8–10)

Remark - The lower dentition of *O. daouiensis* is described by Gheerbrant et al. [26], [39]. The upper dental formula of *O. daouiensis* identified here is I^1?^, I^2^, I^3?^, C^1^, P^3^, P^4^, M^1^, M^2^, M^3^, which is identical to lower dental formula. In most of the available material, the teeth are unworn and the degree of wear of all cheek teeth is comparable, suggesting that permanent teeth erupt nearly at the same time (i.e., in a short time span), except for the mast molar that is the last tooth to erupt (e.g., MNHN.F PM45). The enamel is slightly wrinkled.

#### Incisors (Fig. 8)

A broken piece of left premaxillary of the specimen MNHN.F PM54 bears an enlarged root belonging to a broken incisor that lacks the crown (Fig. 8). The root is hypertrophied and compressed laterally; it is larger than the canine root (L = 7 mm). It indicates an enlarged incisor that is identified as a left I^2^ (e.g., no trace symphysis in associated preserved bone). The crown was probably procumbent. It is inferred that this is the larger upper incisor as in early proboscideans such as *Numidotherium* and *Moeritherium*.

As in the lower dentition known in *O. daouiensis* [26], the third upper incisor was probably very small and vestigial.

#### Canine (Figs. 3, 10)

The canine preserved in MNHN.F PM54 is separated from the I^2^ by a small diastema. The canine is stout, low, but sharp. The root was inflated and larger than the crown. The crown is convex laterally and concave lingually. It bears a small anterior crest and a longer (higher) distal crest. A faint trace of lingual cingulum is present, but it is much smaller than in the lower canine which is more anthropoid-like in this respect.

#### Premolars (Figs. 9–10)

The two first premolars are absent, as in the lower dentition. They are not replaced by a significant diastema in the tooth row: P^3^ is very close to C^1^.

P^3^ and P^4^ are sub-molarized with a developed protocone, three roots, and a more or less transverse occlusal outline. As a consequence, the whole cheek dentition is rather homodont.

The labial cingulum is inflated, especially at anterior and distal ends. Traces of stylar cusps are present, most distinctly as a metastyle, and more variably as a D cusp. The paracone is the main labial cusp: it is large, low and nearly as wide as long. It bears long V-shaped shearing anterior and posterior crests that are underlined by a distinct wear facet. Shearing function with trigonid of occlusal relative teeth P^3-4^ was enhanced in *Ocepeia*. There is no trace of metacone. The protocone is much smaller than the paracone. It bears symmetric and well-developed pre- and post-protocristae that enclose a well delimited protofossa. The preprotocrista joins a very small parastyle; the postprotocrista, well distinct in both P^3^ and P^4^, joins the postparacrista. One or more variable cuspules are inflated on the postprotocrista behind the protocone. Their position is reminiscent of the molar metaconule. A small but distinct transverse crest crosses the protofossa in its anterior part and links the internal flank of paracone and protocone. The lingual flank of the protocone is convex. A very slight enamel bulging of the crown below and behind the protocone of P^4^ suggests trace of postcingulum.

P^3^ and P^4^ are very similar in morphology, but the former is slightly smaller, less transverse and has a smaller protocone.

#### Molars (Figs. 9–10)

M^1-3^ are bunoselenodont, with a W-like (selenodont) ectoloph that is linked to an inflated mesostyle. They lack a hypocone.

The styles are remarkably well-developed and inflated, and located labial to paracone and metacone. Parastyle and mesostyle are the larger styles, they form inflated cusps on the labial side of the crown. Additional small stylar crests and cuspules are variably present in front of the paracone and metacone (e.g., D cusp). The stylar shelf is present but not so wide. The paracone and metacone are low and linked by a dilambdodont ectoloph that is typically joined to the large mesostyle. The two arms of the centrocrista are united labially just before joining the mesostyle.

The preparacrista joins the parastyle; the postmetacrista, well-developed, joins the metastyle. The labial flank of the paracone and metacone is convex. The paracone is larger than the metacone, and more so from M^1^ to M^3^. The protocone is remarkably low and expanded mesio-distally; its crests are widely divergent. Its labial flank is convex. The protofossa is vast, and especially long, and shallow. A very short transverse crest is present at the base of the lingual flank of the paracone. The preprotocrista joins the parastyle below the preparacrista. The postmetacrista joins the base of the postmetacrista below the metacone. The paracingulum is much more developed than the metacingulum. A metaconule is more or less inflated on the postprotocrista where it is salient disto-lingually; it is more developed on M^1^ than on M^2^. The metaconule is doubled lingually by a small additional conular cuspule. There is also a small paraconule on the preprotocrista; it is located more labially than the metaconule. The lingual flank of the protocone is strongly canted labially. A more or less distinct trace of continuous lingual cingulum is present on M^1–3^, around the protocone, but it lacks a hypocone. Three roots are present, including in M^3^.

The selenodont pattern of the ectoloph is functionally underlined by well-developed related semi-lunar shearing facets such as wear facets 3 and 4, 1a and 1b (nomenclature of Crompton [63]).

The occlusal outline is more or less square in M^1^, and wider transversely in M^2^ and M^3^. The molar size increases slightly from M^1^ to M^3^.

*Dental measurements: see* *Tables 2* *–* *3* *.*

**Table 2:** *Ocepeia daouiensis*, dimensions of upper dentition: upper teeth (mm).

	C^1^			P^3^		P^4^		M^1^		M^2^		M^3^	
Specimen	L	W	H	L	W	L	W	L	W	L	W	L	W
PM54 right	5,2	5,1	*8	6	6,4	5,5	7,6	7	8	6,8	9	6,4	8,5
PM54 left	5,7	5	?	5,6	6,3	5,6	7,3	7,1	8,1	7	9	6,2	8,5
PM45 right	?	?	?	*5,6	6	5,3	7,5	6,5	8	6,1	8,5	?	?

L: Length; W: Width.

*Estimated measurements.

**Table 3:** *Ocepeia daouiensis*, dimensions of upper dentition: Length (L) of upper tooth row (mm); r: right; l: left.

Specimen	PM45 r	PM54 r	PM54 l
L C-M^3^	?	37	40,5
L P^3^-M^3^	?	30,9	30,7
L P^4^-M^3^	?	25	25,2
L P^3^-M^2^	*23	24,5	25
L P^4^-M^2^	17,5	18,5	19
L P^4^-M^1^	11,3	12	12
L M^1-3^	?	20	20,1
L M^2-3^	?	13,2	13,5
L M^1-2^	12,5	13,1	14
L P^3-4^	10,6	11,8	11

*Estimated measurements.

### Body mass estimation (Table 4)

Our estimations of the body mass of *Ocepeia daouiensis* (Table 4) vary noticeably with the skeletal measurements: 1) Body mass estimates are higher based on anterior molars (M3 provides the smallest estimates); 2) Body mass estimates based on tooth area are higher than those based on tooth length.

**Table 4:** Body mass estimates of *Ocepeia daouiensis* (in grammes).

Measurements base	All ungulates	Selenodonts	Selenodont browsers
Area M1*	9306.15	8769.99	7881.27
Area M2*	7556.50	7130.07	6358.69
Area M3*	5756.35	5188.77	4830.70
Length M1*	6924.72	6934.83	6357.63
Length M2*	4394.59	4037.88	3691.52
Length M3*	**3484.46**	2741.06	2687.64
Length M1-3*	**4043.51**	3846.18	3417.84
Skull length**	**2950.34**	—	—

*Mean of upper and lower teeth;

**estimation  =  minimal size. Predictive allometric equations from [64], [67] for all ungulates, selenodonts, and selenodont browsers. The best (lower) estimates are in bold (mean for all ungulates  = 3500 g.; see text).

For ungulates, it is agreed that tooth lengths are better predictors than tooth widths or areas that vary more with dietary specialisations [64]. Moreover, body mass estimates based of skull measurements are considered more reliable for primates [65].

Body mass estimates of *Ocepeia daouiensis* based on M1 and M2 area are in the higher range (predictive equation for all euungulates (Artiodactyla, Perissodactyla), mean M1 and M2 = 8.5 kg) and are obviously overestimated. This is related to the fact that M1 and, less markedly, M2 are larger in *Ocepeia daouiensis* relative to modern euungulates on which are based the predictive equations [64]; modern euungulates have a greater size ratio from M1 to M3 than in *Ocepeia*. The overestimation of the body mass based on anterior molars seems to confirm that *Ocepeia* is a megadont form with large anterior molars (see description).

Body mass estimate based on the skull length of *Ocepeia daouiensis* is in the lower range (2.9 kg) and is close to the estimates based on the length of M1-3 and M3. The relative size of M3 (larger relative to M2 and M1) in *Ocepeia daouiensis* is closer to that of modern mammals; this might explain why M3 provides more reliable body mass estimates that are close to those based on the length of M1-3 and of the skull. The mean body mass estimate of *Ocepeia daouiensis* based on the length of M1-3, M3 and the skull is 3.5 kg (predictive equations for all ungulates). It is comparable to extant hyraxes and to *Meniscotherium chamense* (3.4 kg [67]) which are close in skull size, and in dental morphology and specialisations.

Kondrashov & Lucas [66] have recently shown than dental measurements of early condylarths such as the phenacodont *Tetraclaenodon puercensis* “*pliciferus*” (Torrejonian), of skull size (110 mm) close to that of *Ocepeia*, give much larger body mass estimation (14 kg) than postcranial measurements (3.2 kg), similarly indicating they are megadont primitive ungulates. The cheek dentition of *Ocepeia* is actually proportionally much larger with respect to the palate and skull size than in *Tetraclaenodon puercensis*.

### Reconstruction of the skull and the head of *O. daouiensis*: Figs. 11–13

The reconstruction of the skull *O. daouiensis*, made by 3 of us (EG, FG, CL), is based primarily on the most complete specimen, MNHN.F PM45. However, because this specimen is incomplete and slightly crushed dorso-ventrally, some details of the reconstruction (e.g., dentition, I^2^ C^1^, M^3^) were completed based on comparison with MNHN.F PM54. However, the reconstruction of the whole skull is not a chimera of the two specimens. It is indeed important to note that specimen MNHN.F PM45 is more distorted than other specimens, and it is a probable female with much more gracile overall morphology and smaller size than MNHN.F PM54. Hence, because of the sexual dimorphism of *O. daouiensis*, our reconstruction is only partially representative of the species, i.e. it is not representative of male individuals which would appear significantly more robust (with stronger sagittal and temporal crests), as exemplified by MNHN.F PM54.

The lower jaw reconstructed in Figure 12 is based primarily on specimen PM41, and other specimens for the anterior dentition (see [26]).

The reconstruction of the skull was made using the 3D models computed from tomographic data and processing (by FG) with help of programs Mimics Innovation Suite (Materialize, release 16), VG studio Max (Volume Graphics, release 2.2) and Cinema 4D (Maxon, release 13). The 3D model of the specimen MNHN.F PM45 was segmented following bone sutures, lines of distortion and breaks and best preserved part; segments were re-assembled after correction of distortions in the three main anatomical orientations (Fig. S2), by comparison with available material and using natural symmetry in the arrangement of skull bones.

The main difficulty in our reconstruction was the plastic distortion of individual bone that could not be retrodeformed; this especially true for the dorso-ventral compression of MNHN.F PM 45 that could not be evaluated precisely, and for which we propose an estimated correction in the Figures 11-13 based on the comparison with MNHN.F PM 54.

The final drawing of the reconstruction was based on the reconstructed 3D digital model (Fig. S2), with help of the original specimens and their casts, of superposed photographic views and camera lucida drawings (e.g., occlusal view of dentition, ventral view of petrosal), and of osteological measurements. Drawings (by CL) were made by hand and on computer with help of specialized computed programs (Adobe Creative Suite 6).

### Comments

Many features of *Ocepeia daouiensis* are generalized eutherians traits (Table 5), which indicates it is very primitive.

**Table 5:** *Ocepeia daouiensis*, main primitive (eutherian) features of the skull, dentition and lower jaw.

Palatines very short and posterior (posterior to M^2^)
Large lacrimal facial process
No contact between frontal and maxillary (rostrum)
Infraorbital foramen anterior (above P^3^) and small
Jugal extended anteriorly on the orbit at the expense of the maxillary
Orbit posterior
Zygomatic arches not strongly flared laterally
Wide pterygoid fossa
Alisphenoid canal absent
Postglenoid foramen medial to postglenoid process
Hypoglossal foramen present and double
Hypoglossal foramen far distal to jugular foramen
Occiput slightly inclined anteriorly
Strong nuchal and sagittal crests
Almond pointed shape of promontorium
Fenestra cochleae and aquaeductus cochleae present
Fenestra cochleae postero-medial to the fenestra vestibuli
Fenestra cochleae vertical and elongated transversely
Fenestra cochleae faces postero- laterally
Epitympanic wing of the periotic medial to the promontorium absent
Cochlear canal (aquaeductus cochleae) opens ventro-medially in the jugular fossa
Well-developed caudal tympanic process of the petrosal
Petrosal subarcuata fossa present, large and deep
Inner ear: Spiral turns of the cochlea widely separated
Inner ear: Subplanar coiling of the cochlea
Inner ear: Large anterior semicircular canal
Upper molars: Wide stylar shelf and presence stylar cusps (incl. D cusp)
Upper molars: Parastyle well-developed and mesio-labially located
Upper molars: Paracrista strong joining labially the parastyle
Upper molars: conules distinct
Upper molars: No hypocone
P^3-4^: Metacone absent
P^3-4^: No trace of transverse loph
P_3-4_: Simplified
Canine large
Lower molars: Paraconid present, paracristid sharp, trigonid not compressed
Lower jaw: No coronoid foramen

Several symplesiomorphic resemblances are noted with “condylarths” such as arctocyonids (e.g., orbit location and construction) and hyopsodontids. The arctocyonids such as *Arctocyon* also share some remarkable derived features such as the shortened frontal, although the structure is more derived in *Ocepeia*, with for instance a strongly reduced orbito-temporal process of the frontal. However, the skull of *Ocepeia daouiensis* has closest resemblances with pleuraspidotheriids, including in derived features such as the elongated parietal and basicranium, the large pterygoid fossa, and the basioccipital shape. The lower jaw of *Ocepeia* also resembles pleuraspidotheriids in the wide angle. However many other differences, including divergent specialized traits, show that this is result of superficial resemblances related to convergences and symplesiomorphies.

Interestingly, a dental resemblance of *Ocepeia* is noted with dilambdodont pantodonts such as *Haplolambda* and *Leptolambda*. It includes the selenodont molars, but also the absence of hypocone, presence of a continuous lingual cingulum on upper molar, presence of a paraconid on lower molars, overall shape of the dentary with enlarged angle and deep and robust corpus, and short anterior dentition and symphysis. Similar skull features are also noted such as the flat and long skull roof, widely fused orbito-temporal fossa, and the long parietal and sagittal crest. However, *Ocepeia* and pantodonts differ by both derived and primitive features (orbit position, dental formula, zygomatic arch composition, etc…), indicating well distinct lineages that converged for some features.

Some skull features of *Ocepeia daouiensis* are known in semi-aquatic mammals such as pantolestids: the short muzzle, the absence of postorbital process and related widely confluent orbito-temporal fossa, the long and flat skull roof, the skull shape broader than high, the strong and posteriorly salient lambdoid crests. However, many other features of *Ocepeia* are well distinct from pantolestids, for instance the small infraorbital foramen, the short frontal, the lower jaw morphology and the distinctive dentition.

The skull and dentition of *Ocepeia daouiensis* are characterized by a remarkable combination of primitive and derived features. The most remarkable derived features are summarized in Tables 5–7.

**Table 6:** *Ocepeia daouiensis*, main derived features of the skull and lower jaw (* anthropoid-like feature).

Feature description	Taxonomic rank	K # in matrix
Petrosal: Fenestra vestibuli elliptical with high stapedial ratio	Placentalia	164–1
Petrosal, inner ear: Secondary bony spiral lamina present on the basal turn	Placentalia, some primitive eutherians	174–1
Petrosal, inner ear: Number of turns of the cochlea ≥2	Placentalia	175–2
Petrosal, inner ear: Large angle of spiral plan of cochlea and horizontal plan	?Placentalia	-
Petrosal: Fenestra cochlea oriented posteriorly	?Placentalia	-
Petrosal, inner ear: No secondary crus commune	Placentalia	-
Petrosal, inner ear: Crus commune moderately long	Placentalia	-
Small coronoid fossa present	Paenungulata	63–1
Wide nasal cavity	?Paenungulata	126–1
Very short maxillary process developed in the orbit rim (short bony blade between lacrimal and jugal)	Paenungulata	136–1
Jugal high with small but distinct postorbital ventral process at maxillary suture	?Paenungulata	145–1
Amastoidy	Paenungulata	161–1
Zygomatic process of the maxilla well-developed laterally, extended caudally on the medial part of the jugal	Paenungulata	-
Caudal process of the petrosal large and inflated	Paenungulata	-
Glenoid surface of the squamosal poorly excavated, wider than long, and opened anteriorly	Paenungulata or Afrotheria	-
Enlarged (long) pars mastoidea	Proboscidea or autapomorphy	162–1
Lower jaw: Mandibular symphysis probably fused	Autapomorphy*	52–1
Lower jaw: condyle significantly higher than the tooth row	Autapomorphy	59–1
Extensive pneumatization of the skull bones	Autapomorphy	118–2
Short and wide rostrum (premaxilla, maxilla, nasal)	Autapomorphy*	120–3
Short frontal, with reduced orbito-temporal process	Autapomorphy	141–1
Meso- and postero-cranial region elongated, including parietal, alisphenoid, pterygoid, basisphenoid, periotic (pars mastoidea)	Autapomorphy	-
Parietals with two oblique bony ridges diverging anteriorly from the mid part of the sagittal crest	Autapomorphy	-
Lower jaw: corpus inflated	Autapomorphy	-
Lower jaw: broad and round posteriorly projecting angular process	? (“ungulates”)	62–1
Fenestra vestibuli very small	?	163–1
Tegmen tympani hyper-inflated and pneumatized, forming large and robust barrel-like bony structure	?	169–1
Sulcus for the internal carotid artery on promontorium	?	-
Jugular foramen large	?	170–1
No postorbital process of the frontal	?	148–1
Lower jaw: condyle with two articular facets	?	-

Numbers refer to those of the matrix analyzed in this work (see Text S1, part II).

**Table 7:** *Ocepeia daouiensis*, main derived features of the dentition (with presumed rank of apomorphy; * anthropoid-like feature).

Feature description	Taxonomic rank	K # in matrix
Lower molars: Entolophid incipient	Paenungulata	34–1
Upper molars: Mesostyle well-developed in M^1-3^	Paenungulata	98–1
Upper molars: Mesostyle labial	Paenungulata	99–2
Upper molars: Ectoloph selenodont, centrocrista linked to mesostyle	Paenungulata	101–2
M1<M2<M3	?Paenungulata	111–1
I_3_ vestigial	Proboscidea or autapomorphy	7–1
No diastema between C1 and P3	Autapomorphy*	10–0 1(r), 69–1 (r)
P1-2 absent	Autapomorphy*	11–3, 13–3, 72–1; 74–1
Lower molars: Hypoconulid reduced and lingual in M_1-2_	Autapomorphy	41–1, 42–1
I^2^ enlarged	Proboscidea or autapomorphy	67–1
C_1_ stout and anthropoid-like (lingual cingulum, asymmetrical labio-lingual profile)	Autapomorphy*	-
Lower molars: Protoconid and hypoconid selenodont-like with semi-lunar wear facets	Autapomorphy	-
Upper cheek teeth more or less homodont	Autapomorphy	-
Lower incisors compressed with root wide and short	Autapomorphy*	-
Dentition megadont	Autapomorphy*?	-
Upper molars: weak lingual cingulum, continuous around the protocone	Autapomorphy?	-
Lower molars: Paraconid median in M_3_	?	-
Molars bunodont	? (“ungulates”)	25–1, 88–1

Numbers refer to those of the matrix that is analyzed in this work (see Text S1, part II).

These derived features are of different taxonomic rank value. Few but significant ones are placental traits. They concern mostly the inner ear structure, such as the cochlea with at least two turns. Other derived traits are mostly ungulate-like (i.e., ungulate-grade like), or paenungulate-like, or autapomorphic features.

Paenungulate traits are much more significant than the phenacodont-like features previously discussed for *Ocepeia* [39]. They include skull and dental features (Tables 6, 7) that are discussed in the phylogenetic analysis.

Other derived features of *Ocepeia* are autapomorphies. Some of them are striking anthropoid-like convergent traits (* in Tables 6, 7): I_1–3_ compressed, canine shape, short rostrum including short and fused mandibular symphysis, and short anterior dentition (length of I_1–3_  =  length of C_1_), and loss of P1-2 without significant diastemata, and also the inflated mandibular corpus.

***Ocepeia grandis*** n. sp. Gheerbrant

(Figs. 14–20)

**Figure 14:**
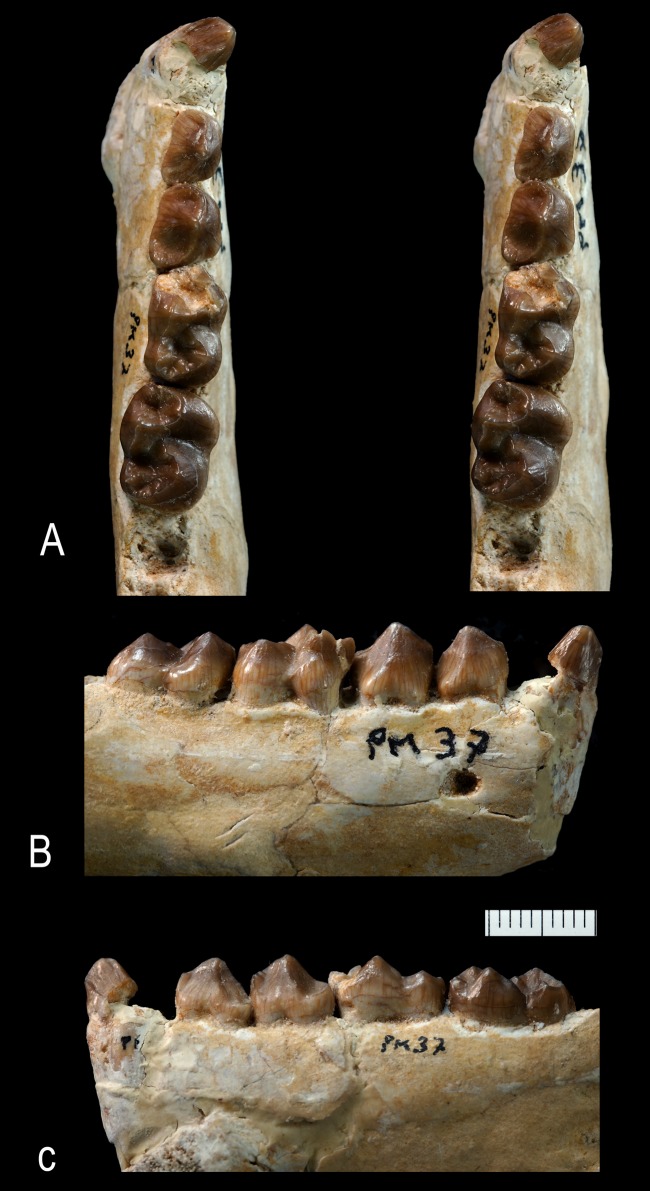
*Ocepeia grandis* n. sp., Thanetian (Phosphate level IIa), Sidi Chennane, Ouled Abdoun Basin, Morocco. Holotype, MNHN.F PM37, fragment of right dentary bearing C^1^, P^3–4^, M^1–3^, in occlusal (stereophotograph, a), labial (b) and lingual (c) views. Scale bar in millimeters. Scale bar:10 mm.

**Figure 15:**
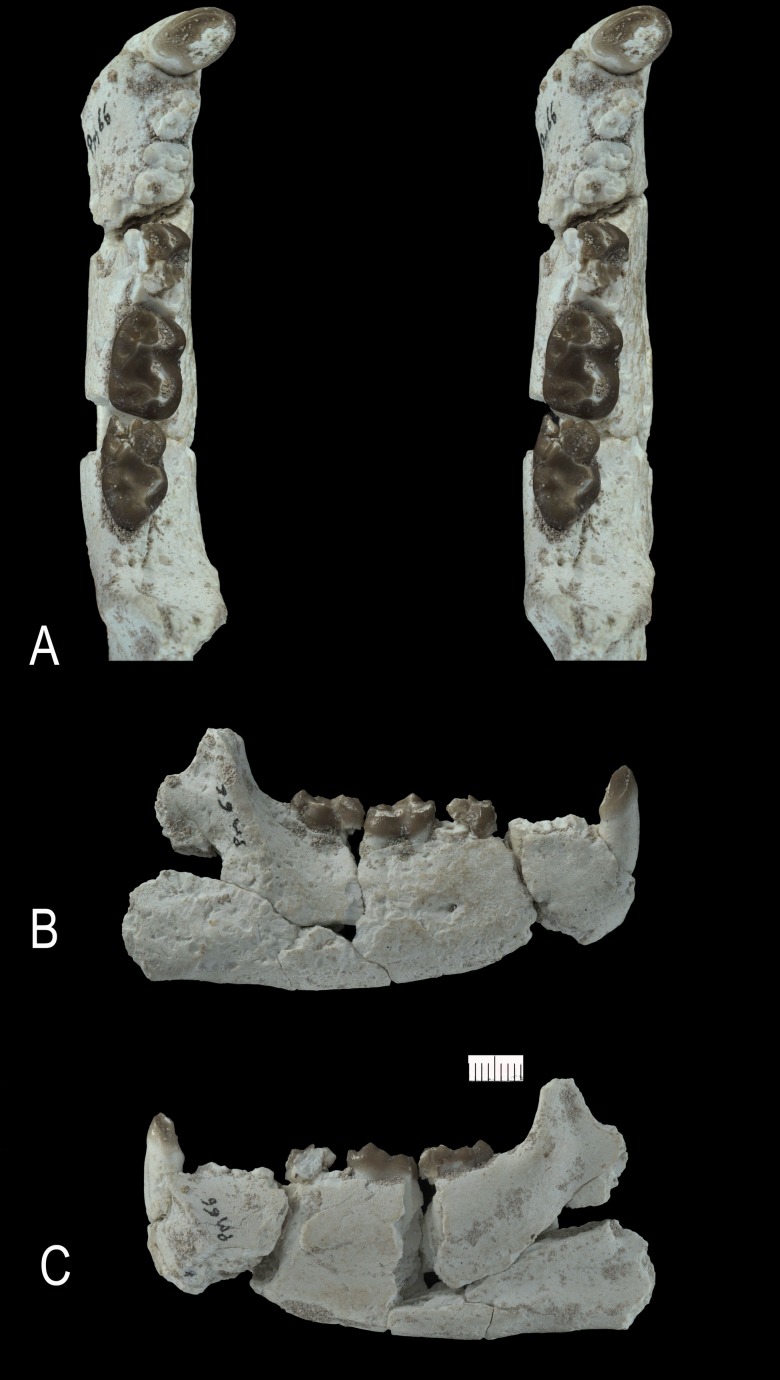
*Ocepeia grandis* n. sp., Thanetian (Phosphate level IIa), Sidi Chennane, Ouled Abdoun Basin, Morocco. PM66, right dentary with M_2–3_, alveoli for C_1_, P_3–4,_ M_1_, in occlusal (stereophotograph, a), labial (b) and lingual (c) views (private collection; cast MNHN). Scale bar in millimeters. Scale bar:10 mm.

**Figure 16:**
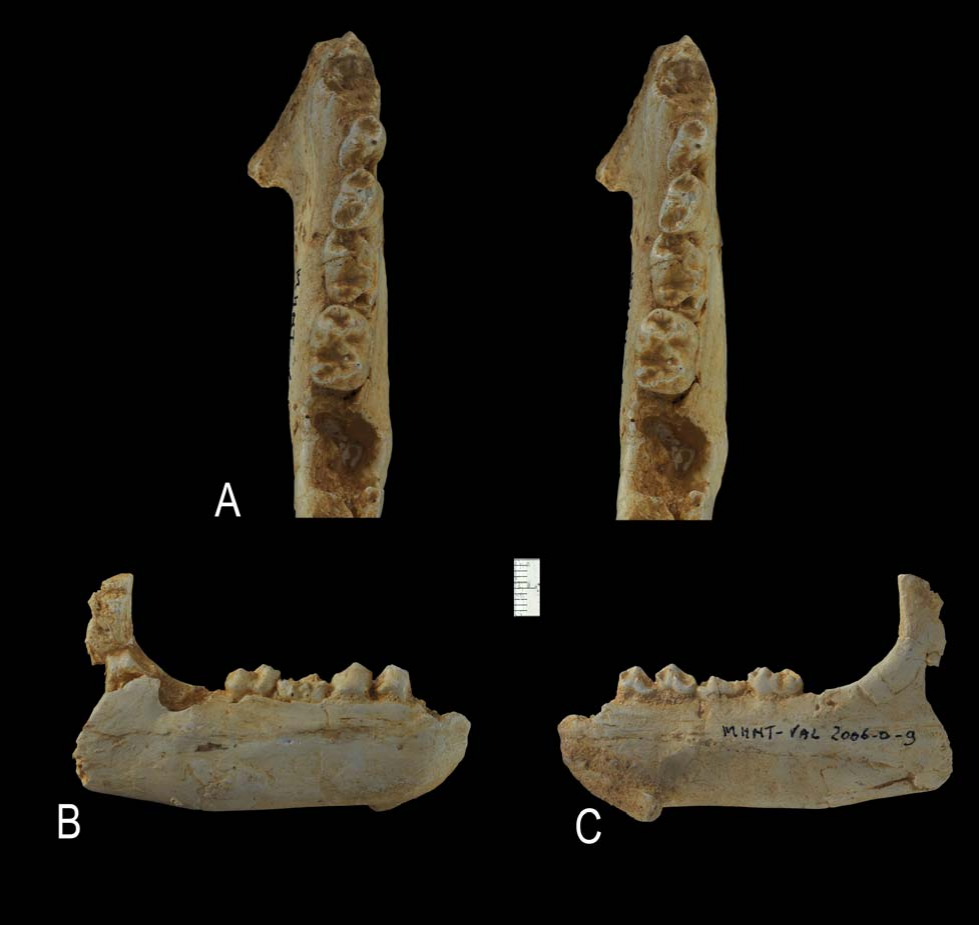
*Ocepeia grandis* n. sp., Thanetian (Phosphate level IIa), Sidi Chennane, Ouled Abdoun Basin, Morocco. MNHT.PAL.2006.09, right dentary with P_3–4_, M_1–2_, alveolus for C_1_ and mandibular symphysis, in occlusal (stereophotograph, a), labial (b) and lingual (c) views (coll. of the Museum of Toulouse). Scale bar:10 mm.

**Figure 17:**
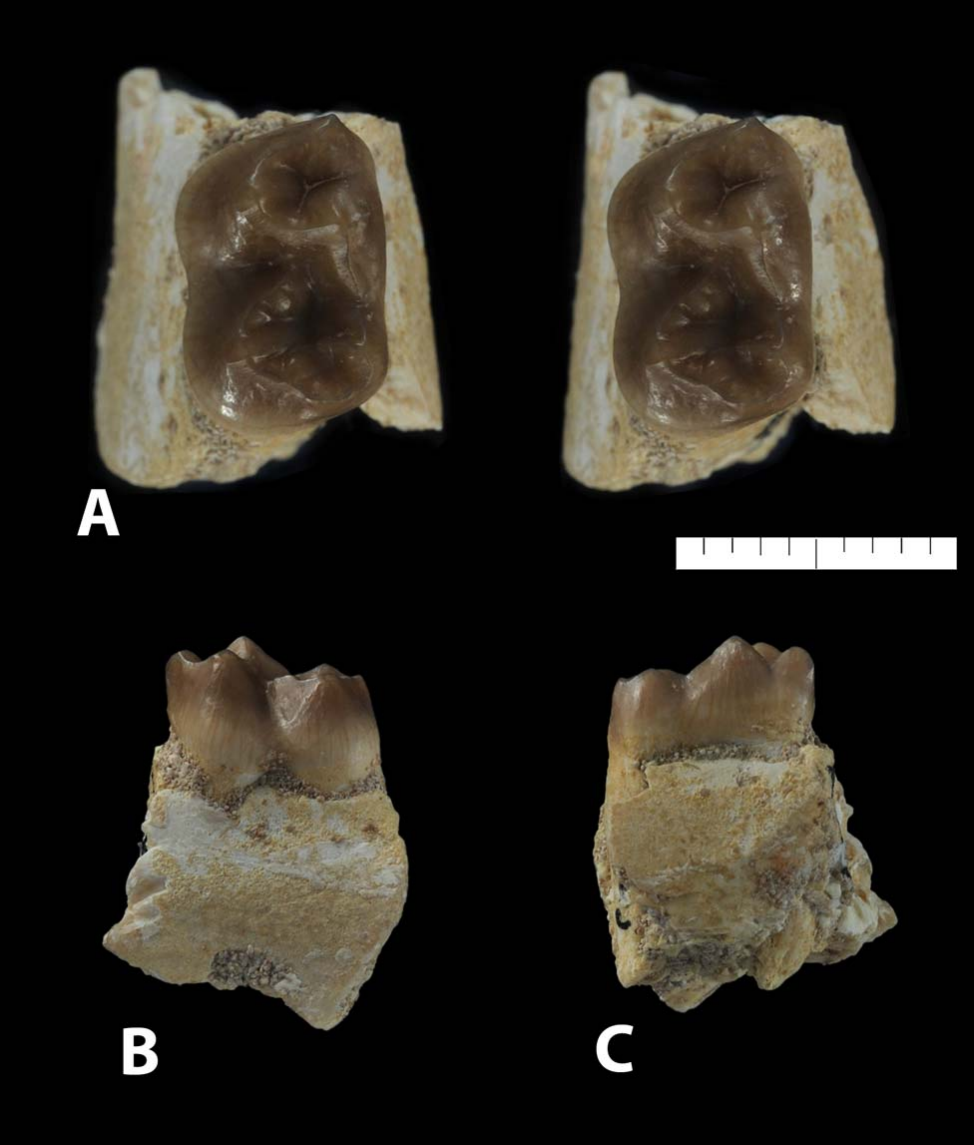
*Ocepeia grandis* n. sp., Thanetian (Phosphate level IIa), Sidi Chennane, Ouled Abdoun Basin, Morocco. MNHN.F PM34, fragment of left dentary with M_1_, in occlusal (stereophotograph, a), labial (b) and lingual (c) views. Scale bar:10 mm.

**Figure 18:**
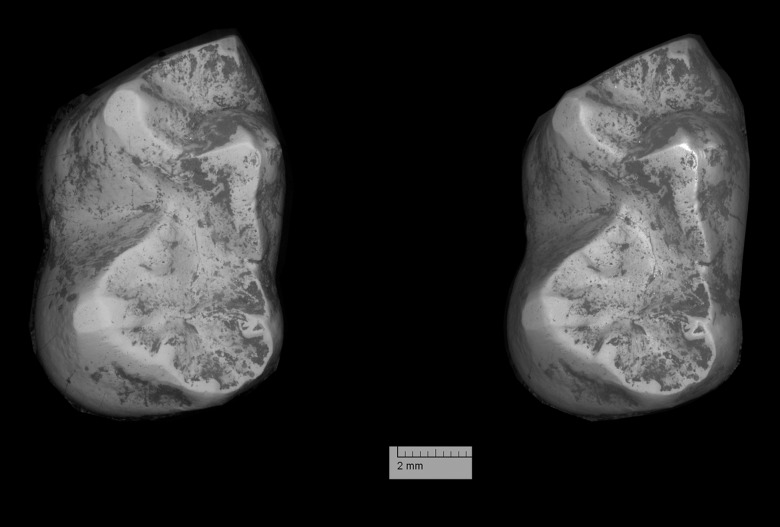
*Ocepeia grandis* n. sp., Thanetian (Phosphate level IIa), Sidi Chennane, Ouled Abdoun Basin, Morocco. MNHN.F PM34, details of the left M_1_ in occlusal view (s.e.m. stereophotograph). Scale bar  = 2 millimeters.

**Figure 19:**
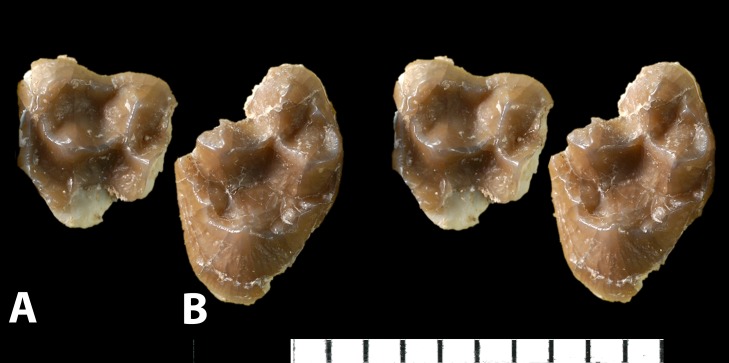
*Ocepeia grandis* n. sp., Thanetian (Phosphate level IIa), Sidi Chennane, Ouled Abdoun Basin, Morocco. MNHN.F PM39, fragments of isolated upper molars: left M^2?^ and left M^1^ (labial part) in occlusal view (stereophotograph). Scale bar:10 mm.

**Figure 20:**
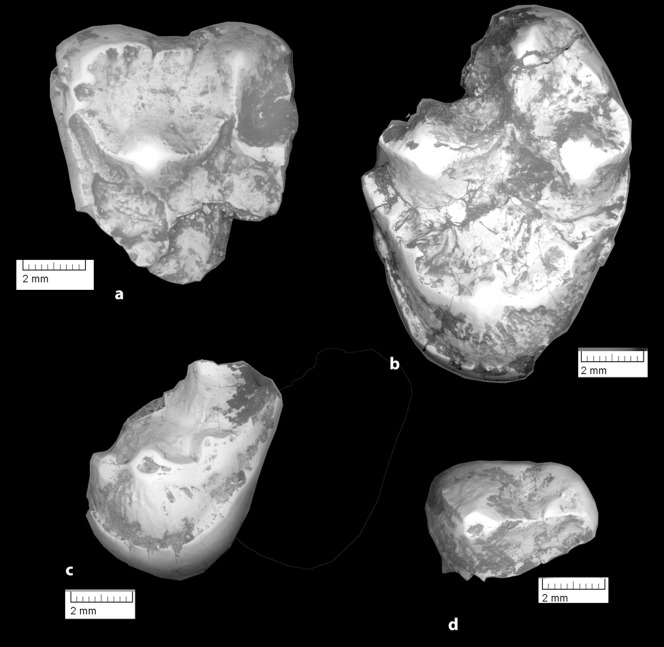
*Ocepeia grandis* n. sp., Thanetian (Phosphate level IIa), Sidi Chennane, Ouled Abdoun Basin, Morocco. MNHN.F PM39, fragments of isolated upper teeth: left M^1^ (labial part, a), subcomplete left M^2?^ (b), right M^1?^ (lingual part, c), and labial part of a left upper premolar in occlusal view (d) (s.e.m. photographs). Scale bar  = 2 millimeters.

**ZooBank life science identifer (LSID) for species**. urn:lsid:zoobank.org:act:1E8AD70F-0465-47BA-B8BA-8D8D2B8C00C9.

**Etymology**. *Grandis* (Latin), in reference to the large size of the species with respect to the type-species *O. daouiensis*.

**Age and occurrence**. This Paleocene species comes from a phosphate level located above that of *O. daouiensis*: Thanetian upper Bone Bed (also known as the coprolites Thanetian Bone Bed) of Bed IIa of the local phosphate series from the Ouled Abdoun Basin (unknown quarries), Morocco [40].

**Holotype**. MNHN.F PM37, fragment of right dentary bearing C1, P3-4, M1-2 (Fig. 14), from an unknown locality of Ouled Abdoun basin.

**Hypodigm**. Holotype MNHN.F PM37; MNHN.F PM34 (Fig. 17), fragment of left dentary with M_1_ (unknown Ouled Abdoun locality); MHNT PAL 2006.0.9 (PM70; Fig. 16), right dentary with P_3–4_, M_1–2_, alveolus for C_1_ and mandibular symphysis (unknown Ouled Abdoun locality, coll. of the Museum of Toulouse); MNHN.F PM39 (Figs 19–20), fragments of isolated upper teeth: subcomplete left M^2?^, left M^1^ (labial part), right M^1?^ (lingual part), and labial part of a left upper premolar (unknown Ouled Abdoun locality).

**Other referred material**. PM66 (Fig. 15), right dentary with M_2–3,_ alveoli for C_1_, P_3–4_, M_1_ (unknown Ouled Abdoun locality; private collection; cast MNHN.F).

**Diagnosis**. Species most closely related to *O. daouiensis*. It differs primarily from *O. daouiensis* in being 150% larger. Other few and discrete morphological features of *Ocepeia grandis* n. sp. that differs from *O. daouiensis* are the conules more developed, the hypoconid and the metacone slightly larger.

### Description and comparisons

This species is known only by part of the cheek dentition and of the lower jaw. The upper dentition is poorly known, only by broken isolated teeth that perfectly occlude with associated lower teeth.

The dental morphology is very similar to *O. daouiensis*. Beside the much larger size, the few and most significant distinct morphological features are mentioned in the diagnosis. The mandibular symphysis was at least partially fused as in *O. daouiensis* (contra [26]). The mandibular corpus seems deeper in *O. grandis* than in *O. daouiensis*. The mental foramina show some variability in their position. A large mental foramina was present between P3 and C1, and another one at least between M1 and P4. MHNT PAL 2006.0.9 shows a small diastema between C1 and P3, but this is also a probable variable feature as in *O. daouiensis*.

By comparison to the entoconid, the molar hypoconid appears proportionally larger in *O. grandis* than in *O. daouiensis*. P4 and P3 are broken in MHNT PAL 2006.0.9, showing a peculiar arrangement of the roots not seen in *O. daouiensis*: The anterior root of these premolars is shifted mesio-labially with respect to the posterior one, and this is more marked in P3. Less inclined wear facet seen on molars might indicate a more lateral component of the chewing stroke in the larger *O. grandis*.

The few known upper molars of *O. grandis* (Figs 19, 20) are very similar to those of *O. daouiensis*. They differ mainly in the larger and more cuspate conules, together with presence of additional small cuspules on the protocristae (Fig. 20), and in the more marked enamel ornamentation with stronger enamel wrinkles. We interpret these features, especially conular development, as a more grinding-rasping functional specialization for harder food than in *O.daouiensis*. The labial fragment of upper premolar of specimen MNHN.F PM39, presents a distal crest and cusp more inflated behind the main labial cusp (paracone) suggesting a more distinct metacone or metastyle than in *O. daouiensis*. It also differs from *O. daouiensis* by the presence of a vertical groove instead of a crest on the anterior flank of the paracone.

Both the evolutionary stage (including in relative size) and the relative age *O. daouiensis* (Selandian) and *O. grandis* (Thanetian) agree with the hypothesis that they belong to a single lineage.

### Dimensions (mm)

Teeth and lower jaw: see Tables 8–10.

**Table 8:** *Ocepeia grandis* n. sp., dimensions of lower dentition (mm): lower teeth.).

	C_1_		P_3_		P^4^		M_1_		M_2_		M_3_	
Specimen	L	W	L	W	L	W	L	W	L	W	L	W
MNHN.F PM34	?	?	?	?	?	?	10.6	7.2	?	?	?	?
MNHN.F PM37	*5.3	*7.4	7.2	5.2	8.5	6.8	11	7.9	11.8	8.7	?	?
MHNT PAL 2006.0.9 (PM70)	?	?	7.4	5.1	8.2	6.2	?	*7.2	9.8	7.9	?	?
PM66	6.2	8	?	?	?	?	?	?	11.2	8.4	>12.6	7.2

L: Length; W: Width.* Estimated measurements.

**Table 9:** *Ocepeia grandis* n. sp., dimensions of dentary (mm): corpus.

	PM66	MHNT PAL 2006.0.9 (PM70)	MNHN.F PM37
Height below M_2_	22	20.2	?
Transverse width below m1	11.1	11.6	11.5

**Table 10:** *Ocepeia grandis* n. sp., dimensions of upper dentition (mm): upper teeth.).

	?P^4^ (or P^3^)		M^1^		M^2^		M^3^	
Specimen	L	W	L	W	L	W	L	W
MNHN.F PM39	7.6	?	?	*9.5	*9.5	13.6	?	?

L: Length; W: Width.* Estimated measurements.

C1-M3 length: PM66: 54.5 mm; MNHN.F PM37: 54 mm; MHNT PAL 2006.0.9 (PM70): 54 mm.

P3-M2 length: MNHN.F PM37: 37.4 mm; MHNT PAL 2006.0.9 (PM70): 33.4 mm.

#### Body mass estimates (Table 11)

Estimates based on the length of M1-3 cannot be calculated because none of the known specimens of *O. grandis* preserves well the molar series. The best body mass estimate of *Ocepeia grandis* based on available material (lower dentition) is slightly more than ten kilograms (Table 11). *O. grandis* was indeed close in size to *Phosphatherium escuilliei*, and much larger than *O. daouiensis*.

**Table 11:** Body mass estimates of *Ocepeia grandis* n. sp. (in grammes; *estimation  =  minimal size).

Measurements base	All ungulates	Selenodonts
Length M_2_	19547	19408
Length M_3_*	**12305**	**10475**
Length M_1–3_	?	?

Predictive allometric equations from [64], [67] for all ungulates, selenodont forms. The best estimates are probably those of the lower range given by M_3_ (although M_3_ is not well preserved in known material of *O. grandis*).

## Phylogenetic Analysis of *OCEPEIA*

The new material of *Ocepeia daouiensis* discovered in the Paleocene of the Ouled Abdoun quarries provides the first data on the morphology of its skull and upper dentition (see reconstruction Figures 11-13). *O. daouiensis* is now the best known mammal from the Paleocene of Africa. Its relationships were poorly known and are here investigated in a new cladistic analysis developed below (see also Text S1).

### 1. Phylogenetic matrix (Text S1, part II)

We investigated the ordinal and supra-ordinal cladistic relationships of *Ocepeia* with the help of the program TNT [68]. In this aim, we used a revised matrix of Gheerbrant et al. [37] and Gheerbrant [69] that was developed for the study of the earliest proboscideans *Phosphatherium* and *Eritherium*, both also known from the Ouled Abdoun phosphate basin. The matrix is extended to include comparisons with basal afrotherians and with other Laurasian “condylarths”.

The new matrix includes 175 characters and 25 taxa that are detailed in Text S1 (Part I). The CT scan study of the skull of *Ocepeia* allowed especially the inclusion of several new characters of the petrosal (middle and inner ear).

With respect to the previously published matrix [37], [69], we expand the taxonomic comparisons with the addition of 7 taxa to our matrix in order to assess the relationships of *Ocepeia* among Laurasian and African placental taxa, e.g. to test relationships with Afrotheria *versus* Laurasiatheria. Our comparisons are extended to the Zhelestidae (petrosal: [59]), *Protungulatum* (petrosal: [52]), *Hyopsodus*, *Todralestes variabilis*, *Potamogale* (Tenrecidae), *Ptolemaia* (*P. lyonsi* and *P. grangeri*: Ptolemaiida), and *Orycteropus afer* (Tubulidentata). We rooted our phylogenetic analysis using the generalized eutherian morphotype (“Eutheria” in the matrix) as outgroup; it is represented by primitive, mostly Cretaceous, eutherians such as leptictids, cimolestids, *Maelestes*, *Asioryctes* and *Acristatherium*.

For the most inclusive taxa such as Eutheria, Perissodactyla, Hyracoidea, Sirenia, Embrithopoda, Zhelestidae, our comparisons are based not on one particular genus or species, but on the most primitive known taxa (see details in Text S1, p.22) that are the best representatives of the hypothetical ancestral morphotype of the clade/group, as presently documented. This is especially important in such autapomorphic taxa as the embrithopods, the tubulidentates, or even the modern hyracoids that have lost many of their supraordinal morphological synapomorphies. Some of these taxa such as the tubulidentates have a so poor fossil record (e.g., no Paleogene fossils) that many of their features cannot be used for comparisons (i.e., autapomorphies and “inapplicable” characters that cannot be homologized, see Table S1). We included the primitive embrithopod *Namatherium* [70] in our comparisons.

The whole matrix includes 3305 states of which about 30% are unknown or “inapplicable” (Table S1). 18 features are uninformative (Table S2) and where excluded from the analysis; they correspond mostly to generalized, and for some, poorly documented traits.

### 2. Cladistic analysis

We developed several analyses, all with the “traditional search” command of TNT, and with both the unweighted and standard implied weighting options. The matrix was analyzed under the following successive conditions:

- All features (175) unordered;

- 152 features ordered;

- 152 features ordered and step matrix for 19 unordered features and 12 other unordered characters coded not reversible;

- Partitioned matrix, separating dental and skull features;

- Constrained analysis to test hypothesis of paenungulate position of *Ocepeia*: matrix with key features weighted (e.g., selenodonty).

### 3. Results and topologies

One remarkable point is that nearly all our analyses of the whole matrix result in the recovery of few most parsimonious trees (MPTs), i.e. a single tree or a well resolved consensus tree. In other words, we obtained few MPTs and all with similar topology. This means that there is some coherent signal among the various characters analyzed, i.e., they do not conflict significantly. Lower topological resolution is obtained with partitioned analysis restricted to skull features which indicates that 1) there are some conflicts among the skull features, 2) dental traits provide the stronger phylogenetic signal in our analysis. However, it should be stressed that some of the partitioned analyses restricted to skull morphology recover the clade Paenungulata and a clade corresponding to Afroinsectiphilia minus Macroscelidea (Text S1, III, Cladograms 7–8), although they do not resolve the position of *Ocepeia*.

**Figure 21:**
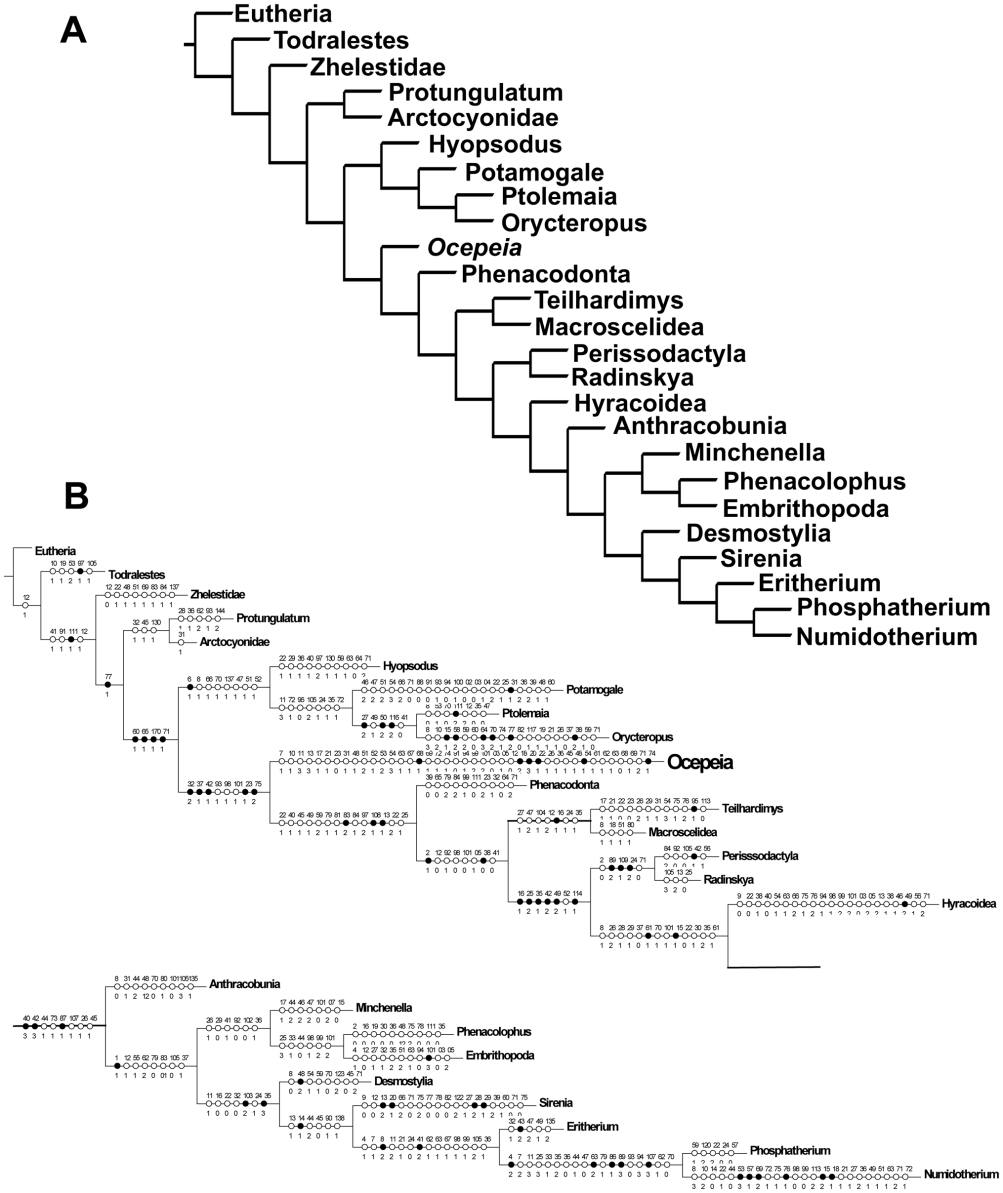
Relationships of *Ocepeia*. Cladogram resulting from parsimony analysis with TNT version 1.1 program of modified matrix of Gheerbrant [68] (Text S1, parts I-II). All features are processed as unordered and unweighted in this tree (see Text S1 for character distribution). Single most parsimonious tree obtained from the “traditional search” command (see Text S1, part III, Cladogram 1). Tree length: 596. Retention index: 53.6. Consistency Index: 40.8. In Figure 21b, the black and open white circles represent respectively strict and homoplasic synapomorphies. This is the shortest tree obtained in all our analyses. The implied weighting analysis does not change the topology. In this analysis *Ocepeia* is by far the most autapomorphic (see text).

The Retention Indices of resulting trees are between 50 and 60, indicating strong homoplasy. For instance, the shortest tree (all features unordered, Fig. 21) includes only 13 non homoplasic characters, i.e. 8% of all analyzed informative features (75 exclusive synapomorphic states, 2% of all sates).

The exclusive sypapomorphies are indicated by an asterisk (*) before the state number.

#### Common nodes and topological patterns

There are several recurrent nodes that are shared among all resulting trees from our different analyses. We discuss these successively from the base to the higher part of the trees.

*Todralestes* always has the basalmost position, as sister-group of all other taxa except the eutherian outgroup. It is indeed excluded from placentals, and should be considered as a stem eutherian as for the Zhelestidae [71]. However, it should be noted that *Todralestes variabilis* remains poorly known (45% of characters unknown in matrix), being documented only by cheek teeth.

Zhelestidae, *Protungulatum*, and Arctocyonidae have a basal position in the trees that is not fixed relative to each other. However, the Zhelestidae is generally the most basal clade.

*Ocepeia* has a basal position in the trees, outside the Paenungulata, “Altungulata” (lophodont ungulates) and “Taxeopoda”. It most frequently belongs to a basal afrotherian clade together with *Potamogale*, *Ptolemaia*, *Orycteropus*, corresponding to the molecular-based clade Afroinsectiphilia minus Macroscelidea, although in some case (e.g., unordered analysis) it is a more derived branch (sister group of all other taxa). Relationships with Afroinsectiphilia afrotherians result from the analysis of the whole matrix and from partitioned analysis restricted to dental features, which indicates that the phylogenetic signal is mostly dental. Bremer support for this node (Afroinsectiphilia and *Ocepeia*) is rather low. Within Afroinsectiphilia, *Ocepeia* joins preferentially *Potamogale* (e.g., 148-1, postorbital process reduced), and *Ptolemaia* groups with *Orycteropus* in many of our analyses (ordered, unordered, and step matrix; Text S1, part III). The clade (*Ptolemaia*, *Orycteropus*), that is well supported by 3 exclusive synapomorphies (*27-2, *50-2, *116-2; ordered analysis), agrees with the conclusions of Simons & Gingerich [72] and Seiffert [73].

In the higher part of the MPTs obtained in this work, the main clades are stable and robust; in nearly all trees, we recover the following clades: (Phenacodonta (*Teilhardimys*, Macroscelidea, (Perissodactyla, Paenungulata))). This node is supported by 3 exclusive synapomorphies of which the most noticeable are the upper molars with lingual root enlarged and bearing a sulcus, and with developed interloph. Upper molar with incipient lophodonty (89-1) appears at this node or at the next node (*Teilhardimys*, Macroscelidea, (Perissodactyla, Paenungulata)).

The relative position of *Teilhardimys* and Macroscelidea to each other (i.e., same clade or not) changes in several trees. In the partitioned analysis restricted to skull features (Text S1, part III, Cladograms 7-8), the Macroscelidea groups with basal afrotherians, as in the molecular-based Afroinsectiphilia hypothesis.

Following Gheerbrant et al. [37] and Gheerbrant [69], we recover the sister-group relationships of Perissodactyla (usually allied to *Radinskya*) and Paenungulata, that corresponds to the taxon “Altungulata”. This is a very robust node in our analysis (ordered analysis: 5 exclusive synapomorphies: *25-2, *35-1, *42-2, *49-2; 4 homoplastic synapomorphies: 11-0, 16-1, 52-1, 90-2). Consequently, it would refute the monophyly of the clades Afrotheria and Laurasiatheria. It is supported especially by the lophodonty (*25-2, *35-1, *42-2) and the enlarged M_3_ (*49-2). However, the latter feature implies unlikely reversal in *Eritherium*, and the lophodonty is most likely convergent in Perissodactyla and Paenungulata, as indicated by detailed structural traits (see below).We suggest, following Gheerbrant [69], that our cladistic analysis remains unable to detect convergence of lophodonty in Laurasian and African “ungulates” because of fossil gaps, i.e. because of unknown early structural steps in its evolution. We lack data especially on the dental ancestral morphotype of perissodactyls, but also on some questionable paenungulates such as *Phenacolophus*, *Minchenella*, and anthracobunids. Phenacodonts are plesiomorphic with a low stem position and do not help to solve this question in the analysis. Only the partitioned analysis restricted to cranial features (ordered analysis; Text S1, part III, Cladograms 7-8) does not recover sister-group relationships of Perissodactyla and Paenungulata. This confirms that the signal for “Altungulata” here is mostly dental (e.g., lophodonty).

The node Paenungulata occurs in all our trees with identical taxonomic content. However, 1) Bremer supports are generally low, except in unordered analysis (Fig. 21; Text S1, III, Cladogram 1), 2) internal (inter-ordinal) topology varies strongly. In the ordered analysis (Fig. 21), the clade is supported by three exclusive synapomorphies (*61-1: coronoid foramen present; *115-1: M^3^ with one hypocone root; *135-2: orbit above P4-M1) and 9 homoplastic synapomorphies of which the best ones (RI>75) are lower molars with loph transverse (26-2) and with large lingual cusps (28-1), and amastoidy (161-1). The presence of a coronoid foramen, unknown in *Ocepeia*, was recently evidenced in macroscelideans [74]. The amastoidy is known in *Ocepeia*, as well as the selenodont ectoloph (101-2).

The clade Tethytheria is generally recovered at least for the crown groups Sirenia and Proboscidea, but the position of stem taxa such as Embrithopoda, Anthracobunia, Desmostylia is variable, and the Bremer supports are low. Tethytheria is not recovered only in partitioned analysis with step matrix restricted to dental features (Text S1, part III, Cladogram 11), in which Proboscidea joins the Hyracoidea.

The clade Proboscidea is one of the most stable in all our analyses. It is supported by a strong Bremer Index.

**Figure 22:**
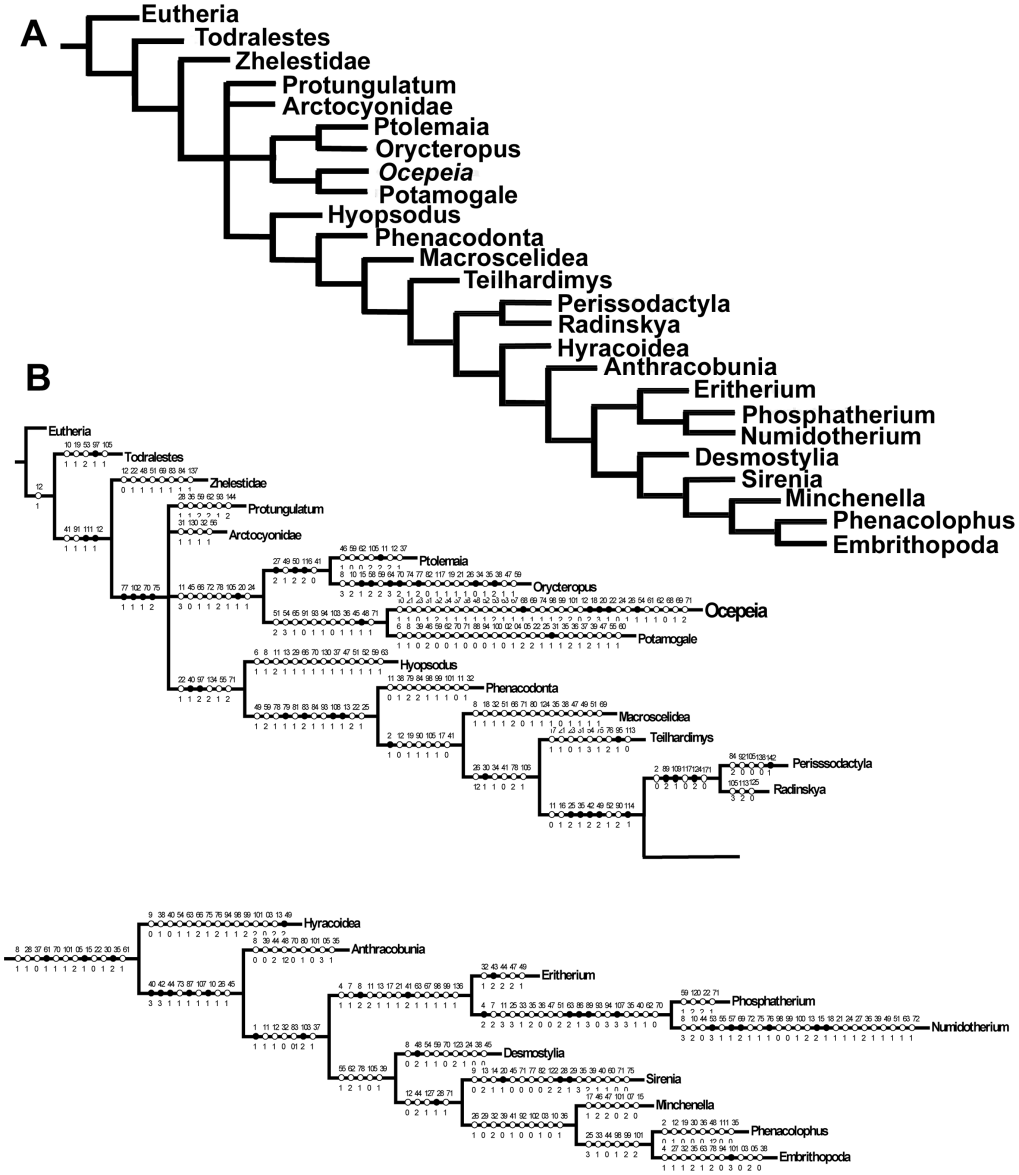
Relationships of *Ocepeia*. Cladogram resulting from parsimony analysis with TNT version 1.1 program of modified matrix of Gheerbrant [68] (Text S1, parts I-II). 152 features are processed as ordered in contrast to Figure 21 (Text S1, part I). Strict consensus of 2 trees resulting from the “traditional search” command (Text S1, part III, Cladogram 3). Tree lengths: 665. Retention index: 54.6. Consistency Index: 37. In Figure 22b, the black and open white circles represent respectively strict and homoplasic synapomorphies. This is **our reference topology** for our discussion of the relationships of *Ocepeia*. In this analysis *Ocepeia* is also strongly autapomorphic.

Embrithopoda. A clade (*Minchenella* (*Phenacolophus* (Embrithopoda))) is very stable (several analyses, see Text S1). It is supported by 10 homoplastic features in the ordered analysis (Fig. 22). However, it should be noted that a recent analysis of dental enamel microstructure argues against close relationships of *Phenacolophus* and Embrithopoda [77]. We concur, and suggest that the cladistic relationships of *Phenacolophus* recovered here results from unresolved significant dental convergences, in the same way as between perissodactyls and paenungulates. This is probably related to our poor knowledge of *Phenacolophus* (59% of its characters unknown in matrix) and *Minchenella* (50% of its characters unknown in matrix) which are known only by dental specimens and are among the less known analysed taxa. Actually, it should be emphasized that even the described dental material of *Phenacolophus* [78] is very poorly preserved – a point which is widely underestimated in current phylogenetic discussions; for instance the structural dental homology remains widely to check. Embrithopods are usually sister-group of Sirenia as recovered by Seiffert [73], except in partitioned analysis (ordered) restricted to dental traits in which they are stem tethytheres (Text S1, part III, Cladograms 5-6).

#### The different topologies obtained

**Figure 23:**
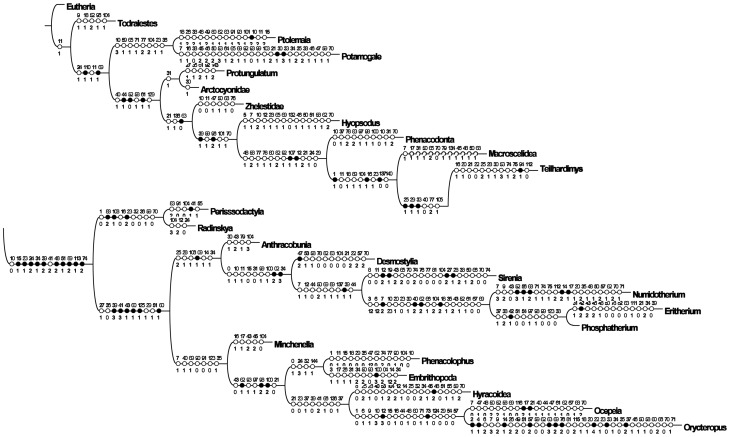
Relationships of *Ocepeia*. Cladogram resulting from parsimony analysis with TNT version 1.1 program of modified matrix of Gheerbrant [68] (Text S1, parts I-II). This tree results from an analysis in which *Ocepeia* is constrained to cluster with paenungulates by increasing the weight of characters shared with paenungulates (Tables 6, 7, 12), i.e., characters 98, 99, 101 that are directly related to the selenodonty. Consensus of two trees (Text S1, part III, Cladogram 13). This tree is well resolved but significantly longer than trees resulting from other analyses (Figs. 21, 22). Tree length: 767; retention index: 53.2; consistency index: 37.5. The black and open white circles represent respectively strict and homoplasic synapomorphies; see Text S1, part III, for description of characters. In this tree *Ocepeia* is noticeably less autapomophic. See text for further comments.

- Unordered analysis (Fig. 21; Text S1, part III, Cladograms 1-2). The analysis of the matrix with all features unordered provides a single resolved tree, which is the shortest tree (length 596, RI 53.6, CI 40.8) obtained in all our analyses. It differs from most other analyses in the interchanged position of *Hyopsodus* and *Ocepeia*: the former joins the primitive afrotherian clade (afroinsectiphilians), and the latter is more derived as sister group of all other taxa that are characterized by incipient lophodonty. The implied weighting analysis does not change the topology. In this analysis, *Ocepeia* is by far the most autapomorphic with 6 exclusive autapomorphies and 35 homoplastic traits.
- Ordered analysis (Fig. 22; Text S1, part III, Cladogram 3). The analysis of the matrix with 152 ordered features provides only 2 trees which differ in the relative position of *Protungulatum* and Arctocyonidae. The consensus tree (Length 665, RI 54.6, CI 37) illustrated Figure 22 is **our reference topology** for our discussion of the relationships of *Ocepeia*. *Ocepeia* is the sister group of *Potamogale*, and both are sister group of the clade (*Ptolemaia*, *Orycteropus*), corresponding to the clade Afroinsectiphilia (minus Macroscelidea). Analysis with standard “implied weighting” command provides a single tree that fixes relative position of *Protungulatum* and Arctocyonidae (Text S1, part III, Cladogram 4). In this analysis *Ocepeia* is strongly autapomorphic, as it is in the unordered analysis (36 homoplastic states, 5 exclusive autapomorphies).
- The partitioned ordered analysis (Text S1, part III, Cladogram 5) restricted to dental features recovers similar topology than in the unpartitioned analysis (Fig. 22; e.g. *Ocepeia*  =  basal afrotherian) except in the derived position of *Orycteropus* that clusters with Proboscidea, probably as the result of the optimization of many features (50% of unknown data in *Orycteropus*; see Table S1). The partitioned ordered analysis restricted to skull features and to taxa only documented by skull features (Text S1, III, Cladogram 8) recovers two main clades: basal afrotherians (*Potamogale*, *Orycteropus* and Macroscelidea, *i.e.* clade Afroinsectiphilia) and Paenungulates (but including Zhelestidae). The position of *Ocepeia* is surprisingly not resolved in the partitioned ordered analysis restricted to the skull. Similar results are obtained with the TNT “implied weighting” command.
- Ordered analysis with step matrix (Table S4 and Text S1, part III, Cladograms 9-12). In this analysis we ordered the state transformation according to the most likely successive structural steps, and we excluded unlikely reversals (e.g., reappearance of teeth; see Table S3). The analysis recovers 8 trees of which the consensus agrees (Text S1, part III, Cladogram 9) with the ordered analysis (Fig. 22; Text S1, part III, Cladogram 3). Length, retention and consistency indices of trees from step matrix analysis (Text S1, part III, Cladogram 9) are very close to the ordered analysis. In this analysis, Proboscidea are supported by a strong Bremer Index, and *Ocepeia* clusters with basal afrotherians. “Implied weighting” command provides a single tree (Text S1, part III, Cladogram 10) which differs in the interchanged position of *Hyopsodus* and *Ocepeia*, the latter which is excluded from basal afrotherians. In this analysis *Ocepeia* is much less autapomorphic, with 28 autapomophic traits.
- The partitioned analysis including ordered features and step matrix (Text S1, part III, Cladograms 11-12) shows that dental features yield the most significant phylogenetic signal: 1) dental features provide one single tree very similar to the ordered analysis, except for the position of *Orycteropus* that surprisingly joins Proboscidea (as in analysis without step matrix); 2) skull features provide an unresolved basal polytomy (Text S1, part III, Cladogram 12).
- Constrained analysis for paenungulates relationships of *Ocepeia* (Fig. 23; Text S1, part III, Cladogram 13). We constrained the analysis to cluster *Ocepeia* with Paenungulata by increasing the weight of features shared with paenungulates (Tables 6, 7, 12). The most important shared characters with paenungulates that impacts topology in the cladistic analysis are characters related to the **selenodont pattern** of upper molars (ectoloph dilambdodont and joined to a developed mesostyle: characters 98, 99, 101). Other shared features (see Table 12), such as the occurrence of an entolophid (34-1), do not support cladistic relationships with paenungulates at similar weight cost. Constrained analysis with weighted upper molar selenodonty results in only two trees, the consensus of which is well resolved (Fig. 23; Text S1, part III, Cladogram 13) and significantly longer than in other (unweighted) analyses (RI = 53.2, CI = 37.5, Length = 767). Bremer supports in resulting trees are higher than in other analyses, including for nodes among Paenungulata (e.g., Proboscidea, Tethytheria). In this topology the node Paenungulata (node 23) is supported by 5 exclusive synapomorphies (*40-3, *42-3, *61-1, *126-1, *161-1 and 4 homoplastic features (28-1, 37-0, 130-1, 152-1). However, the node is paradoxically not supported by features related to the selenodonty, following our hypothesis; these features support in fact the more internal paeungulate node 31 ((*Phenacolophus*, Embrithopoda) (Hyracoidea (*Ocepeia*, *Orycteropus*))). In these trees *Ocepeia* is noticeably less autapomophic (16 homoplasies; 2 exclusive states, including: 118-2, extensive pneumatization; 122-1, palatine posterior to M^1^). Within Paenungulata, *Ocepeia* has a nested derived position rather than a stem position; it clusters with *Orycteropus*, both as sister group of Hyracoidea (with good Bremer support). We reject this hypothesis of the derived position of *Ocepeia* within Paenungulata because it admits unlikely reversal of the hypocone that is typically absent in *Ocepeia*. This topology implies other noticeable reversals in *Ocepeia*. A stem position of *Ocepeia* to Paenungulata would be actually more parsimonious for several features at the node of crown Paenungulata (e.g. coronoid foramen, frontal-maxilla contact, lower molars without entoconulid, and with hypoconulid low and distal).
- Finally, we analyzed the position of *Ocepeia* in the exclusive context of the afrotherian relationships (i.e., relationships within the monophyletic clade Afrotheria); in this way we excluded from the analysis putative laurasiatherian ungulates such as Perissodactyla, *Radinskya*, Phenacodonta, and even *Hyopsodus*. In all obtained trees, *Ocepeia* keeps the same outgroup position with respect to Macroscelidea and Paenungulata, in the same clade with *Potamogale* and *Orycteropus* (Afroinsectiphilia). This is especially related to the absence of hypocone and lophodonty in *Ocepeia*.

**Table 12:** Cladistic interpretation of shared characters of *Ocepeia* with ungulates and paenungulates (ordered analysis, Fig. 21).

Characters	Description	Convergence with	Remarks
28–1 (RI = 88)	Lower molars with lingual cusps larger than labial cusps	*Protungulatum*, **Paenungulata**	
34–1 (RI = 88)	Hypolophid present	(Perissodactyla, *Teilhardimys*, **Paenungulata**)	
63–1 (RI = 50)	Anterior coronoid (retromolar) fossa present	Some Paenungulata: Hyracoidea, Proboscidea, Embrithopoda	Not Sirenia, Desmostylia, *Minchenella*, *Phenacolophus*, Anthracobunia
98–1 (RI = 16)	Mesostyle present	Phenacodonta, some Paenungulata: Hyracoidea, Proboscidea, Desmostylia, Embrithopoda	Not Sirenia and advanced Proboscidea (reversals)
101–1 and 101–2 (RI = 33)	Centrocrista dilambdodont and linked to the mesostyle	Phenacodonta, Paenungulata	Not *Minchenella*; centrocrista secondarily lost in Sirenia, Desmostylia; convergence with Hyracoidea and Embrithopoda at state 2; convergence with Phenacodonta and Paenungulata at state 1
161–1 (RI = 83)	Periotic amastoidy	**Paenungulata**	
126–1 (RI = 71)	Nasal cavity wide and high	*Orycteropus*, Tethytheria	Not Hyracoidea (primitive state?)
145–1 (RI = 60)	Zygomatic arch high dorso-ventrally with ventral process at maxillary suture	Tethytheria	Not Hyracoidea and Desmostylia (primitive state?)
136–1(RI = 50)	Orbit bordered ventrally partly by a short process of the maxillary which extends between the lacrimal and jugal	Some Paenungulata: Hyracoidea, Proboscidea, Embrithopoda	Not Sirenia, Desmostylia, Anthracobunia
7–1 (RI = 50)	I_3_ vestigial	Proboscidea (*Eritherium*)	I_3_ becoming lost in proboscideans
67–1 (RI = 0)	I^2^ enlarged	Proboscidea	
162–1 (RI = 50)	Pars mastoidea larger than pars cochlearis	Proboscidea	
25–1 (RI = 92), 88–1 (RI = 50)	M_1–3_ bunodont with low trigonid		“Ungulate” plesiomorphy?
62–1 (RI = 40)	Angular process broad and rounded posteriorly projecting		“Ungulate” plesiomorphy?
111–1 (RI = 33)	M^1^<M^2^		“Ungulate” plesiomorphy?

RI: Retention Index.

### 4. Discussion: Relationships of *Ocepeia*, morphological support and significance

Our cladistic analysis confirms that Ocepeia daouiensis is plesiomorphic among placentals, as was indicated in previous studies of more limited material [69]. This is most noticeably shown by the skull construction that is eutherian-like in many respects (Table 5), and this is confirmed in the analysis restricted to cranial features (Text S1, part III, Cladograms 7-8). Some characters of the dentition, such as the absence of a hypocone, the presence of a paraconid, and the large canine, are also remarkable plesiomorphies.

As a matter of fact, the several and noticeable ungulate-like features of *Ocepeia daouiensis* are not optimized in the phylogenetic analysis that excludes it from African and Laurasian “ungulates” (including primitive taxa), but instead relates it to basal afrotherians.

#### Relationships with Placentalia

Whereas *Todralestes* is a stem eutherian, several features indicate unequivocal relationships of *Ocepeia* with crown placentals. This is especially true of the inner ear morphology (e.g., cochlear canal with two full turns), as identified from the CT scan study.

#### Relationships with Afrotheria

The most striking result of the cladistic analysis of *Ocepeia* is its relationships with Afrotheria, and especially its basal position within Afrotheria. The primitive morphology of the skull of *Ocepeia* agrees with its basal position in Afrotheria, and also with the basal position of Afrotheria among placentals.

However, the relationship with an exclusive clade of basal afrotherians such as *Potamogale*, *Ptolemaia* and *Orycteropus*, i.e. the clade Afroinsectiphilia minus Macroscelidea, remains poorly supported morphologically. The single identified exclusive synapomorphy with afroinsectiphilian implies an unlikely character transformation in *Ocepeia* (120-1>3: rostrum very narrow to very wide). Six homoplastic synapomorphies (ordered analysis) also support the node, most with low RI (40-60), and of which the best is the loss of P^1^_1_ (11-3 RI = 41; 72-1 RI = 75). Within basal afrotherians, the reduced postorbital process (148-1) is interpreted as an unambiguous synapomorphy of *Ocepeia* and *Potamogale*.

A peculiar relationship of *Ocepeia* with basal afrotherian taxa such as *Potamogale* is questionable in regard to its many remarkable ungulate-like features, for instance. We interpret the basal afrotherian placement of *Ocepeia* in our trees as a rather more general phylogenetic signal for its afrotherian relationships. However, our analysis does not recognize a monophyletic clade Afrotheria and, hence, any exclusive afrotherian synapomorphy in the Moroccan genus. This is related to the more general problem that the monophyly of the supercohort Afrotheria remains poorly characterized morphologically on the basis of available fossil and neontological morphological data (but see [73], [76], [79]). In this regard, *Ocepeia* should help to characterize the ancestral morphotype of the afrotherians.

The sister- or stem-group position of *Ocepeia* to the whole Afrotheria cannot be indeed rejected, especially if we agree with the hypothesis of an ungulate-like ancestral afrotherian morphotype, instead of an insectivore-like ancestral afrotherian morphotype, as raised by Seiffert [73], [75], [76]. However, this does not fit well with 1) the early diversity of the insectivorous-grade mammals from the Paleocene of Morocco [32], [33], [34], and 2) several striking paenungulate-like traits of *Ocepeia,* such as the selenodonty (see below) that remain unknown in extant and fossil insectivoran-like afrotherians (Afroinsectiphilia). Moreover, the synapomorphies that support in our cladistic analysis a relationship of *Ocepeia* to insectivoran-like afrotherians (Afroinsectiphilia) are weak (see above).

#### Relationships with Paenungulata

Within Afrotheria, our cladistic analysis places *Ocepeia* outside of the Paenungulata. Even the constrained analysis that clusters *Ocepeia* with paenungulates results in a hypothesis of nested paenungulate position that is refuted by unlikely implied characters transformations such as a secondary loss of the hypocone.

Whatever the cladistic results, the new material of *Ocepeia* described here evidences a combination of primitive eutherian features, placental traits and ungulate-like traits. The ungulate-grade traits include proto-ungulate-like and paenungulate-like features that are summarized in Tables 6, 7 and 12. These traits have a noticeable heterogeneous distribution in our trees. The generalized ungulate-grade traits of *Ocepeia* are the low and bunodont teeth (e.g., 25-1, 88-1), and the posteriorly projecting broad angular process of the dentary (62-1). The most remarkable paenungulate-like features of *Ocepeia* are dental traits such as especially the presence of an incipient entolophid (34-1, see also [26]), and the ectololoph selenodont (W-shaped centrocrista, 101-2) and linked to an inflated mesostyle (98-1, 99-2 or 99-1), and lower molars with large inflated lingual cusps (28-1). The ectoloph is secondarily lost within paenungulates (e.g., Proboscidea), and the entolophid is known in perissodactyls. Some noticeable paenungulate features of the skull such as the periotic amastoidy (161-1), the developed zygomatic process of the maxilla, and lower molars with enlarged lingual cusps are known in *Ocepeia*. Amastoidy in the only trait restricted to paenungulates in our analysis. Several other traits of *Ocepeia* are known in paenungulates (Table 12), but not in all of them for several reasons such as reversals, convergences, or poor knowledge. The most remarkable ones are the high (deep) zygomatic arch with ventral process below the orbit (145-1), the short process of the maxilla in the orbit rim (136-1), presence of a coronoid fossa (63-1), and wide nasal cavity (126-1). Some paenungulate-like traits of *Ocepeia* seem even restricted to proboscideans (Table 12: 7-1, vestigial I_3_; 67-1, enlarged I^2^; 162-1, large pars mastoidea). The ancestral condition of several of these traits remains poorly known in several paenungulate orders such as hyracoids (e.g., features 126, 145), sirenians (63, 98, 99, 101, 136) and embrithopods (63, 136).

Table 12 summarizes the interpretations of the paenungulate-like traits of *Ocepeia* in the trees (ordered analysis). The shortest trees (unconstrained parsimonious analyses, Figs. 21-22) admit noticeable parallelisms of *Ocepeia* and Paenungulata in their ungulate-like traits (Table 12), which instead are autapomorphic in both taxa. Resemblances with Proboscidea are also interpreted as convergences in the cladistic analysis. As a result *Ocepeia* is strongly autapomorphic in our trees (31 autapomorphies in ordered analysis, 41 in unordered analysis).

In fact, the shared traits of *Ocepeia* and Paenungulata conflict with the shared lophodonty of Paenungulata and Perissodactyla which is interpreted in our parsimony analyses as a synapomorphy for a questionable clade “Altungulata”, and which challenges the monophyly of Afrotheria in our trees. The morphotypic bunodont incipiently lophodont pattern of primitive proboscideans and paenungulates (89-1, also shared by *Teilhardimys* and primitive macroscelideans) is well distinct from the advanced lophodont pattern (89-2) of primitive perissodactyls, supporting the probable convergence of Laurasian and African ungulate-like mammals [69]. Together with remarkable shared features of *Ocepeia* and paenungulates such as the selenodont ectoloph, that is unknown in primitive perissodactyls, this challenges such a clade “Altungulata”, and agrees with the alternative hypothesis of the relationships of *Ocepeia* and Paenungulata.

In this hypothesis, the proto-ungulate features of *Ocepeia* best fit with its primitive stem position with respect to Paenungulata. The most noticeable plesiomorphic features that exclude *Ocepeia* from the crown group of extant paenungulate orders are the presence of paraconid, the absence of hypocone and related absence of bilophodonty, and the large canines. Crown paenungulates also depart by the M^3^ with two lingual roots (115-1), the molar trigonid compressed with reduced paracristid (29-1), presence of coronoid foramen (61-1), orbit more anterior (above P^4^-M^1^, 135-2) and possibly relatedly the tuber maxillae more posterior to orbit (143-1), and a more developed posttympanic process (158-1). Such hypothesis of a stem paenungulate position of *Ocepeia* would actually be more parsimonious for several features at the nodes of stem and crown Paenungulata.

In our view, the **selenodont morphology of the molar ectoloph is a structural landmark of the monophyly of the paenungulates** including stem taxa such as *Ocepeia*, i.e. as a key feature of their ancestral morphotype. The incipient entolophid is another, but more derived, significant shared derived trait. Together with the relationships of *Ocepeia* with basal afrotherians (afroinsectiphilians) obtained in the parsimony study, this would make it the first known fossil link between insectivore-like and ungulate-like afrotherians.

Recent Eocene discoveries evidenced intriguing paenungulate dental and postcranial affinities of the European louisinid *Teilhardimys* and some primitive afrotherians such as early macroscelideans (e.g., [79]). In contrast to *Ocepeia*, these taxa share with paenungulates especially a large hypocone and the primitive bunodont-bilophodont pattern. However, they lack the selenodont ectoloph linked to a well-developed mesostyle and other paenungulate features seen in *Ocepeia* such as the incipient entolophid, implying unsuspected noticeable convergent evolution of ungulate-like features within Afrotheria (see Table 12).

#### The autapomorphic and endemic nature of *Ocepeia*

Our cladistic analysis emphasizes the autapomorphic nature of *Ocepeia*, that is here distinguished at least at familial level in the **new family** **Ocepeiidae** (see diagnosis). The ordered analysis (Fig. 22) records 31 autapomorphies; 5 are exclusive autapomorphies (68-1, 118-2, 120-3, 122-1, 154-1) and others are mostly homoplastic convergences with paenungulates (see above and Table 12). In this topology, *Ocepeia* is by far as the most autapomorphic taxon compared in this work. The constrained analysis however shows that *Ocepeia* is much less autapomorphic when it clusters with paenungulates; in this topology *Ocepeia* has only 18 autapomorphies (including 2 exclusive states), and *Orycteropus* is consistently much more autapomorphic.

The most remarkable specialized features of *Ocepeia* are the anthropoid-like traits of the skull face and dentition (Tables 6, 7), such as the rostrum short and robust and the shortened anterior dentition. Other important specialized features of *Ocepeia* are the skull with elongated meso- and postero-cranial region as illustrated especially by the very long parietal and related short frontal. The noticeable pneumatization of the skull bones (a feature that evolves parallely in large proboscideans) is also remarkable; its adaptive significance remains unknown (structural pattern specialized for enhanced sound transmitting?).

These autapomorphic traits are evidences of the strong endemic nature of *Ocepeia*, and the long evolutionary history of its lineage in Africa.

## Conclusions

The new material reported here from the Paleocene (Selandian) of the Ouled Abdoun Basin considerably enhances our knowledge of early afrotherians and African condylarth-like mammals. It enlightens the skull morphology of *Ocepeia daouiensis* that is reconstructed (Figs. 11–13) and studied here. *O. daouiensis* is today the best known Paleocene mammal from Africa. Another new species of the same genus, *O. grandis* n. sp., is described from the same basin in a higher Paleocene level of Thanetian age. It differs mostly by its larger size, and it is a probable direct descendant of *O. daouiensis*. *O. daouiensis* and *O. grandis* belong to the new placental family Ocepeiidae, that is only known from the Paleocene of the Ouled Abdoun Basin (Morocco) at that time.

The cladistic analysis of *Ocepeia* supports its placental and afrotherian relationship. However, it does not fix its ordinal and supra-ordinal position within Afrotheria. The most parsimonious trees (MPTs) support a relationship of *Ocepeia* with basal insectivore-like afrotherians (i.e., afroinsectiphilians), in contrast to its many paenungulate-like traits. These most parsimonious trees involve 1) noticeable parallelisms of *Ocepeia* and Paenungulata in their ungulate-like traits, 2) a mostly dental relationships of Paenungulata and laurasiatherian ungulates such as Perissodactyla. The Paenungulata - Perissodactyla sister group relationship ( = ”Altungulata”) recovered in our most parsimonious trees is considered here as the result of a convergence obscured by fossil gaps that make relationships of *Ocepeia* uncertain within Afrotheria. The shared derived features of *Ocepeia* and paenungulates are not optimized as exclusive synapomorphies because they conflict with undetected convergences, in our most parsimonious trees, of Paenungulata and Perissodactyla such as the bilophodonty. A recent study of the enamel microstructure of *Phenacolophus* [77] confirms for instance the convergence of this bilophodont Laurasian genus with paenungulates. These convergences of Laurasian and African ungulates also conflict with a monophyletic clade Afrotheria in our most parsimonious trees.

Within Afrotheria, the remarkable paenungulate-like features of *Ocepeia* are much more significant than the few and weak synapomorphies recovered in our MPTs with afroinsectiphilians. The selenodont pattern is one of the most remarkable shared derived features of *Ocepeia* and paenungulates. However, the skull and dentition of *Ocepeia* show a much more primitive construction with respect to extant paenungulate orders. This excludes unambiguously *Ocepeia* from the crown clade Paenungulata that departs for instance in the bilophodont molar pattern. Such a combination of primitive and derived features in *Ocepeia* actually best fits with its stem position to Paenungulata. The sister-group relationship of *Ocepeia* to Paenungulata needs however to be further and more formally established, hence our referral with a question mark.

*Ocepeia* actually shows a remarkable mosaic of autapomorphic, ungulate-like and plesiomorphic features. In particular, the combination of plesiomorphic eutherian-like and afrotherian-like skull features, and of proto-ungulate and paenungulate-like dental traits, makes *Ocepeia* the first and best known “transitional fossil” between insectivore-like and ungulate-like afrotherians [80], [81].

The family Ocepeiidae is representative of a specialized endemic African lineage characterized by unexpected anthropoid-like features of the dentition and muzzle, and by other skull traits such as elongated meso- and postero-cranial region (e.g., long parietal) and short frontal, and remarkably pneumatized bones (e.g., occipital). It indicates an old history in Africa, since the very beginning of the Tertiary or earlier. This supports the old endemic evolution of Afrotheria and placentals in Africa, in contrast to hypotheses supposing their origin from Paleogene Laurasian stem “condylarths” such as louisinids and apheliscids [82].

## Supporting Information

Figure S1 ***Ocepeia daouiensis*** **, skull MNHN.F PM45, 3D CT scan model of the original specimen (unretouched).**
(PDF)

Figure S2 ***Ocepeia daouiensis*** **, skull MNHN.F PM45, 3D CT scan model reconstruction.** A. dorsal view. B.ventral view. C.lateral view. D. posterior view.
(TIF)

Table S1 **Matrix of** ***Ocepeia*** **: Data completeness.** We distinguish here absence of data (character marked by a question mark) from unapplicable characters (“-”  =  gaps in TNT); unapplicable characters correspond to features that cannot be homologized. Undocumented and unapplicable features are however processed similarly in TNT. The larger number of unapplicable traits occurs in *Orycteropus* that is the most specialized taxon compared in our cladistic analyses.
(DOC)

Table S2 **Matrix of** ***Ocepeia*** **: 18 uninformative characters (inactived in the analysis).**
(DOC)

Table S3 **Matrix of** ***Ocepeia*** **: Irreversible characters.**
(DOC)

Table S4 **Matrix of** ***Ocepeia*** **: Step matrices for 19 characters, all non additive; other transformations for these characters are coded unlikely with a cost of 10 steps instead of one step.**
(DOC)

Text S1 **Phylogenetic analysis of** ***Ocepeia daouiensis*** **.** Part I: Studied characters of *Ocepeia daouiensis*. Part II: Characters matrix for analysis of *Ocepeia daouiensis*, characters summary list, taxa analyzed. Part III: TNT analysis, method, cladograms, and diagnose of nodes. Part IV: References.
(PDF)

Video S1 ***Ocepeia daouiensis*** **, skull MNHN.F PM45, 3D CT scan model of the original specimen (unretouched).**
(AVI)

Video S2 ***Ocepeia daouiensis*** **, skull MNHN.F PM45, 3D CT scan model of the left petrosal.** Red: fenestra vestibuli; blue: f. cochleae; green: cochleae canaliculus.
(AVI)
