# Supplementary figures and images for: Ocepeia (Middle Paleocene of Morocco): The Oldest Skull of an Afrotherian Mammal
Source: PLoS One. 2014 Feb 26;9(2):e89739. doi: 10.1371/journal.pone.0089739 (PMC3935939; doi:10.1371/journal.pone.0089739)

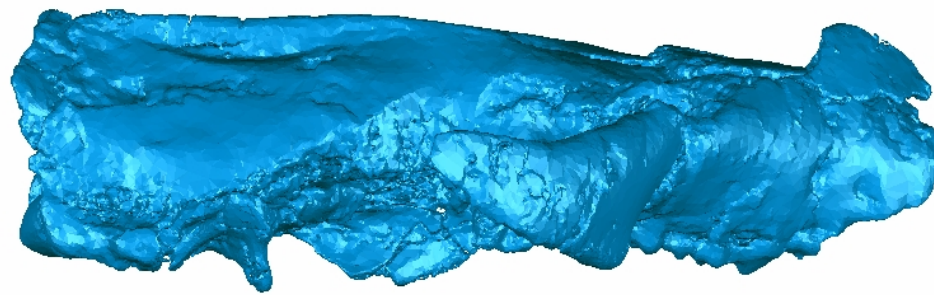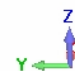

Click on the image to activate the 3D Model.

Supplement: Figure S1 — Ocepeia daouiensis , skull MNHN.F PM45, 3D CT scan model of the original specimen (unretouched). (PDF) [file pone.0089739.s001.pdf]

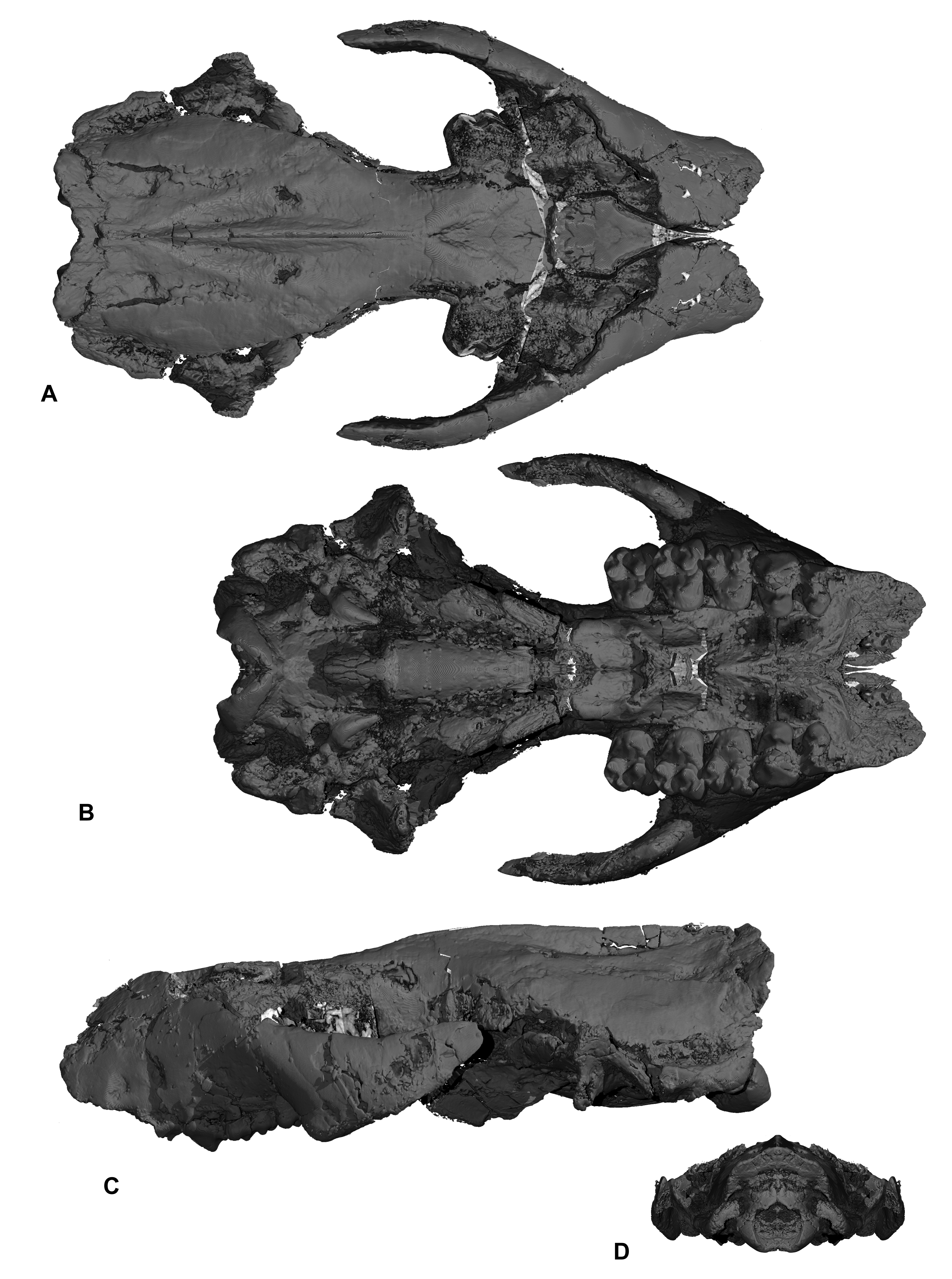

Supplement: Figure S2 — Ocepeia daouiensis , skull MNHN.F PM45, 3D CT scan model reconstruction. A. dorsal view. B.ventral view. C.lateral view. D. posterior view. (TIF) [file pone.0089739.s002.tif]
