# Supplementary material for: Ocepeia (Middle Paleocene of Morocco): The Oldest Skull of an Afrotherian Mammal
Source: PLoS One. 2014 Feb 26;9(2):e89739. doi: 10.1371/journal.pone.0089739 (PMC3935939; doi:10.1371/journal.pone.0089739)
Supplement: Table S1 — Matrix of Ocepeia : Data completeness. We distinguish here absence of data (character marked by a question mark) from unapplicable characters (“-” = gaps in TNT); unapplicable characters correspond to features that cannot be homologized. Undocumented and unapplicable features are however processed similarly in TNT. The larger number of unapplicable traits occurs in Orycteropus that is the most specialized taxon compared in our cladistic analyses. (DOC) [file pone.0089739.s003.doc]

**Table S1. Matrix of *Ocepeia*: Data completeness.**  We distinguish here absence of data (character marked by a question mark) from unapplicable characters (“-“ = gaps in TNT); unapplicable characters correspond to features that cannot be homologized. Undocumented and unapplicable features are however processed similarly in TNT. The larger number of unapplicable traits occurs in *Orycteropus* that is the most specialized taxon compared in our cladistic analyses.

|  | ? | -  (gaps) |  | % of data rank |  |
| --- | --- | --- | --- | --- | --- |
| Euth | 2 | 2 |  | Peris | 99,43% |
| Zhel | 50 | 1 |  | Euth | 98,86% |
| Prot | 57 | 1 |  | Arct | 98,86% |
| Arct | 2 | 0 |  | Phena | 98,29% |
| Oce | 13 | 6 |  | Hyop | 98,29% |
| Teil | 81 | 0 |  | Hyra | 98,29% |
| Phena | 3 | 0 |  | Emb | 95,43% |
| Peris | 1 | 0 |  | Macro | 94,86% |
| Rad | 86 | 0 |  | Num | 94,86% |
| Hyop | 3 | 1 |  | Phos | 93,71% |
| Todr | 79 | 1 |  | Oce | 92,00% |
| Ptol | 48 | 4 |  | Sir | 91,43% |
| Pot | 18 | 13 |  | Desm | 90,29% |
| Tub | 76 | **48** |  | Pot | 89,14% |
| Macro | 9 | 1 |  | Erit | 72,57% |
| Hyra | 3 | 1 |  | Zhel | 72,00% |
| Anth | 68 | 0 |  | Ptol | 72,00% |
| Desm | 17 | 1 |  | Prot | 68,00% |
| Sir | 15 | 1 |  | Anth | 61,14% |
| Phe | 87 | 0 |  | Tub | **56,57%** |
| Min | 103 | 0 |  | Todr | 54,86% |
| Emb | 8 | 2 |  | Teil | 53,71% |
| Erit | 48 | 1 |  | Rad | 50,86% |
| Phos | 11 | 1 |  | **Phe** | **49,14%** |
| Num | 9 | 6 |  | **Min** | **41,14%** |
