# Supplementary material for: Ocepeia (Middle Paleocene of Morocco): The Oldest Skull of an Afrotherian Mammal
Source: PLoS One. 2014 Feb 26;9(2):e89739. doi: 10.1371/journal.pone.0089739 (PMC3935939; doi:10.1371/journal.pone.0089739)
Supplement: Table S2 — Matrix of Ocepeia : 18 uninformative characters (inactived in the analysis). (DOC) [file pone.0089739.s004.doc]

Table S2. Matrix of *Ocepeia*: 18 uninformative characters (inactived in the analysis)

| K # | State in  *Ocepeia* | Remark |
| --- | --- | --- |
| 3 | 0 | Generalized trait |
| 5 | 0 | Generalized |
| 15 | 0 | Generalized |
| 20 | 0 | Generalized |
| 56 | 0 | Generalized |
| 57 | 0 | Generalized |
| 58 | 0 | Generalized |
| 64 | 0 | Generalized |
| 68 | 1 | Autapomorphy + poorly documented trait |
| 85 | 0 | Generalized |
| 95 | 0 | Generalized |
| 129 | 0 | Generalized |
| 131 | 0 | Generalized |
| 142 | 0 | Generalized |
| 154 | 1 | Autapomorphy |
| 166 | 0 | Generalized |
| 173 | ? | Generalized |
| 174 | 1 | Poorly documented trait |
| 175 | 2 | Poorly documented trait |
