# Supplementary material for: Ocepeia (Middle Paleocene of Morocco): The Oldest Skull of an Afrotherian Mammal
Source: PLoS One. 2014 Feb 26;9(2):e89739. doi: 10.1371/journal.pone.0089739 (PMC3935939; doi:10.1371/journal.pone.0089739)
Supplement: Table S3 — Matrix of Ocepeia : Irreversible characters. (DOC) [file pone.0089739.s005.doc]

Table S3. Matrix of *Ocepeia*: Features that are coded irreversible

| K # | Description | State transformation rejected | Remark |
| --- | --- | --- | --- |
| 3 | I/1 presence | 1🡪0 | No reversal state 1 |
| 5 | I/2 presence | 1🡪0 | No reversal state 1 |
| 7 | I/3 presence/development | 2🡪0 ; 2🡪1 | No reversal state 2 |
| 11 | P/1 presence/development | 3🡪0 ; 3🡪1; 3🡪2 | No reversal state 3 |
| 13 | P/2 presence/development | 3🡪0 ; 3🡪1; 3🡪2 | No reversal state 3 |
| 52 | Fusion of mandibular symphysis. | 1🡪0 | No reversal state 1 |
| 64 | Number of upper incisors | 3🡪0 ; 3🡪1; 3🡪2  2🡪1; 2🡪0  1🡪0 | No reversal all states |
| 70 | C1/Presence/Development | 2🡪0 ; 2🡪1 | No reversal state 2 |
| 72 | P1/ presence | 1🡪0 | No reversal state 1 |
| 74 | P2/ presence | 1🡪0 | No reversal state 1 |
| 131 | Lacrimal presence | 1🡪0 | No reversal state 1 |
| 175 | Inner ear – Cochlea number of turns | 2🡪0 ; 2🡪1 ; 1🡪0 | No reversal all states |
