# Supplementary material for: Ocepeia (Middle Paleocene of Morocco): The Oldest Skull of an Afrotherian Mammal
Source: PLoS One. 2014 Feb 26;9(2):e89739. doi: 10.1371/journal.pone.0089739 (PMC3935939; doi:10.1371/journal.pone.0089739)
Supplement: Table S4 — Matrix of Ocepeia : Step matrices for 19 characters, all non additive; other transformations for these characters are coded unlikely with a cost of 10 steps instead of one step. (DOC) [file pone.0089739.s006.doc]

Table S4. Matrix of *Ocepeia*: Step matrices for 19 characters*,* all non additive; other transformations for these characters are coded unlikely with a cost of 10 steps instead of one step.

| States transformation favoured (one step) |  | Characters |
| --- | --- | --- |
| 2-0-1 |  | 68, 111, 119, 122, 124, 138, 149, 168 |
| 2🡨0🡪1, 2-1 |  | 27, 90, 99, 116 |
| 3-0-1-2 |  | 105, 112, 134 |
| 1-0-2-3-4 |  | 120, 135 |
| 1-0-2-3-4  1-2 |  | 89, 107 |
