# Supplementary material for: Ocepeia (Middle Paleocene of Morocco): The Oldest Skull of an Afrotherian Mammal
Source: PLoS One. 2014 Feb 26;9(2):e89739. doi: 10.1371/journal.pone.0089739 (PMC3935939; doi:10.1371/journal.pone.0089739)
Supplement: Text S1 — Phylogenetic analysis of Ocepeia daouiensis . Part I: Studied characters of Ocepeia daouiensis. Part II: Characters matrix for analysis of Ocepeia daouiensis, characters summary list, taxa analyzed. Part III: TNT analysis, method, cladograms, and diagnose of nodes. Part IV: References. (PDF) [file pone.0089739.s007.pdf]

Ocepeia **(middle Paleocene of Morocco): the oldest skull of an afrotherian mammal**
Emmanuel Gheerbrant, Baadi Bouya, Mbarek Amaghzaz, Florent Goussard, Charlène Letenneur
Supporting Information

Text S1
Phylogenetic analysis of *Ocepeia*

Table of contents

Part I. Studied characters of *Ocepeia daouiensis* 3
Lower Dentition 3
Anterior lower dentition 3
Lower premolars 4
Lower molars 5
Upper dentition 8
Upper premolars 8
Upper molars 9
Skull 12
Part II. Characters matrix for *Ocepeia daouiensis*, characters summary list, taxa analyzed 18
Matrix 18
Characters summary list 20
Taxa analyzed 22
Part III. TNT analysis, method, cladograms, diagnose of nodes 23
1. Analysis with all characters unordered 23
1.1 Unweighted analysis 23
Cladogram 1 23
1.2 Implied weighting (standard) analysis 24
Cladogram 2 24
2. Analysis with characters ordered 25
2.1 Unweighted analysis 25
Cladogram 3 25
Synapomorphies common to 2 trees 26
2.2 Implied weighting (standard) analysis 29
Cladogram 4 29
2.3 Unweighted and partioned analysis : dental characters 30
Cladogram 5 30
2.4 Implied weighting and partioned analysis : dental characters 31
Cladogram 6 31
2.5 Unweighted and partioned analysis : Skull characters 32
Cladogram 7 - All taxa 32
Cladogram 8 - Only taxa with well known skull characters 32

3. Analysis with character ordered + step matrix 33
Character step matrix 33
3.1 Unweighted analysis 34
Cladogram 9 34
3.2 Implied weighting (standard) analysis 38
Cladogram 10 38
3.3 Unweighted and partitioned analysis: Dental and mandibular characters 39
Cladogram 11 39
3.4 Unweighted and partitioned analysis: Skull characters 40
Cladogram 12 40
4. Constrained analysis on matrix with ordered characters 41
Part IV. References 45

Part I.
Studied characters of *Ocepeia daouiensis*

The characters analyzed here are those of the matrix of Gheerbrant [1] and Gheerbrant et al. [12] that was modified and revised. Modifications of their matrix include :

- 24 characters emended : 8, 12, 15, 20, 51, 58, 60, 62, 63, 66, 80, 81, 105, 109, 116, 118, 119, 128, 133, 135, 136, 139, 143, 168;
- 36 new characters observed in *Ocepeia* (especially petrosal characters) : 3, 5, 9, 20 , 27, 50, 53, 54, 60, 67 , 94, 96, 97, 120, 122, 123, 124, 125, 126, 130, 132, 134, 138, 141, 154, 155, 156, 163, 164, 165, 167, 169 170, 171, 174, 175;
- 3 characters deleted : 78, 86, and 112 which are partly redundant or of poor significance.

Modified characters are commented, below the description of the character states. New characters are in **bold**.

Lower Dentition

Anterior lower dentition

1. Lower incisors shape and development. (0) Lower incisors small, peg like or spatulate; (1) Lower incisors higher or enlarged.
2. Lower incisors shape. (0) Lower incisors subvertical; (1) Lower incisors procumbent.
3. **I/1: (0) present; (1) absent.**
4. Development of I/1. (0) I/1 and I/2 of similar size; (1) I/1 slightly larger than I/2; (2) I/1 very enlarged; (3) I/1 hypsodont.
   Non applicable when I/1<I/2.
5. **I/2: (0) present; (1) absent.**
6. Enlargement of I/2: (0) I/1 and I/2 of similar size; (1) I/2 larger than I/1

Non applicable when I/1>I/2

7. I/3: (0) I/3 present well developed; (1) I/3 very small and labial to C/1; (2) I/3 lost.
8. C/1: (0) present and large ; (1) reduced (smaller than molars) ; (2) very small (smaller than P/2); (3) absent.
9. C/1: (0) Canine biradicular; (1) Canine uniradicular.

   Chambius has a lower canine with a single root [2].

10. P/1-P/2 diastema or C/1-P/2 diastema: (0) absent or very short; (1) present; (2) very long (in relation to the loss of C and P/1).

Lower premolars

11. P/1: (0) well developed; (1) small but more or less premolarized; (2) small and simple (basically conical); (3) absent.

Ptolemaia has lost P/1, but other species referred to ptolemaiids, such as *Cleopatrodon ayeshae* and *Qarunavus meyeri* retain P/1. The number of premolars is variable in ptomelaiids; the type specimen of *P. lyonsi* has only two premolars by contrast to most other described specimens of ptolemaiids.

12. P/1: (0) biradicular; (1) uniradicular.

This is character 10 of Gheerbrant [1], modified with deletion of state 2 because it is redundant with 11(3). State inapplicable when P/1 is lost.

13. P/2: (0) well developed; (1) small, but premolariform; (2) small and simple (basically conical); (3) absent

 P. lyonsi has lost P/2, but *P. grangeri* and other species referred to ptolemaiids retains P/2.

14. P/2: (0) biradicular; (1) uniradicular.

When P/2 is present in *Ptolemaia* (species *P. grangeri*), it is biradicular.

15. P/3: (0) biradicular and large; (1) uniradicular and smaller crown.

16. P/3: (0) metaconid absent or weak; (1) metaconid present.

17. P/3: (0) paraconid present; (1) paraconid absent or weak.

18. P/3: (0) talonid reduced (small) and simple (mostly cutting or piercing); (1) talonid developed (enlarged) and molariform with distinct basin and cusps.

19. P/3-4: (0) paraconid low; (1) paraconid high.

 Sirenians : only P/4 (homology of P/3 is uncertain in sirenians).

20. P/4: (0) biradicular and large; (1) one root and smaller crown.

21. P/4: (0) paraconid present; (1) paraconid absent or weak.

22. P/4: (0) talonid reduced (small) and simple (mostly cutting); (1) talonid developed (enlarged) and molariform with distinct basin and cusps.

23. P/4: (0) metaconid weak and distal with respect to protoconid; (1) metaconid present and lingual.

24. Lower premolars (at least P/4): (0) labial cingulum (ectocingulid) absent; (1) labial cingulum present.

Lower molars

25. M/1-3: (0) Molar pattern tribosphenic with high trigonid and sharp.
26. M/1-3: (0) no lophs; (1) lophs oblique; (2) lophs transverse.
27. **M/1-3: (0) Crown uniformly brachyodont; (1) unilateral lingual or labial hypsodonty; (2) crown uniformly hypsodont.**
   Derived embrithopods show unilateral labial hypsodonty, but primitive taxa such as *Namatherium* and *Palaeoamasia* show only incipient labial hypsodonty that is more distinct at last (larger) molar locus. In *Phosphatherium*, the lingual hypsodonty is variable.
   This character is not additive.
28. M/1-3: (0) Labial cusps (protoconid-hypoconid) larger than lingual cusps (metaconid-entoconid); (1) lingual cusps larger than labial cusps (or cusps subequal).
   In *Ocepeia*, the hypoconid is much larger than the entoconid, but the metaconid is slightly larger than the protoconid.
29. M/1-3: (0) Trigonid more or less elongated with well differentiated (functional) paracristid; (1) trigonid short, strongly compressed mesio-distally, with paracristid weakened.
30. M/1-3: (0) Paraconid present; (1) paraconid weak or absent.
31. M/1-3: (0) Premetacristid weak or absent; (1) premetacristid developed.
32. M/1-3: (0) Posmetacristid reduced or absent; (1) postmetacristid well developed; (2) postmetacristid more or less inflated as a metastylid.
   The postmetacristid is variable in desmostylians.
   This character is not additive.
33. M/1-3: (0) Protocristid depressed by a deep median notch; (1) protocristid lophid-like, with no strong notch.
34. M/1-3: (0) Hypolophid absent; (1) hypolophid present.
   Ocepeia: Hypolophid (=entolophid) incipient.
35. M/1-3: (0) Hypolophid absent or incomplete; (1) hypolophid depressed by a deep median notch; (2) hypolophid lophid-like (true lophodonty).
36. M/1-3: (0) Entocristid present; (1) entocristid reduced to absent.
37. M/1-3: (0) Entoconulid absent; (1) entoconulid present.
38. M/1-3: (0) Mesoconid absent; (1) mesoconid present.
39. M/1-3: (0) Cristid obliqua strongly oblique, joining the trigonid in its lingual part; (1) cristid obliqua joining the trigonid at its mid-width; (2) cristid obliqua joining the trigonid in its labial part.
40. M/1-3: (0) Postcristid developed, linking hypoconid, hypoconulid and entoconid; (1) hypoconulid and entoconid separated by a narrow notch (postentocristid reduced), but the labial segment of the postcristid (posthypocristid) remains well developed between hypoconulid and hypoconid; (2) hypoconulid furthermore separated from the hypoconid: labial segment of the postcristid (posthypocristid) reduced to absent; (3) hypoconulid cingular-like, completely separated from entoconid and hypoconid (postcristid reduced).
41. M/1-2: (0) Hypoconulid median; (1) hypoconulid lingual; (2) hypoconulid labial.
   This character is not additive.

42. M/1-2: (0) Hypoconulid high and poorly distal with respect to hypoconid and entoconid; (1) hypoconulid low and poorly distal with respect to hypoconid and entoconid; (2) hypoconulid low and distal; (3) hypoconulid low and cingular-like.

43. M/1-2: (0) Postentoconulid absent; (1) postentoconulid present, but weak; (2) postentoconulid well developed.

44. M/3: (0) Postentoconulid absent; (1) postentoconulid present, but weak; (2) postentoconulid well developed.

45. M/1-3: (0) Ectocingulid and/or ectostylid weak or absent; (1) ectocingulid present.

46. M/1 trigonid/talonid relative width: (0) trigonid narrower than talonid; (1) trigonid subequal to the talonid; (2) trigonid wider than the talonid.

47. M/2 trigonid/talonid relative width: (0) trigonid narrower than talonid; (1) trigonid subequal to the talonid; (2) trigonid wider than the talonid.

48. M/3 trigonid/talonid relative width: (0) trigonid wider than the talonid; (1) trigonid subequal to the talonid; (2) trigonid narrower than the talonid.

49. M/3: (0) M/3 of similar size to M/2, or slightly larger; (1) M/3 smaller than M/2; (2) M/3 significantly larger than M/2, which is larger than M/1.
This character is not additive.

50. M/3: (0) two roots; (1) two roots, but roots fused in higher part ; (2) one root.
Taxa that have M/3 with more than two roots are scored inapplicable.

Dentary

51. Extension of mandibular symphysis: (0) short: extended to P/2 or more anterior; (1) moderately long: extended between P/2 and P/3 ; (2) long : extended to P/3 or more posterior.
Palaeoamasia : the mandibular symphysis extends up to P/4 [4]. *Ocepeia*: the mandibular symphysis is short, but it extends up to P/3 because of the loss of P/1-2 and compression of the anterior part of the tooth row. Macroscelidea: *Herodotius* [4]. Hyracoidea : *Microhyrax*.

52. Mandibular symphysis fusion: (0) unfused (synchondrosis); (1) fused.
Embrithopods : *Arsinoitherium*.

53. **Anteriormost mental foramen : (0) below P/1 or more anterior ; (1) below P/2; (2) below P/3 ; (3) posterior to P/3.**

Ocepeia: The anterior mental foramen varies between anterior and below P/3, but seems under P/3 in most specimens (P/2-1 lost). *Numidotherium* has only one mental foramen below P/3-4 of which the homology is tentative here. The desmostyle *Behemotops* has two mental foramina below P/3 and other ones more anterior but of unkown exact location [6]. Primitive sirenians such as *Prorastomus* have numerous mental foramina of which the homology is uncertain.

Wible et al. [7]: character 129.

54. Posteriormost mental foramen: (0) below canine and anterior premolars; (1) below penultimate premolar; (2) below ultimate premolar; (3) at ultimate premolar and first molar junction or more posterior.

Taxa with only one mental foramen are scored inapplicable.

Wible et al. [7]: character 130.

55. Horizontal ramus (body): (0) low; (1) high

56. Position of the coronoid apophysis (adult): (0) rising at level of M/3, or more distally; (1) rising anteriorly to M/3.
    In the primitive macroscelidean *Chambius*, the coronoid apophysis rises distinctly posteriorly to M/3.

57. Orientation of the coronoid apophysis: (0) anterior margin vertical or posteriorly canted; (1) anterior margin anteriorly canted.

58. Development of the coronoid apophysis: (0) coronoid apophysis very high above tooth row (as high or higher than M/3-P/4 length) ; (1) coronoid apophysis moderately high above tooth row ; (2) coronoid apophysis strongly reduced to absent.

This is character 51 of Gheerbrant [1], modified 1) to be independent from the vertical location of the articular condyle, and 2) with the addition of the state 2.

59. Mandibular condyle: (0) at level or low above the dental row; (1) moderately high above the dental row (one molar length); (2) very high above the dental row (more than one molar length), and closer to coronoid process apex than to dental row.
    Embrithopods : *Arsinoitherium*.

60. Mandibular condyle shape: (0) ovoid (or plate-like); (1) transversely elongate and cylindrical; (2) antero-posteriorly elongate.

Embrithopods: Arsinoitherium.

Wible et al. [7]: character 149; Seiffert [8]: character 287.

61. Coronoid foramen: (0) absent; (1) present.

62. Angular process: (0) thin stick-like or hook-like distally protruding angular process; (1) broad and rounded distally projecting angular process; (2) mandibular angle broad and round without angular process.

Metoldobotes has a broad and rounded angular process [8], but in other macroscideans it is thin. Seggeurius is in the state 1, but more derived hyracoids have a broadly expanded angle that is not protruding posteriorly. Arsinoitherium is in the state 2, but the state of more primitive embrithopods such as palaeoamasiids and *Namatherium* is unknown.

63. Anterior coronoid fossa (retromolar fossa): (0) absence of fossa on the anterior margin of the ascending ramus (coronoid process); (1) presence of a retromolar fossa on the ascending ramus behind the M/3, which is delimited medially by the coronoid crest of the ascending ramus and laterally by the sharp mesio-laterally salient masseteric crest; (2) fossa enlarged, as the result of the posteriorly recurved coronoid crest (at basis) and of the sharper masseteric crest.

Upper Dentition Anterior dentition (upper incisors and C1/)

64. Number of upper incisors: (0) three; (1) two; (2) one; (3) none.

65. Relative size of upper incisors: (0) small; (1) large.

66. Relative size of I1/: (0) not enlarged (≤ I2/); (1) I1/>I2/; (2) I1/ greatly enlarged I1/>>I2/ (I1/ tusk like).

67. Relative size of I2/: (0) not enlarged (≤ I1/); (1) I2/>>I1/.
   The relative size of I2/ in primitive hyracoids and generaly in primitive paenungulates is unknown. Embrithopods : *Arsinoitherium*.

68. Relative size of I3/ : (0) I3/ similar in size to I2/ and I1/ ; (1) I3/ very small, much smaller than I2/ and I1/ ; (2) I3/ larger than I2/ and I1/.

This character is added with respect to Gheerbrant (2009), especially in order to take into account state (2) of *Ocepeia*.

This character is not additive.

69. Upper diastema: (0) small or absent; (1) developed but short; (2) elongated (I2/-P2/).

70. C1/: (0) large (>> premolars) ; (1) medium to small (similar or smaller to premolars and incisors); (2) absent.

71. C1/: (0) two roots; (1) one root.

Upper premolars

72. P1/: (0) present; (1) absent.

73. P1/: (0) two roots; (1) one root
   Arsinoitherium has one root at P1/, but the character is unknown in primitive embrithopods.

74. P2/: (0) present; (1) absent.

75. P2/: (0) two roots; (1) three roots; (2) one root.
   Arsinoitherium has two roots at P2/, but this state state might not be representative of the primitive condition of the embrithopods.

This character is not additive.

76. P2/: (0) protocone absent, crown narrow transversely; (1) protocone present, crown slightly developed transversely; (2) protocone large, crown as long as wide.

77. P3/: (0) two roots; (1) three roots; (2) one root.

Desmostyles = *Ashoroa*.

78. P3/: (0) protocone absent or reduced; (1) protocone present; (2) protocone well developed, crown as wide as long.

79. P3/: metacone: (0) absent; (1) present and small; (2) large.

80. P3/: conules: (0) absent; (1) present.

81. P4/: conules: (0) absent; (1) present.

82. P4/: (0) two roots; (1) three roots; (2) four roots.

83. P4/: metacone: (0) absent; (1) small; (2) large.

84. P4/: metacone: (0) absent; (1) connate to paracone; (2) well developed and separated from paracone.

85. P4/: hypocone: (0) absent; (1) present.

86. P4/: (0) no loph; (1) at least one loph present.

87. P4/ (and P3/ when protocone is developed): (0) postprotocrista distinct; (1) postprotocrista absent or weak.

Upper molars

88. M1-3/ : (0) More or less puncturing morphology with high and pointed cusps; (1) bunodont morphology with low and inflated crown and cusps.

89. M1-3/ : (0) Strictly bunodont pattern (loph absent); (1) bunodont bilophodont pattern, with weak metaloph and protoloph; (2) bunodont bilophodont pattern, with developed (crest-like) metaloph and protoloph; (3) true lophodont pattern; (4) bunolophodont pattern.
Homology of the upper molar pattern of *Orycteropus* is unknown, but the bilobed occlusal outline suggests vestigial bilophodonty and presence of hypocone. Embrithopods are coded inapplicable because their bilophodonty is not homologous with other taxa (although it is probably derived from state 1).

This character is not additive.

90. M1-3/ : (0) No loph; (1) lophs transverse; (2) lophs oblique.

Embrithopods are coded inapplicable because their bilophodonty is not homologous with other taxa.

This character is not additive.

91. M1-3/ : (0) Parastyle mostly labial to the paracone; (1) parastyle mesial.

92. M1-2/ : (0) Parastyle large on M1-2/; (1) parastyle small on M1-2/.

93. M1-2/ : (0) Ectocingulum reduced, or thin and isolated; (1) ectocingulum inflated and continuous with mesial and distal cingula.

94. M1-2/ Stylar shelf development: (0) Stylar shelf wide (> 50 % molar width) ; (1) Stylar shelf moderately wide (50 to 25 % of molar width); (2) Stylar shelf reduced (< 25% molar width); (3) Stylar shelf absent.

The hypertrophied stylar shelf (state 0 here) is an important autapomorphy of the embrithopods, corresponding to a reversed trait that returns to primitive state of pretribosphenic therians (e.g., peramurids). In *Phenacolophus* the stylar shelf is typically wider posteriorly than anteriorly in the molars, in relation with the paracone located more labial than the metacone.

Wible et al. [7]: character 65.

95. M1-3/ : (0) Pericone absent; (1) present.

96. M1-3/ : (0) Cingulum lingual (pre- and postcingulum) absent; (1) cingulum lingual present (pre- and postcingulum).

97. M1-3/ : (0) No hypocone ; (1) hypocone small (<<protocone); (2) large hypocone (close in size or larger than protocone).

The hypocone in this character is derived from the postcingulum. Homology is unknown in *Orycteropus*, but the bilobed shape of upper molars suggest vestigial bilophodonty and presence of

hypocone. In embrithopods the hypocone is vestigial, as the protocone (probably secondarily reduced).

98. M1-3/ : (0) Mesostyle absent or weak; (1) present and inflated.

Behemotops has a small but inflated mesostyle.

99. M1-3/ : (0) Mesostyle absent or weak; (1) mesostyle present and close to paracone and metacone; (2) mesostyle present and shifted labially with respect to paracone and metacone.

In *Phenacolophus* the pattern is singular : the mesostyle is shifted labially with respect to metacone, but not to paracone.The combination of the characters and states 99-2 and 101-2 correspond to an ectoloph selenodont with advanced dilambdodont pattern; it is specialized for an enhanced shearing function from phase I of mastication and is known both in follivorous and insectivorous taxa.

This character is not additive.

100. M1-3/ : (0) Centrocrista present; (1) reduced to absent.

The reduction of the centrocrista is not homologous in tenrecids and paenungulates.

101. M1-3/ : (0) Centrocrista rectodont and not linked to mesostyle; (1) centrocrista linked to the lingual flank of the mesostyle and more rectodont than dilambdodont; (2) centrocrista extensively linked to the mesostyle and noticeably dilambdodont (ectoloph selenodont); (3) centrocrista hyperdilambdodont with lingual migration of paracone and metacone.

State 3 is typical of the embrithopods [9].
Absence of centrocrista: character not applicable.

102. M1-3/ : (0) Postmetacrista well developed and predominantly transversal; (1) postmetacrista weak and predominantly longitudinal.

In embrithopods the postmetacrista is reduced, but transverse.

103. M1-3/ : (0) Preparacrista well developed and predominantly transversal; (1) preparacrista small and predominantly longitudinal; (2) Preparacrista absent or very weak

104. M1-3/ : (0) Metacone smaller than paracone but well developed on M3/; (1) metacone weak or absent on M3/.

105. M1-3/ : (0) Conules well developed; (1) conules small; (2) conules absent (with possible occurrence of a slight paraconular swelling) ; (3) conules inflated and bulbous.

This character is not additive.

Wible et al. [7]: character 76.

106. M1-3/ : (0) Postprotocrista developed; (1) postprotocrista reduced or absent.

State 1 is related to lophodont pattern, although it is also found in non lophodont taxa such as *Hyopsodus.*

107. M1-3/ : (0) Prehypocrista and metaloph absent or reduced; (1) prehypocrista absent or weak, metaloph formed by transversal alignment and close juxtaposition of hypocone, metaconule (if present) and metacone (2) prehypocrista developed forming the metaloph and joining metaconule or metacone base; (3) prehypocrista forming the metaloph and joining the metacone apex (true lophodonty).

Embrithopods are coded non applicable because of the secondary reduction of the hypocone, and their non homologous bilophodonty (= hyperdilambdodonty).

This character is not additive.

108. M1-3/ : (0) Interloph not differentiated; (1) interloph developed transversally (extended lingually to a more or less developed entoflexus)

109. M1-2/ : (0) Distocrista present between postcingulum and hypocone; (1) Distocrista absent, postcingulum independant from hypocone.

Polarity of this character is redefined with respect to Gheerbrant et al. [2] and Gheerbrant [1]. The distocrista is primitively linked to the hypocone. Taxa without hypocone are coded not applicable. State 1 is the precursor of the development of a third (distal) loph.

110. M1-3/: (0) Postentoconule (hypostyle) absent; (1) postentoconule (hypostyle) present.

111. M1/ and M2/: (0) M1/ and M2/ of roughly similar size; (1) M1/ significantly smaller than M2/; (2) M1/ significantly larger than M2/

This character is not additive.

112. M2/ and M3/ : (0) M2/ and M3/ of roughly similar size; (1) M3/ smaller than M2/; (2) M3/ strongly reduced; (3) M3/ significantly larger than M2/ (molar size increase posteriorly).

Primitive hyracoids such as *Microhyrax* have a small M3/, in the same proportion as to *Eritherium*.

This character is not additive.

113. M1-2/: (0) one simple lingual root; (1) lingual root enlarged and incipiently divided with a median sulcus; (2) at least 2 lingual roots (hypocone root).

Chambius (Macroscelidea) has two lingual roots which are partly fused. *Namatherium* seems to have one enlarged lingual root at M1/, but *Palaeoamasia* has two lingual roots. According to McKenna et al. [10], *Radinskya* has four roots at its M1/. *Microhyrax* is charaterized by a small M3/ which is probably representative of the ancestral morphotype of the Hyracoidea.

114. M3/ : protocone root (0) small and simple; (1) enlarged.

115. M3/: hypocone root: (0) absent; (1) distal root present; (2) mesial and distal roots present.

In *Namatherium* the hypocone roots are fused in the higher part below the crown, but separated in their lower part [11]. *Palaeoamasia* has only two lingual roots, one below the hypocone (its vestigial remnant), and the other, enlarged, below the protocone. Ptolemaids have a single root at M3/.

116. M1-3/: (0) Crown uniformly brachyodont; (1) unilateral lingual or labial hypsodonty; (2) crown uniformly hypsodont.

This is character 106 of Gheerbrant [1], modified with addition of state 2.

Derived embrithopods such as *Arsinoitherium* show unilateral labial hypsodonty, but primitive taxa such as *Namatherium* and *Palaeoamasia* show only incipient labial hypsodonty that is more distinct at last molar locus (larger teeth). In *Phosphatherium*, the lingual hypsodonty is variable. *Teilhardimys*, as *Louisinina*, is characterized by unilateral labial hypsodonty.

This character is not additive.

117. M1-3/: (0) Occlusal outline extended transversally; (1) occlusal outline narrower, squared or slightly elongated.

Skull

118. <u>(0) Reduced pneumatization of skull bones; (1) significant pneumatization (developed sinuses and diploe); (2) extensive pneumatization.</u>

Ocepeia is remarkably specialized in the extensive pneumatization of several bones of posterior part of the skull; this is especially true for the supraoccipital, but also for the parietal, the basisphenoid, the basioccipital and the periotic.

This is character 108 of Gheerbrant [1], modified with the addition of state 2 (*Ocepeia* condition).

119. Rostrum elongation: (0) Preorbital length relative to the total skull length between 30% and 50 %; (1) Preorbital length relative to the total skull length > 50 %; (2) Preorbital length relative to the total skull length < 30 %.

This is character 2 of Gheerbrant et al. [2], modified for better accuracy. The primitive eutherian condition of an elongated rostrum is seen in *Asioryctes* (40%), *Kennalestes* (39%), and *Arctocyon* (34%). *Ocepeia* is surprisingly primitive in this character (preorbital length relative to the total skull length = 37 %); in this regard, it is best characterized by a wide rostrum (see character 120). *Arsinoitherium* is primitive in this state, but possibly secondarily by comparison to *Namatherium*. State 1 is found in some specialized eutherians such as *Zalambdalestes*.

This character is not additive.

120. Rostrum relative width to length - ratio of width of the snout at canine level to preorbital length: (0) between 30% (included) and 50%; (1) <30 % ; (2) between 50% (included) and 70% ; (3) between 70% (included) and 90% ; (4) > 90%.

Arsinoitherium is in the state 0, but this might not be representative of the primitive condition of the order Embrithopoda in view of *Namatherium* which seems to have a shorter snout (although broken anteriorly in the only known specimen). *Ocepeia* is very derived (82 %; autapomorphy) in this character, being characterized by a primate-like robust short and broad snout. State 1 in found in some specialized eutherians such as *Zalambdalestes*.

This character is not additive.

121. (0) Upper tooth row not extended more distally than mid skull length; (1) upper tooth row very extended distally, up to the two thirds of the skull length.

122. Palatine development: (0) Palatine anteriorly extended to M1/ level; (1) Palatine short, more posterior than M1/; (2) Palatines more anterior than M1/.

In *Ocepeia*, the palatine is very short, and it is extended between rear of M3/ and M2/ levels.

This character is not additive.

Wible et al. [7]: character 189.

123. **Major palatine foramen: (0) within palatine; (1) between palatine and maxilla; (2) within maxilla; (3) multiple small foramina; (4) absent.**

This character is not additive.

Wible et al. [7]: character 188.

124. Location of anterior margin of the choanae: (0) at M3/ level, (1) posterior to M3/, (2) anterior to M3/.

This character is not additive.

125. **Postpalatine torus : (0) present, (1) absent.**

Seiffert [8]: character 304.

126. (0) Nasal cavity narrow and not high anteriorly; (1) Nasal cavity wide and high.

127. (0) Nasal long and located anterior to the orbit; (1) nasal short and posteriorly shifted.

128. (0) Nasal fossa anterior; (1) nasal fossa moderately retracted; (2) nasal fossa strongly retracted.

Character modified from Gheerbrant et al. [2], Gheerbrant [1]. According to Pickford et al. [11], the nasal fossa is retracted in *Namatherium*.

129. (0) Premaxillary-frontal contact absent (separated by the nasal); (1) premaxillary-frontal contact present.

130. (0) No contact between frontal and maxillary; (1) Frontal and maxillary contact present

Wible et al. [7]: character 173.

131. (0) Lacrimal present; (1) lacrimal absent.

In the embrithopod *Namatherium* the lacrimal is not distinct, but the lacrimal foramen is present.

132. (0) Lacrimal facial process present and large; (1) Lacrimal facial process reduced along orbital rim or absent.

133. (0) Lacrimal tubercle present; (1) lacrimal tubercle absent.

The presence of a lacrimal tubercle is considered primitive in eutherians where it it known for instance in leptictids [7]. This polarity is followed by contrast to Gheerbrant et al. [2] and Gheerbrant [1].

134. (0) Lacrimal foramen anterior to orbit (facial); (1) lacrimal foramen in the orbit rim; (2) lacrimal foramen in the orbit; (3) lacrimal foramen absent.

The lacrimal foramen is absent in *Arsinoitherium*, but it is present in *Namatheriu*m and located in the orbit [11]. The lacrimal foramen is present in *Prorastomus*. The absence of a lacrimal foramen is specialized in *Potamogale* with respect to other non-aquatic tenrecids.

This character is not additive.

Seiffert [8]: character 306.

135. (0) orbit very posterior, above M3/ or more caudal; (1) orbit posterior, above the anterior molars (M1-2/); (2) orbit shifted anteriorly with anterior rim above P4/-M1/ level, and closer to infraorbital foramen; (3) orbit above premolars; (4) orbit anterior to premolars.

This character is modified following Seiffert [8].

The embrithopod *Namatherium* has an orbit located close to P4/-M1/level, and the infraorbital canal is short. *Dimaitherium* has an orbit located above P4/ anterior part [12].

This character is not additive.

136. (0) Orbit bordered ventrally mostly or entirely by the jugal that joins the lacrimal (reduced zygomatic process of the maxillary); (1) orbit bordered ventrally partly by a short process of the maxillary which extends between the lacrimal and jugal; (2) maxillary forms the antorbital rim (zygomatic process of the maxillary), the jugal being restricted distally to the orbit.

This is character 11 of Gheerbrant et al. [2], and 118 of Gheerbrant [1], modified following Cox [13].

In hyracoids, there is an incipient anterior reduction of the jugal that is less developed than in the primitive eutherian condition. The state varies from a small contact (*Saghatherium*) to absence of

lacrimal-jugal suture (*Procavia*, *Antilohyrax* and *Dimaitherium*) [1]. Accordingly, the state is coded polymorphic [01] in hyraxes. In *Ocepeia* there is a short maxillary process developed in the orbit rim, so the jugal is slightly withdrawed posteriorly and not in contact with lacrimal. The zygomatic process of the maxilla is indeed more developed in *Ocepeia* than in *Procavia*. In *Namatherium*, the orbit anterior rim is made mostly from the maxillary. The jugal is lost in *Potamogale* and other tenrecids.

137. (0) Infraorbital foramen small; (1) infraorbital foramen large.

138. (0) Infraorbital foramen above last premolar; (1) Infraorbital foramen anterior to last premolar; (2) Infraorbital foramen below M1/ or more posterior .

The primitive eutherian condition might be in fact in the state 1 (*contra* [7]). This character is not additive.

Wible et al. [7]: character 165.

139. (0) Infraorbital foramen anterior to the orbit (more than one tooth length), long infraorbital canal; (1) Infraorbital foramen close to the orbit, short infraorbital canal; (2) Infraorbital foramen below to the orbit, short infraorbital canal.

140. (0) Submaxillary fossa absent; (1) submaxillary fossa present

141. (0) Frontal similar or a little shorter than parietal; (1) frontal very shortened: less than half length of parietal; (2) frontal very elongated: longer than parietal.

In *Ocepeia*, the frontal is very short with a length of about 1/3 that of the parietal. This character is partly equivallent to character 43 of Asher et al. [14], and character 226 of Wible et al. [7].

142. (0) Tuber maxillae (maxillary tuberosity) reduced; (1) tuber maxillae large (sometime inflated).

143. (0) Tuber maxillae mostly ventral or anterior to the orbit; (1) tuber maxillae noticeably posterior to the orbit.

The primitive embrithopod *Namatherium* is characterized by a tuber maxillae posterior to the orbit, by contrast to *Arsinoitherium*.

144. (0) Ascending process of the palatine present between the maxillary and the frontal in the orbito-temporal fossa; (1) ascending process of the palatine palatine reduced (small) in the orbito-temporal fossa; (2) ascending process of the palatine absent in the orbito-temporal fossa (= contact between frontal and maxilla in orbito-temporal fossa).

145. Morphology of the zygomatic arch: (0) Zygomatic arch narrow and slender without distinct ventral process at maxillary suture; (1) Zygomatic arch high dorso-ventrally with ventral process at maxillary suture.

The ancestral condition of this character in hyracoids needs to be confirmed.

146. (0) Jugal restricted distally to the anterior part the glenoid fossa; (1) jugal extended distally up to the distal part the glenoid fossa.

147. (0) Postorbital constriction strong; (1) postorbital constriction weak or absent.

148. (0) Postorbital process of the frontal developed, inflated; (1) postorbital process of the frontal reduced or absent.

149. (0) Dorsal postorbital process of the jugal present but weak; (1) dorsal postorbital process of the jugal absent; (2) dorsal postorbital process of the jugal very developed (trend to orbit closing).

This character is not additive.

150. (0) Zygomatic arch poorly divergent laterally; (1) zygomatic arch divergent laterally.

The embrithopod *Namatherium* has widely divergent zygomatic arches [11].

151. (0) Squamosal: Cerebral part not extended dorsally; (1) squamosal: Cerebral part extended and inflated (=scale).

152. (0) Zygomatic process of the squamosal moderately developed laterally; (1) Zygomatic process of the squamosal noticeably expanded laterally and robust.

153. (0) Parietal-alisphenoid contact; (1) frontal-squamosal contact.

154. (**0) Parietal not extended more anteriorly than alisphenoid; (1) Parietal extended more anteriorly than alisphenoid with an orbitosphenoid contact**.

In *Ocepia*, the parietal-orbitosphenoid suture, together with the long parietal-alisphenoid, is related to the autapomorphic anterior extension of the parietal.

155. (0) Alisphenoid canal absent; (1) alisphenoid canal present.

The alisphenoid canal is primitively absent in Eutheria [7]. Its presence was decribed as a basal paenungulate trait [15, 16]. Its absence is secondary within paenungulates such as advanced proboscideans. The alisphenoid canal is variable in arctocyonids (present in *Neoclaenodon*).

Wible et al. [7]: character 248.

156. (0) Foramen rotundum and sphenorbital fissure confluent; (1) foramen rotundum separated from the sphenorbital fissure.

The foramen rotundum is separated from the sphenorbital fissure in *Arctocyon* according to Russell [17]. In both *Ocepeia* and *Phosphatherium*, the foramen rotundum seems confluent with sphenorbital fissure, but these elements are poorly preserved in known material, which makes this character uncertain.

Cox [13]: character 19; Froehlich [18]: character 10.

157. (0) External auditory meatus poorly elevated above the tooth row; (1) External auditory meatus high above the tooth row (close to the orbit level).

The condition cannot be checked in *Namatherium* because the skull is broken in this area.

158. (0) Post-tympanic process reduced, external auditory meatus opened ventrally; (1) Post-tympanic process developed, external auditory meatus still opened ventrally; (2) Post-tympanic process developed joining the postglenoid process, external auditory meatus ventrally enclosed

159. (0) Sagittal crest present; (1) sagittal crest reduced/absent.

160. (0) Nuchal crest present; (1) nuchal crest reduced/absent.

161. Periotic: (0) Periotic mastoidy: Mastoid exposure large between squamosal and exoccipital; (1) Periotic amastoidy: Mastoid exposure absent.

According to Savage et al. [19], the structure is amastoid in the primitive sirenian *Prorastomus*.

162. Periotic: (0) Pars mastoidea small; (1) Pars mastoidea larger than pars cochlearis.

163. Periotic: (0) Fenestra vestibuli medium to large; (1) Fenestra vestibuli very small (<< f. cochleae).

The fenestra vestibuli is small in early proboscideans such as *Phosphatherium* and *Numidotherium* [20].

164. Periotic: (0) Fenestra vestibuli round with low stapedial ratio (< 1.8); (1) Fenestra vestibuli elliptical with high stapedial ratio (>1.8).

A high stapedial ratio is a derived traits of the placentals, but low stapedial ratio is known in several primitive eutherians lineages, such as *Maelestes*, *Asioryctes* and leptictids. Some placentals such as *Arsinoitherium* and sirenians [21] also have a round fenestra vestibuli.

Wible et al. [7]: character 275.

165. Periotic : (0) Fenestra cochleae postero-medial to fenestra vestibuli; (1) Fenestra cochleae posterior to fenestra vestibuli.

The fenestra cochleae is absent in *Arsinoitherium*, but the primitive condition of the embrithopods in unknown.

Wible et al. [7]: character 302.

166. Periotic (pars cochlearis): (0) Fenestra rotunda (f. cochleae rotunda) and cochlear canal (cochleae canaliculus) present; (1) Fenestra rotunda and cochlear canal absent, perilymphatic foramen present.

In embrithopods, the presence of a perilymphatic foramen is based on *Arsinoitherium*, which might not be representative of the primitive condition, as for sirenians and proboscideans. This character is not preserved in *Namatherium*.

167. Periotic : (0) Subarcuata fossa present and deeply excavated ; (1) Subarcuata fossa present but poorly excavated; (2) Subarcuata fossa absent.

Embrithopods : *Arsinoitherium* (derived from the ancestral morphotype of the order?).

168. (0) Postglenoid foramen medial; (1) Postglenoid foramen distal; (2) Postglenoid foramen absent.

The polarity of the states of this character is revised here with respect to Gheerbrant et al. [2] and Gheerbrant [1]. The medial location of the postglenoid foramen seen in *Ocepia* is generally considered derived as in *Phosphatherium* (Gheerbrant et al. 2005). However, this character occurs in several Cretaceous eutherians such as in *Daulestes*, *Maelestes*, *Zalambdalestes*, which suggests it is instead primitive. The postglenoid foramen is absent in tubulidentates (*Orycteropus*), hyracoids and *Arsinoitherium* [9].

This character is not additive.

169. (0) Tegmen tympani small or absent; (1) Tegmen tympani inflated, forming large and robust barrel-like bony structure.

An inflated or hyper-inflated tegmen tympani is a derived character known in several placentals taxa such as sirenians and cetartiodactyls (anthracotheriids, hippopotamids, cetaceans) ; in cetartiodactyls, it is usually related to underwater hearing [22, 23, 24]. It is inflated in the modern sirenians, but not in *Prorastomus* [21]. In the macroscelidean *Chambius*, the tegmen tympani appears moderately inflated [25,26].

170. (0) Jugular foramen small (subequal to fenestra cochleae); (1) Jugular foramen large (> > fenestra cochleae).

Embrithopods : *Arsinoitherium*.

Wible et al. [7]: character 312.

171. (0) Absence of midline keel at ventral surface of basioccipital; (1) midline keel of basioccipital weak but present developed; (2) midline keel of basioccipital well developed.

Seiffert [8]: character 354.

172. (0) Hypoglossal foramen present and isolated; (1) Hypoglossal foramen coalescent with *f. lacerum posterius* (= f. *metoticum*).

173. (0) Ethmoid foramen far anterior to optic foramen, located median in the orbito-temporal fossa; (1) Ethmoid foramen posteriorly shifted.

174. Inner ear – Cochlea : (0) Secondary bony spiral lamina present on the basal turn; (1) Secondary bony spiral lamina present on basal and higher turns.

175. Inner ear – Cochlea: (0) Number of turns of the cochlea ≤ 1.5; (1) Number of turns of the cochlea between 1.5 and 2 turns; (2) Number of turns of the cochlea ≥ 2.

The number of turns of the cochlea in modern sirenians is exceptionally small among the placentals [27].

Wible et al. [7]: character 276.

Part II.
Characters matrix for *Ocepeia daouiensis*,
characters summary list, taxa analyzed

Matrix

The characters matrix analyzed below corresponds to the revised matrix of Gheerbrant et al. [2], Gheerbrant [1], with character corrections and additions (Part I), and with addition of new basal taxa. The matrix include the 175 characters described above, of which 18 are uninformative (Table S2). Characters are treated as additive, except when mentioned.

Eutheria
0000000000000000000000000000000000000000000000000[01]10000[01]00[01]00000000000000[01]0000[01]000
000-0000100000000000000-0000000000000[01]00000000001000100000000000000000000000[01]00000000000

Zhelestidae
0000000[01]1000000??000[01]110[01]00[01]0000000000[12]01000[01]11100100[12]000010010????10[01]0?000000
00111000[01]00100201000000[01]00000-
01100000???0??0???00?0???[02]?1100?00???0??????????????00000?0??0?00

Protungulatum
000000001011000000000010100100010001001 0[01]00010100000[01][12]000020020?????010?0??1000010000010 0[
01]0120100000[01]100000-01100000???????????????????????002???????????????0001000?0??????

Arctocyonidae
000000001[01][01][01]0000[01]00000[01][01]10000011000000[12]0100010[01][01]0000020000[12][01]0[01]0000000
01[01]1000100001000001001[01]0201[01]0000[01]1000000011[01][01]00000000000000101110001001000000 0[01]0
0000[01]10000000100010?0000?

Ocepeia
00000010113-3-0010101000100[01]001201001110110000[12]10021230000110 1101?111011-1--
12000100000100001101012021001000-
0100000020301100100000 0?2010100100?1?01000?010?0000111100001120?12

Teilhardimys ???????0101000001010100012101110010110110100[01]02010000300????0?0??????????0121210112[
12]00011111121120000111[12]121001200011???

Phenacodonta
000000001001000[01]0000011[01]10000002000[01][01]1011?0010[12]0100002000021010000 0[02]00100000112011 2
20001[01]0101201211011100[01]1100011[01]000002022010000[01]01[01]200[01][012]0010000000[01]00000100100
000000?1012000?

Perisssodactyla
00000000100[01]000101000111210001020111101202 0012[12]020010[12]0000210[12]00000[02]00100000122[01]112
200012210120120000110012110111100001 00[02]1[02]1000000002[01]00000011000000000101100010001 00?101000
02

Radinskya ???????????????????????????0??0000012??1121000122111
2012000011031211011 21?000??02?2000000000?000?0000000000?000??100001000?00?1??0000?

Hyopsodus
000-
0101102110000000011010001[01][01][02]000100011000[01]0[12]00000[01]20000110100110001101000100001 00000
10011020120000 1100[01]00001100000000200000?0001000200110 01000[01]0100011001000100010000101200??

Todralestes ???????0111100000010001000000000000000010000001 1000?022000000000??????????00?0??00100000
0-00002011000000010000000000000????????????????????????0????????????????????????????????????

Ptolemaia
???????0103-[03]00000100010102000000000000200000011012001[12]00000100000100001 1-
00?12??111100010011030000000 1102000-0220-02000?0??1?000??0??1001100000???00????????0000???????????????

Potamogale
[01]00-0101103-0000100000101000000000000000--0002200020[01]300000100001200010 1-000120001000000-
00010000000100012000-01100000000102311000001-131211201000--11--1-001000010??1000101110??

Orycteropus
--1-1-23-23-3-101--0------2-----------------001012000?0002200103-----2-1-1--2----0???0?1???????????????????????11---2101110410100000010100200000000001000110[01]0000100001002201010??
Macroscelidea
010[02]0-011[01]1[01]000[01]0110011010100001000010111100[01]0201010[01]2?00[01]2[01]0[01]0012000[01]0000001111112[12][01]001111112012000011110210012[12]001100002[01]1100000001210[01]00000000010101000[01]00?000??1?0011?[12]00??
Hyracoidea
010-0[01]010000000100100010220111020111011002001[01]102001010000211[12]1012?001[01]0001212100121000112[01][01]1101212021002121001[13]2110100000101000010[01]022[01]010000[01]0010020000[01]11010110010002012000?
Anthracobunia
0?0000001[01]0000010[01]1001112201111[12]011[01]0003030210[01][12]2[01]01??0000???10??????010100012111121001112111[23]01200001103111011311101?????????????????10??????????????????????????????????????
Desmostylia
110000001[01][12]1000000?000112201110[01]0111001303[01]11[01][01]22001?110001112001000?010?00011001100001112111[23]012[01]01-1200111011011?010??0021?10001011?[23]010[12]0001000000?01??101200100??0????200??
Sirenia
1100000[01]001021????110?11220111000111001303?200102021??100021120012000100102000???0?????111111[23]012001-1200111011011101000021[01]11121101123011210010100001010010110110001021[01]1000?0
Phenacolophus
10000001100100000000????3101000211100003130010[01][12]200???100?????0??????????020?000???????1111[01]1[12]0121[12]0201000110003[12]1[01]01??????????????????0???????????????????????????????????????
Minchenella ???????11???00011110011121010102011100031302122020????1???????0?????????????????????????11200120120000010002[01]001311001???
Embrithopoda
11010-0110000001011001113111010111210003130010[01]02021?210002112101?0001101000120001[01][01]0[01]11--00-0012120300021-10013[12]1[12][01]1000001011110101122[12]1010001110000101001012?01000?12201111??
Eritherium
11010-121021110010101010220111[01]1011101232322[01]0201000020?????11[12]?????0??0100012000100001111111[12]012110112021[12]10111111010??00???1000?0??2211100?01?1?00?10???????0????????????????
Phosphatherium
11020-22?03-1100101?101032[01]111101120[01]1232311000[01]2020[01][23]000011112?????01101000121001110111311103012110112021310113111[01]10220210110001011231110100121?000101101?010011001000001000?
Numidotherium
11020-23-23-10001[01]10111032011110112000132300000[01]20213-101021112010102111-011121001110111311103012001-12021310113212011201?11111001011232111100121100111110111200111?10?00021002

Characters summary list (in bold new characters)

1. Lower incisors shape and development
2. Lower incisors shape.
3. I/1 presence
4. Enlargement of I/1.
5. I/2 presence
6. Enlargement of I/2.
7. Development of I/3.
8. Development of C/1.
9. C/1 root(s).
10. Anterior lower diastema.
11. Development of P/1.
12. P/1 root(s).
13. Development of P/2.
14. P/2 root(s).
15. P/3 root(s) and development.
16. P/3 metaconid development.
17. P/3 paraconid development.
18. P/3 talonid development.
19. P/3-4 paraconid position.
20. P/4 root(s) and development.
21. P/4 paraconid development.
22. P/4 talonid development.
23. P/4 metaconid.
24. Lower premolars cingulum.
25. Lower molar pattern.
26. M/1-3 lophs.
27. M/1-3 crown height
28. M/1-3 cusps.
29. M/1-3 trigonid.
30. M/1-3 paraconid.
31. M/1- 3 premetacristid.
32. M/1- 3 postmetacristid.
33. M/1- 3 protocristid.
34. M/1- 3 hypolophid development.
35. M/1- 3 hypolophid shape.
36. M/1- 3 entocristid.
37. M/1- 3 entoconulid.
38. M/1- 3 mesoconid.
39. M/1- 3 cristid obliqua.
40. M/1- 3 postcristid.
41. M/1-2 hypoconulid position.
42. M/1-2 hypoconulid development.
43. M/1-2 postentoconulid:
44. M/3 postentoconulid.
45. M/1-3 ectocingulid.
46. M/1 trigonid/talonid relative width.
47. M/2 trigonid/talonid relative width.
48. M/3 trigonid/talonid relative width.
49. M/3 development.
50. **M/3 root(s)**
51. Extension of mandibular symphysis.
52. Fusion of mandibular symphysis.
53. **Anteriormost mental foramen**
54. **Posteriormost mental foramen**
55. Horizontal ramus (body).
56. Position of the coronoid apophysis.
57. Orientation of the coronoid apophysis.
58. Development of the coronoid apophysis.
59. Articular condyle.
60. **Mandibular condyle shape**
61. Coronoid foramen.
62. Angular process.
63. Anterior coronoid fossa.
64. Number of upper incisors.
65. Relative size of upper incisors.
66. Development of I1/.
67. Development of I2/.
68. Development **of I3/.**
69. Upper anterior diastema.
70. Development of C1/.
71. C1/ root(s).
72. P1/ presence.
73. P1/ root(s).
74. P2/ presence.
75. P2/ root(s).
76. P2/ protocone.
77. P3/ root(s).
78. P3/ protocone.
79. P3/ metacone.
80. P3/ conules.
81. P4/ conules.
82. P4/ root(s).
83. P4/ metacone development.
84. P4/ metacone relations.
85. P4/ hypocone.
86. P4/ loph.
87. P4/ (and P3/ when protocone is developed) postprotocrista.
88. Upper molar bunodonty.
89. Upper molar lophodonty.
90. M1-3/ lophs.
91. M1-3/ parastyle position.
92. M1-2/ parastyle development.
93. M1-3/ ectocingulum.
94. **M1-2/ Stylar shelf development**
95. M1-3/ pericone.
96. M1-3/ Pre- and postcingulum.
97. **M1-3/ Hypocone**
98. M1-3/ mesostyle development.
99. M1-3/ mesostyle position.
100. M1-3/ centrocrista development.
101. M1-3/ centrocrista shape.
102. M1-3/ postmetacrista.
103. M1-3/ preparacrista.
104. M3/ metacone.
105. M1-3/ conules.
106. M1-3/ postprotocrista.
107. M1-3/ prehypocrista.
108. M1-3/ interloph.
109. M1-2/ distocrista.
110. M1-3/ postentoconule.
111. M1/ and M2/ relative size.
112. M2/ and M3/ relative size.
113. M1-2/ lingual root(s).
114. M3/ protocone root.
115. M3/ hypocone root.
116. M1-3/ crown height.
117. M1-3/ occlusal outline.
118. Pneumatisation of skull bones.
119. **Rostrum elongation.**
120. **Rostrum relative width to length - ratio of width of the snout at canine level to preorbital length.**
121. Extension of upper tooth row.
122. **Palatine development.**
123. **Major palatine foramen.**
124. **Location of the choanae.**
125. **Postpalatine torus.**
126. **Nasal cavity.**
127. Nasal development.
128. Nasal fossa.
129. **Frontal and maxillary contact**
130. Premaxillary-frontal contact.
131. **Lacrimal presence**
132. **Lacrimal facial process**
133. Lacrimal tubercle.
134. **Lacrimal foramen.**
135. Orbit location.
136. Maxillary/jugal relative orbital development.
137. Infraorbital foramen size.
138. **Infraorbital foramen position *vs* teeth**
139. Infraorbital foramen position *vs* orbit (= orbit position)
140. Submaxillary fossa.
141. **Frontal and parietal relative size**
142. Tuber maxillae development.
143. Tuber maxillae position.
144. Ascending process of the palatine.
145. Morphology of the zygomatic arch.
146. Jugal distal extension.
147. Postorbital constriction.
148. Postorbital process of the frontal.
149. Postorbital process of the jugal.
150. Zygomatic arch shape.

151.Cerebral part of squamosal.
152.Zygomatic process of the squamosal.
153.Parietal-alisphenoid contact.
154.**Parietal anterior extent**
155.**Alisphenoid canal**
156. **Foramen rotundum and sphenorbital fissure confluence**
157.External auditory meatus.
158.Post-tympanic process.
159.Sagittal crest.
160.Nuchal crests.
161.Periotic mastoidy.
162.Periotic pars mastoidea.
163.**Periotic: Fenestra vestibuli size**
164.**Periotic: Fenestra vestibuli shape**
165.**Periotic : Fenestra cochleae**
166.Periotic pars cochlearis.
167.**Periotic : Subarcuata fossa**
168.Postglenoid foramen.
169.**Tegmen tympani**
170.**Jugular foramen**
171.**basioccipital midline ventral keel**
172.Hypoglossal foramen.
173.Ethmoidal foramen
174.**Inner ear – Cochlea secondary bony spiral lamina**
175.**Inner ear – Cochlea number of turns**

Taxa analyzed

We compared *Ocepeia daouiensis* with paenungulates, other afrotherians, condylarths, perissodactyles, and insectivorous-like primitive eutherians to assess its ordinal and supra-ordinal relationships. Comparison included primitive representatives (*i.e.*, representatives of primitive ancestral morphotypes) of paenungulate and "altungulate" orders (=lophodont ungulates). We included the primitive embrithopod *Namatherium* in our comparisons [11]. The outgroup corresponds to the generalized eutherian morphotype, represented by leptictids and cimolestids, as well as to other basalmost eutherians such as *Eomaia*. With respect to *Eritherium* study [1], the outgroup comparison is extended to the Zhelestidae (petrosal: [28]), *Protungulatum* (petrosal : [23]), *Hyopsodus*, *Todralestes variabilis*, *Potamogale* (Tenrecidae), *Ptolemaia lyonsi* and *Ptolemaia grangeri* (Ptolemaiida), *Orycteropus afer* (Tubulidentata) in order to test the basal relationships of *Ocepeia* and paenungulates among other placentals, and the hypotheses of Afrotheria *versus* Laurasiatheria.

List of taxa compared (*added to Matrix of Gheerbrant [1])

1. Eutheria [Euth in matrix]: *Maelestes*, *Cimolestes*, *Asioryctes*, leptictids, *Acristatherium*
2. *Zhelestidae (Eutheria, ?Placentalia): *Parazhelestes*, *Eozhelestes*, *Zhelestes*, *Aspanlestes*
3. *Protungulatum [Prot]
4. Arctocyonidae [Arc] : *Loxolophus*, *Tricentes*, *Lambertocyon*, *Artocyon*
5. *Ocepeia daouiensis* [Oce]
6. Louisinidae [Teil]: *Teilhardimys* (=*Microhyus*)
7. Phenacodonta [Phena] : *Ectocion*, *Phenacodus*
8. Perissodactyla [Peris]: primitive genera *Hyracotherium*, *Sifrhippus*, *Cymbalophus*, *Pachynolophus*
9. *Radinskya* [Rad]
10. **Hyopsodus* [Hyop]
11. **Todralestes variabilis* [Todr]
12. * Ptolemaiida [Ptol]: *Ptolemaia grangeri*, *P. lyonsi*
13. *Tenrecoidea [Pto]: *Potamogale*
14. *Tubulidentata [Tub]: *Orycteropus*
15. Macroscelidea [Macro]: primitive taxa (*Chambius*, *Herodotius*, ?*Metoldobotes*) and extant taxa (*Petrodromus*, *Rhynchocyon*)
16. Hyracoidea [Hyra]: primitive genera *Seggeurius*, *Microhyrax*, and Pliohyracidae
17. Anthracobunidae [Anth]: *A. pinfoldi*, *Indobune*
18. Desmostylia [Desm]: *Behemotops* and others
19. Sirenia [S i r]: primitive genera *Prorastomus*, *Pezosiren*, *Protosiren*, *Eosiren*, *Eotheroides*, *Prototherium*
20. Phenacolophids [P h e]: *Phenacolophus*
21. *Minchenella* [Min]:
22. Embrithopoda [Emb]: *Namatherium*, *Palaeoamasiia*, *Crivadiatherium*, *Arsinoitherium*
23. *Eritherium* [Erit]
24. *Phosphatherium* [Phos]
25. *Numidotherium* [Num]

Part III.
TNT analysis, method, cladograms, diagnose of nodes

The parsimony analysis was developed by means of the TNT program [29]. We used the "traditional search" command for all analyses. Bremer indices were calculated for 10000 trees with additional 10 steps longer than in the shortest obtained tree. The 18 uninformative characters (3, 5, 15, 20, 56, 57, 58, 64, 68, 85, 95, 129, 131, 142, 154, 166, 173, 174, 175) were made inactive before the analysis.
The interface WINCLADA associated with the heuristic algorithm NONA was used in complement of our study, especially for the revision of the matrix, for the preliminary explorative analysis of tree topology and for examination of character distribution in trees.

1. Analysis with all characters unordered

1.1 Unweighted analysis

1 tree found

Cladogram 1

Bremer supports (from 10000 trees, cut 0)

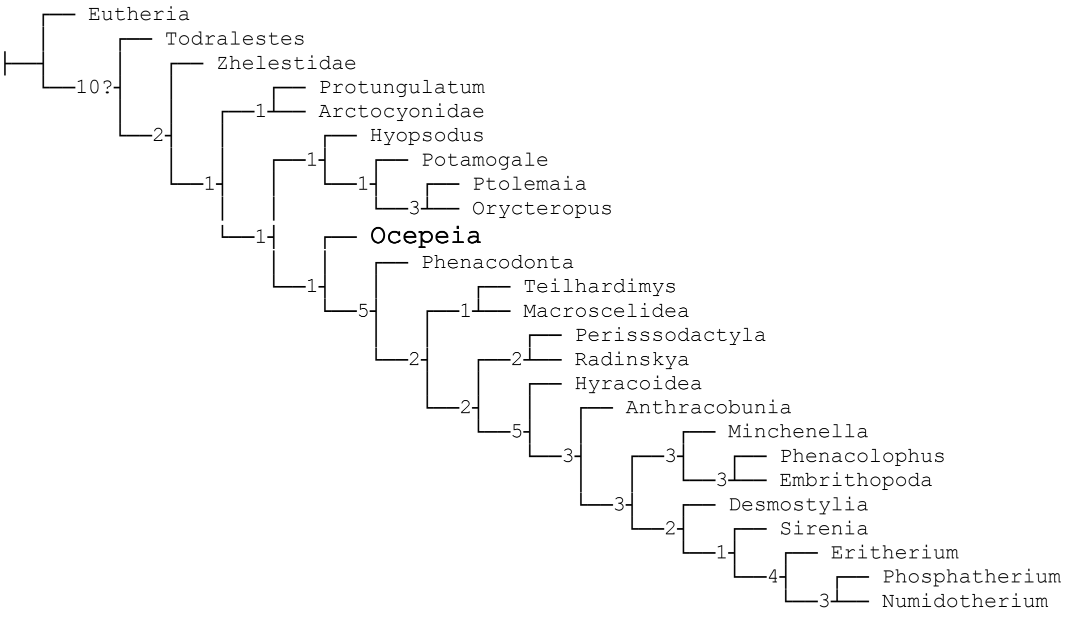

Tree length: 596. Retention index: 53.6. Consistency Index: 40.8

1.2 Implied weighting (standard) analysis

Cladogram 2

1 tree found.

```
    ┌──0 Eutheria
    │   ┌──10 Todralestes
├─25┤   │   ┌──1 Zhelestidae
    └─27┤   │       ┌──11 Ptolemaia
        └─26┤   ┌─39┴──13 Orycteropus
            │   │   ┌──3 Arctocyonidae
            └─30┤   │   ┌──2 Protungulatum
                └─29┤   │   ┌──9 Hyopsodus
                    └─28┤   │       ┌──4 Ocepeia
                        └─33┤   ┌─31┴──12 Potamogale
                            │   │   ┌──6 Phenacodonta
                            └─32┤   │       ┌──5 Teilhardimys
                                └─36┤   ┌─34┴──14 Macroscelidea
                                    │   │       ┌──7 Perisssodactyla
                                    └─35┤   ┌─37┴──8 Radinskya
                                        │   │   ┌──20 Minchenella
                                        └─38┤   │   ┌──15 Hyracoidea
                                            └─41┤   │   ┌──16 Anthracobunia
                                                └─40┤   │   ┌──17 Desmostylia
                                                    └─42┤   │       ┌──19 Phenacolophus
                                                        └─43┤   ┌─47┴──21 Embrithopoda
                                                            └─46┤   ┌──22 Eritherium
                                                                └─45┤   ┌──18 Sirenia
                                                                    └─44┤   ┌──23 Phosphatherium
                                                                        └─48┴──24 Numidotherium
```

Tree lengths: 596.

2. Analysis with characters ordered

152 are characters ordered.

2.1 Unweighted analysis

Cladogram 3

Traditional search, 2 trees found, score 665. Strict consensus of 2 trees.

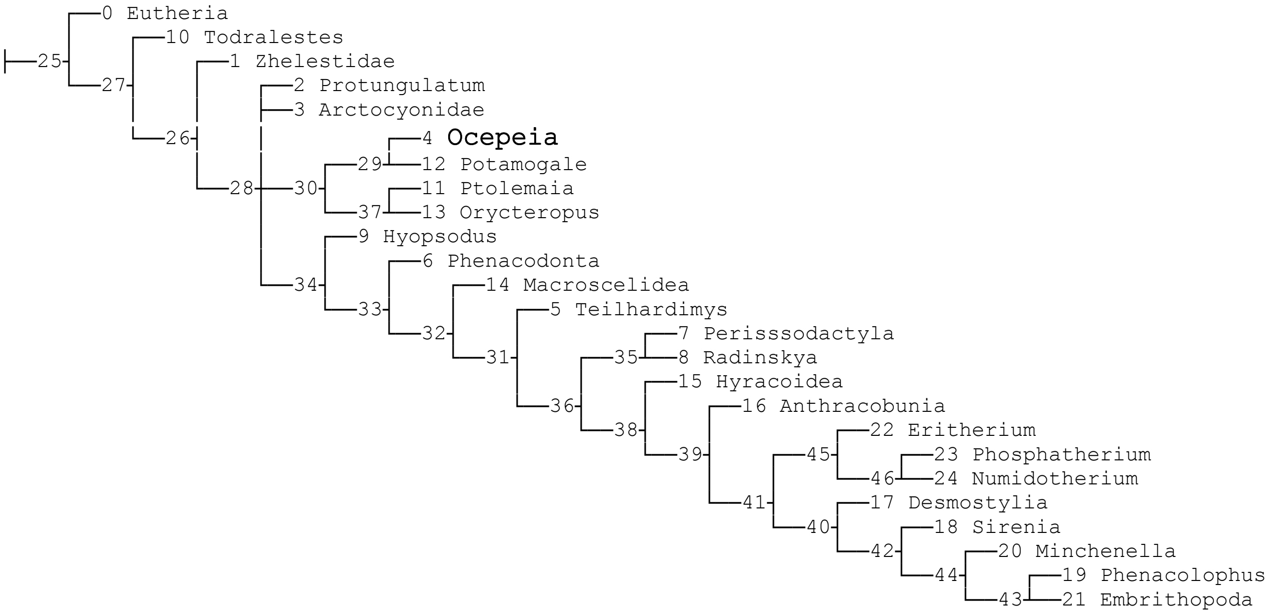

Tree lengths: 665. Retention index: 54.6. Consistency Index: 37

Bremer supports (from 10000 trees, cut 0)

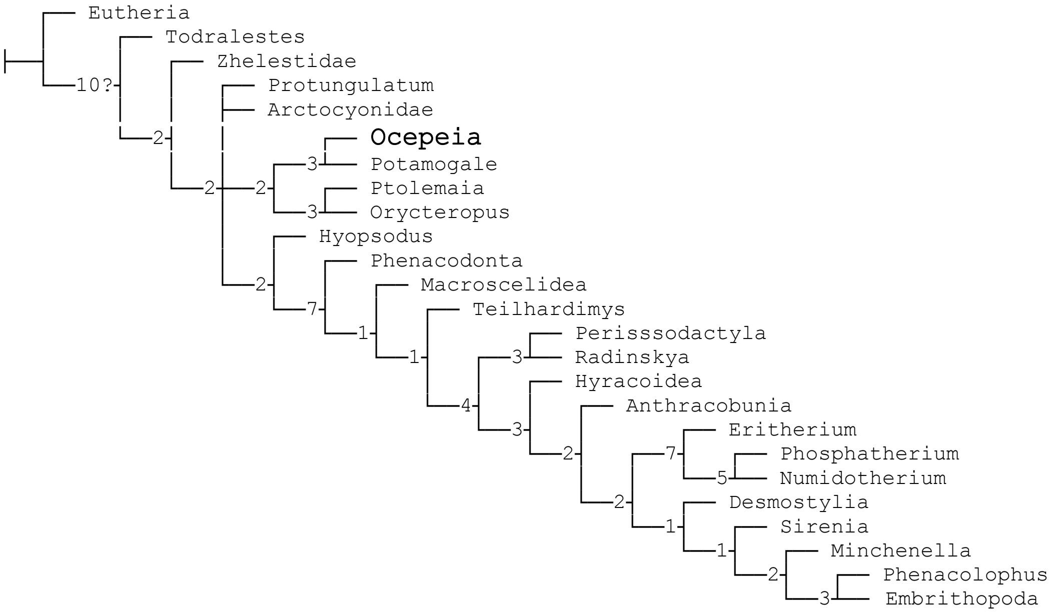

Synapomorphies common to 2 trees
(Node numbers refer to nodes in consensus)

Important note: For correspondence with character description (Part I) and character summary list (Part II), number *n* of characters ***should be read as n+1*** because TNT starts numbering characters from 0.

Eutheria :
All trees:
No autapomorphies:

Zhelestidae :
All trees:
Char. 47: 0 --> 1
Char. 50: 0 --> 1
Char. 68: 0 --> 1
Char. 82: 0 --> 1
Char. 83: 0 --> 1
Char. 136: 0 --> 1
Some trees:
Char. 21: 0 --> 1

Protungulatum :
All trees:
Char. 27: 0 --> 1
Char. 61: 1 --> 2
Char. 143: 0 --> 2
Some trees:
Char. 35: 0 --> 1
Char. 92: 0 --> 1

Arctocyonidae :
All trees:
Char. 30: 0 --> 1
Some trees:
Char. 131: 0 --> 1
Char. 155: 0 --> 1

Ocepeia :
All trees:
Char. 20: 0 --> 1
Char. 22: 1 --> 0
Char. 30: 0 --> 1
Char. 31: 0 --> 2
Char. 33: 0 --> 1
Char. 36: 0 --> 1
Char. 37: 0 --> 1
Char. 47: 0 --> 1
Char. 51: 0 --> 1
Char. 52: 01 --> 2
Char. 62: 0 --> 1
Char. 66: 0 --> 1
Char. 68: 0 --> 1
Char. 73: 0 --> 1
Char. 97: 0 --> 1
Char. 98: 0 --> 2
Char. 100: 0 --> 2
Char. 111: 1 --> 0
Char. 117: 0 --> 2
Char. 119: 1 --> 3
Char. 125: 0 --> 1
Char. 160: 0 --> 1
Char. 168: 0 --> 1
Char. 170: 1 --> 2
Some trees:
Char. 167: 1 --> 0

Teilhardimys :
All trees:
Char. 16: 0 --> 1
Char. 20: 0 --> 1
Char. 22: 1 --> 0
Char. 30: 0 --> 1
Char. 53: 2 --> 3
Char. 74: 0 --> 1
Char. 75: 0 --> 2
Char. 112: 1 --> 0
Some trees:
Char. 31: 2 --> 0

Phenacodonta :
All trees:
Char. 10: 1 --> 0
Char. 37: 0 --> 1
Char. 78: 1 --> 2
Char. 83: 1 --> 2
Char. 97: 0 --> 1
Char. 98: 0 --> 1
Char. 100: 0 --> 1
Char. 110: 1 --> 0
Char. 131: 0 --> 1

Perisssodactyla :
All trees:
Char. 83: 1 --> 2
Char. 91: 1 --> 0
Char. 104: 12 --> 0

Radinskya :
All trees:
Char. 104: 12 --> 3
Char. 112: 1 --> 2
Char. 124: 1 --> 0

Hyopsodus :
All trees:
Char. 5: 0 --> 1
Char. 7: 0 --> 1
Char. 10: 1 --> 2
Char. 12: 0 --> 1
Char. 28: 0 --> 1
Char. 65: 0 --> 1
Char. 69: 0 --> 1
Char. 136: 0 --> 1
Char. 146: 0 --> 1
Char. 150: 0 --> 1
Char. 151: 0 --> 1
Char. 158: 0 --> 1
Char. 162: 0 --> 1
Some trees:
Char. 129: 0 --> 1

Todralestes :
All trees:
Char. 9: 0 --> 1
Char. 18: 0 --> 1
Char. 52: 01 --> 2
Char. 96: 0 --> 1
Char. 104: 0 --> 1

Ptolemaia :
All trees:
Char. 58: 1 --> 0
Char. 61: 1 --> 0
Char. 110: 1 --> 2
Char. 111: 1 --> 2
Char. 136: 0 --> 1
Some trees:
Char. 45: 0 --> 1

Potamogale :
All trees:
Char. 5: 0 --> 1
Char. 7: 0 --> 1
Char. 38: 1 --> 0
Char. 45: 01 --> 2
Char. 58: 1 --> 0
Char. 61: 1 --> 0
Char. 69: 0 --> 1
Char. 70: 1 --> 0
Char. 87: 1 --> 0
Char. 93: 1 --> 0
Char. 99: 0 --> 1
Char. 101: 1 --> 0
Char. 103: 0 --> 1
Char. 124: 0 --> 1
Char. 134: 1 --> 0
Char. 135: 1 --> 2
Char. 136: 0 --> 1
Char. 138: 0 --> 2
Char. 146: 0 --> 1
Char. 154: 0 --> 1
Char. 159: 0 --> 1

Orycteropus :
All trees:
Char. 7: 0 --> 3
Char. 9: 0 --> 2
Char. 58: 1 --> 2
Char. 69: 0 --> 2
Char. 73: 0 --> 1
Char. 76: 1 --> 2
Char. 81: 1 --> 0
Char. 116: 0 --> 1
Char. 118: 0 --> 1
Char. 120: 0 --> 1
Char. 125: 0 --> 1
Char. 133: 1 --> 0
Char. 134: 1 --> 0
Char. 137: 1 --> 2
Char. 146: 0 --> 1
Char. 158: 0 --> 1

Macroscelidea :
All trees:
Char. 7: 0 --> 1
Char. 17: 0 --> 1
Char. 31: 02 --> 1
Char. 50: 0 --> 1
Char. 65: 0 --> 2
Char. 70: 1 --> 0
Char. 79: 0 --> 1
Char. 123: 0 --> 1
Char. 134: 1 --> 0
Char. 146: 0 --> 1
Char. 148: 0 --> 1
Char. 150: 0 --> 1
Char. 168: 0 --> 1
Some trees:
Char. 35: 1 --> 0
Char. 132: 0 --> 1

Hyracoidea :
All trees:
Char. 8: 1 --> 0
Char. 37: 0 --> 1
Char. 39: 12 --> 0
Char. 53: 2 --> 1
Char. 62: 0 --> 1
Char. 65: 0 --> 2
Char. 74: 0 --> 1
Char. 75: 0 --> 2
Char. 93: 2 --> 1
Char. 97: 0 --> 1
Char. 98: 0 --> 2
Char. 100: 01 --> 2
Char. 102: 1 --> 0
Char. 112: 1 --> 2
Char. 148: 0 --> 2

Anthracobunia :
All trees:
Char. 38: 1 --> 0
Char. 47: 0 --> 12
Char. 79: 0 --> 1
Char. 104: 2 --> 3
Char. 134: 2 --> 0

Desmostylia :
All trees:
Char. 47: 0 --> 2
Char. 53: 2 --> 1
Char. 58: 2 --> 1
Char. 122: 1 --> 2
Char. 123: 0 --> 1

Sirenia :
All trees:
Char. 8: 1 --> 0

Char. 12: 0 --> 2
Char. 13: 0 --> 1
Char. 44: 1 --> 0
Char. 70: 1 --> 0
Char. 76: 1 --> 0
Char. 81: 1 --> 0
Char. 121: 0 --> 2
Char. 127: 1 --> 2
Char. 134: 2 --> 3
Char. 138: 1 --> 2
Char. 139: 0 --> 1
Char. 159: 0 --> 1
Char. 170: 1 --> 0

Phenacolophus :
All trees:
Char. 1: 1 --> 0
Char. 18: 1 --> 0
Char. 29: 1 --> 0
Char. 35: 1 --> 0
Char. 47: 0 --> 12
Char. 110: 1 --> 0
Char. 134: 2 --> 1

Minchenella :
All trees:
Char. 16: 0 --> 1
Char. 45: 0 --> 2
Char. 46: 1 --> 2
Char. 106: 1 --> 2
Char. 114: 1 --> 0

Embrithopoda :
All trees:
Char. 3: 0 --> 1
Char. 26: 0 --> 1
Char. 31: 2 --> 1
Char. 34: 1 --> 2
Char. 62: 0 --> 1
Char. 77: 01 --> 2
Char. 93: 12 --> 0
Char. 100: 2 --> 3
Char. 102: 1 --> 0
Char. 104: 0 --> 2

Eritherium :
All trees:
Char. 31: 0 --> 1
Char. 42: 01 --> 2
Char. 46: 1 --> 2
Char. 48: 2 --> 1

Phosphatherium :
All trees:
Char. 58: 2 --> 1
Char. 119: 0 --> 2
Char. 170: 2 --> 1

Numidotherium :
All trees:
Char. 7: 2 --> 3
Char. 9: 0 --> 2
Char. 43: 1 --> 0
Char. 52: 01 --> 3
Char. 54: 0 --> 1
Char. 68: 0 --> 2
Char. 71: 0 --> 1
Char. 74: 0 --> 1
Char. 75: 0 --> 1
Char. 99: 0 --> 1
Char. 112: 1 --> 2
Char. 114: 1 --> 2
Char. 117: 0 --> 1
Char. 120: 0 --> 1
Char. 123: 0 --> 1
Char. 126: 0 --> 1
Char. 135: 1 --> 2
Char. 138: 0 --> 1
Char. 148: 0 --> 1
Char. 150: 0 --> 1
Char. 162: 0 --> 1
Char. 171: 0 --> 1

Node 26 :
All trees:
Char. 40: 0 --> 1
Char. 90: 0 --> 1
Char. 110: 0 --> 1
Char. 111: 0 --> 1

Node 27 :
All trees:
No synapomorphies

Node 28 :
All trees:
Char. 76: 0 --> 1
Char. 101: 0 --> 1
Char. 174: 0 --> 2

Node 29 :
All trees:
Char. 50: 0 --> 2
Char. 53: 2 --> 3
Char. 64: 0 --> 1
Char. 90: 1 --> 0
Char. 92: 0 --> 1
Char. 93: 2 --> 1
Char. 135: 0 --> 1
Char. 147: 0 --> 1
Char. 170: 0 --> 1
Some trees:
Char. 102: 1 --> 0

Node 30 :
All trees:
Char. 10: 1 --> 3
Char. 65: 0 --> 1
Char. 71: 0 --> 1
Char. 77: 0 --> 2
Char. 104: 0 --> 12
Char. 119: 0 --> 1
Char. 144: 0 --> 1

Node 31 :
All trees:
Char. 25: 0 --> 12
Char. 29: 0 --> 1
Char. 33: 0 --> 1
Char. 40: 1 --> 0
Char. 77: 1 --> 2
Char. 105: 0 --> 1

Node 32 :
All trees:
Char. 18: 0 --> 1
Char. 89: 0 --> 1
Char. 104: 0 --> 1
Char. 116: 0 --> 1
Char. 140: 1 --> 0
Some trees:
Char. 163: 0 --> 1

Node 33 :
All trees:
Char. 48: 0 --> 1
Char. 77: 0 --> 1
Char. 78: 0 --> 1
Char. 80: 0 --> 1
Char. 82: 0 --> 2
Char. 83: 0 --> 1
Char. 107: 0 --> 1
Char. 112: 0 --> 1
Char. 121: 0 --> 2
Char. 124: 0 --> 1
Some trees:
Char. 58: 1 --> 2
Char. 92: 0 --> 1

Node 34 :
All trees:
Char. 39: 0 --> 1
Char. 96: 0 --> 2
Some trees:
Char. 21: 0 --> 1
Char. 31: 1 --> 2
Char. 133: 1 --> 2
Char. 154: 0 --> 1
Char. 170: 0 --> 2

Node 35 :
All trees:
Char. 88: 1 --> 2
Char. 108: 0 --> 1
Char. 116: 1 --> 0
Char. 170: 2 --> 0

Node 36 :
All trees:
Char. 10: 1 --> 0
Char. 15: 0 --> 1
Char. 24: 1 --> 2
Char. 34: 0 --> 1
Char. 41: 1 --> 2
Char. 48: 1 --> 2
Char. 51: 0 --> 1
Char. 89: 1 --> 2
Char. 113: 0 --> 1

Node 37 :
All trees:
Char. 26: 0 --> 2
Char. 48: 0 --> 1
Char. 49: 0 --> 2
Char. 115: 0 --> 2
Some trees:
Char. 140: 1 --> 0

Node 38: P**aenungulata**
All trees:
Char. 27: 0 --> 1
Char. 36: 1 --> 0
Char. 60: 0 --> 1
Char. 114: 0 --> 1
Char. 121: 2 --> 0
Char. 134: 1 --> 2
Char. 160: 0 --> 1
Some trees:
Char. 129: 0 --> 1

Node 39 :
All trees:
Char. 39: 12 --> 3
Char. 41: 2 --> 3
Char. 43: 0 --> 12
Char. 72: 0 --> 1
Char. 86: 0 --> 1
Char. 106: 2 --> 1
Char. 109: 0 --> 1

Node 40 :
All trees:
Char. 54: 0 --> 1
Char. 61: 1 --> 2
Char. 77: 2 --> 1
Char. 104: 2 --> 0
Char. 138: 0 --> 1

Node 41 :
All trees:
Char. 0: 0 --> 1
Char. 10: 0 --> 1
Char. 31: 2 --> 0
Char. 82: 2 --> 01
Char. 102: 1 --> 2

Node 42 :
All trees:
Char. 126: 0 --> 1
Char. 127: 0 --> 1
Char. 170: 2 --> 1

Node 43 :
All trees:
Char. 24: 2 --> 3
Char. 32: 0 --> 1
Char. 43: 12 --> 0
Char. 97: 0 --> 1
Char. 98: 0 --> 2
Char. 100: 01 --> 2

Node 44 :
All trees:
Char. 25: 2 --> 1
Char. 28: 1 --> 0
Char. 31: 0 --> 2
Char. 38: 1 --> 0
Char. 40: 0 --> 1
Char. 91: 1 --> 0
Char. 101: 1 --> 0
Char. 102: 2 --> 1
Char. 109: 1 --> 0

Node 45 :
All trees:
Char. 3: 0 --> 1
Char. 6: 0 --> 1
Char. 7: 01 --> 2

Char. 10: 1 --> 2
Char. 12: 0 --> 1
Char. 16: 0 --> 1
Char. 20: 0 --> 1
Char. 40: 0 --> 2
Char. 62: 0 --> 12
Char. 135: 0 --> 1

Node 46 :
All trees:
Char. 3: 1 --> 2
Char. 6: 1 --> 2
Char. 10: 2 --> 3
Char. 24: 2 --> 3
Char. 32: 0 --> 1
Char. 34: 1 --> 2
Char. 35: 1 --> 0
Char. 46: 1 --> 0
Char. 50: 0 --> 2
Char. 85: 0 --> 1
Char. 88: 1 --> 3
Char. 92: 1 --> 0
Char. 93: 2 --> 3
Char. 106: 1 --> 3
Char. 134: 2 --> 3
Char. 139: 0 --> 1

2.2 Implied weighting (standard) analysis

Cladogram 4

```
1 trees retained.

Tree 0:
```

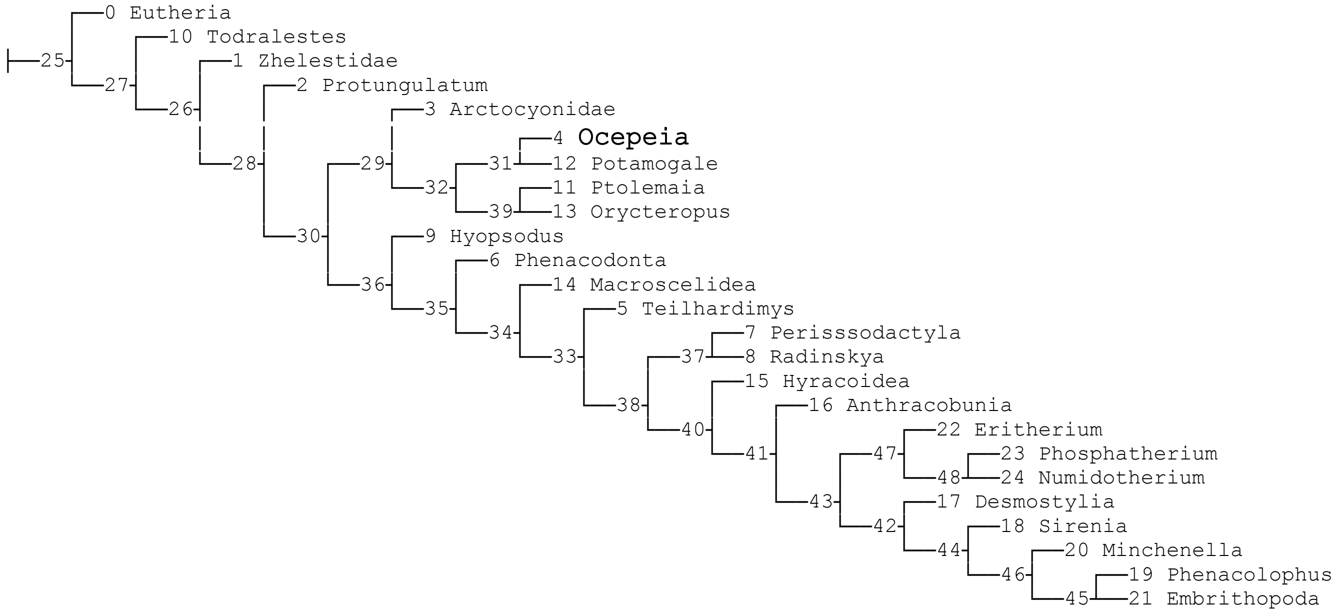

Tree length: 666. Retention index: 54.6; Consistency Index: 37

2.3 Unweighted and partioned analysis : dental characters

Cladogram 5

```
1 trees retained.

Tree 0:
```

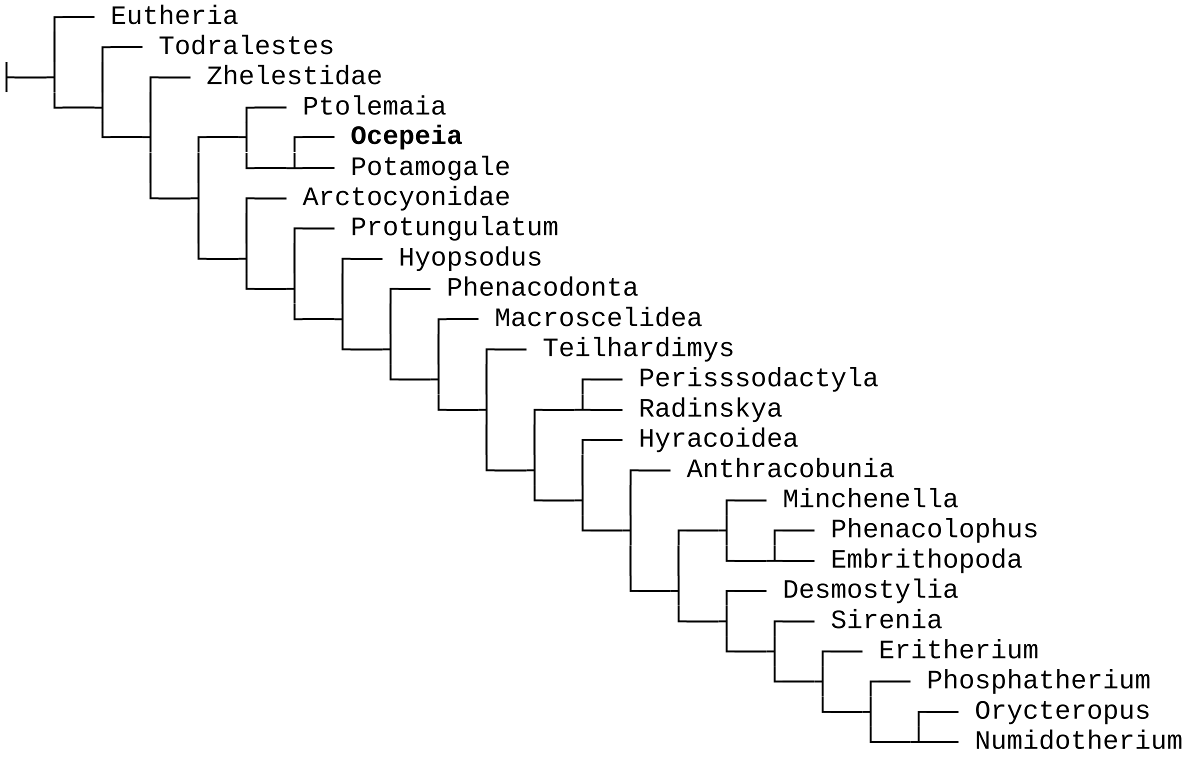

Tree length: 422. Retention index: 59.8; Consistency Index: 36.5

2.4 Implied weighting and partioned analysis : dental characters

Cladogram 6

```
2 trees retained.

Strict consensus of 2 trees
```

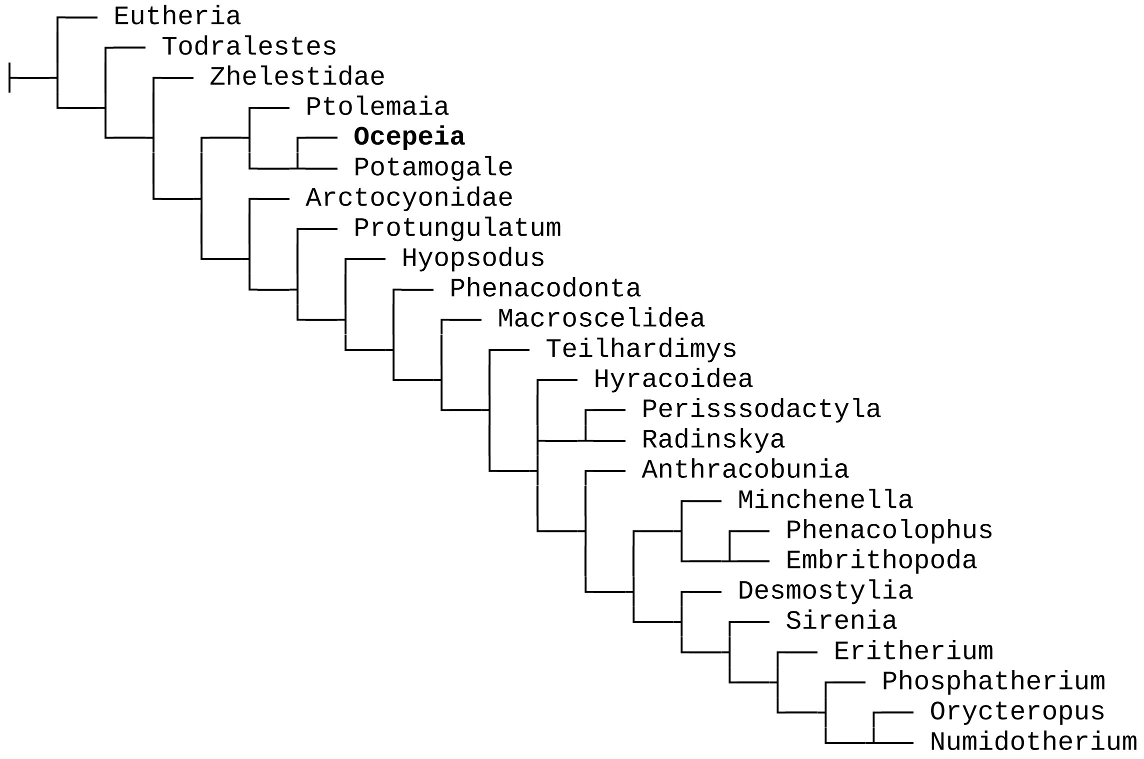

2.5 Unweighted and partioned analysis : Skull characters

Cladogram 7 - All taxa

10 trees retained. Strict consensus of 10 trees (0 taxa excluded)

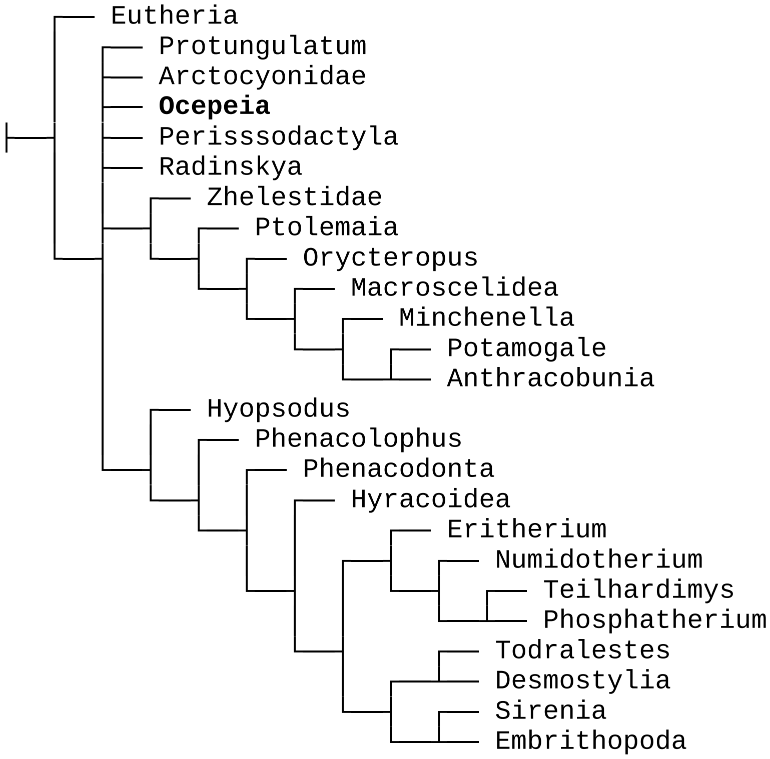

Tree length: 168. Retention index: 51.8; Consistency Index: 44.6

Cladogram 8 - Only taxa with well known skull characters

10 trees retained. Strict consensus of 10 trees (7 taxa excluded: Protungulatum, Protungulatum, Teilhardimys, Todralestes, Ptolemaia, Anthracobunia, Phenacolophus, Minchenella)

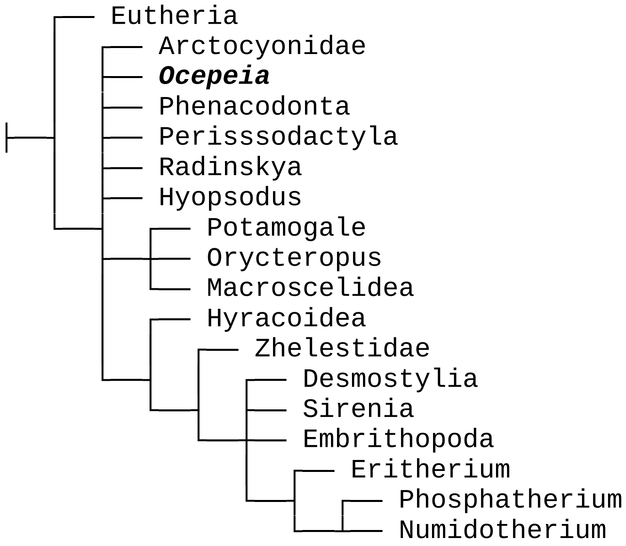

3. Analysis with character ordered + step matrix

Character tranformations that are considered unlikely are coded with an additional cost of 10 steps. This concerns for instance reversal of the loss of teeth. Cost between 1 and 10 corresponds to ordered characters.

Important note: For correspondence with character description (Part I) and character summary list (Part II), number *n* of characters ***should be read as n+1*** because TNT starts numbering characters from 0.

Character step matrix with costs for each state is below.

```
Char. 2:
        0>1  1
        1>0  10
Char. 4:
        0>1  1
        1>0  10
Char. 6:
        0/1  1     0>2  1
        1/0  1     1>2  1
        2>0  10    2>1  10
Char. 10:
        0/1  1     0/2  1     0>3  1
        1/0  1     1/2  1     1>3  1
        2/0  1     2/1  1     2>3  1
        3>0  10    3>1  10    3>2 10
Char. 12:
        0/1  1     0/2  1     0>3  1
        1/0  1     1/2  1     1>3  1
        2/0  1     2/1  1     2>3  1
        3>0  10    3>1  10    3>2 10
Char. 26:
        0>1  1     0>2  1
        1>0  10    1/2  1
        2>0  10    2/1  1
Char. 51:
        0>1  1
        1>0  10
Char. 63:
        0>1  1     0>2  2     0>3  3
        1>0  10    1>2  1     1>3  2
        2>0  10    2>1  10    2>3  1
        3>0  10    3>1  10    3>2 10
Char. 67:
        0/1  1     0/2  1
        1/0  1     1/2  2
        2/0  1     2/1  2
Char. 69:
        0/1  1     0>2  1
        1/0  1     1>2  1
        2>0  10    2>1  10
Char. 71:
        0>1  1
        1>0  10
Char. 73:
        0>1  1
        1>0  10
Char. 88:
        0/1  1     0/2  1     0/3  2
        1/0  1     1/2  1     1/3  2
        2/0  1     2/1  1     2/3  1
        3/0  2     3/1  2     3/2  1
Char. 89:
        0>1  1     0>2  1
        1>0  10    1/2  1
        2>0  10    2/1  1
Char. 98:
        0>1  1     0>2  1
        1>0  10    1/2  1
        2>0  10    2/1  1
Char. 104:
        0/1  1     0/2  2     0/3  1
        1/0  1     1/2  1     1/3  2
        2/0  2     2/1  1     2/3  3
        3/0  1     3/1  2     3/2  3
Char. 106:
        0/1  1     0/2  1     0/3  2
        1/0  1     1/2  1     1/3  2
        2/0  1     2/1  1     2/3  1
        3/0  2     3/1  2     3/2  1
Char. 110:
        0/1  1     0/2  1
        1/0  1     1/2  2
        2/0  1     2/1  2
Char. 111:
        0/1  1     0/2  2     0/3  1
        1/0  1     1/2  1     1/3  2
        2/0  2     2/1  1     2/3  3
        3/0  1     3/1  2     3/2  3
Char. 115:
        0>1  1     0>2  1
        1>0  10    1/2  1
        2>0  10    2/1  1
Char. 118:
        0/1  1     0/2  1
        1/0  1     1/2  2
        2/0  1     2/1  2
Char. 119:
        0/1  1     0/2  1     0/3  2
        1/0  1     1/2  2     1/3  3
        2/0  1     2/1  2     2/3  1
        3/0  2     3/1  3     3/2  1
Char. 121:
        0/1  1     0/2  1
        1/0  1     1/2  2
        2/0  1     2/1  2
Char. 123:
        0/1  1     0/2  1
```

```
          1/0   1     1/2   2
          2/0   1     2/1   2
Char. 130:
          0>1   1
          1>0   10
Char. 133:
          0/1   1     0/2   2     0/3   1
          1/0   1     1/2   1     1/3   2
          2/0   2     2/1   1     2/3   3
          3/0   1     3/1   2     3/2   3
Char. 134:
          0/1   1     0/2   1     0/3   2
          1/0   1     1/2   2     1/3   3
          2/0   1     2/1   2     2/3   1
          3/0   2     3/1   3     3/2   1
Char. 137:
          0/1   1     0/2   1
          1/0   1     1/2   2
          2/0   1     2/1   2
Char. 148:
          0/1   1     0/2   1
          1/0   1     1/2   2
          2/0   1     2/1   2
Char. 167:
          0/1   1     0/2   1
          1/0   1     1/2   2
          2/0   1     2/1   2
Char. 174:
          0>1   1     0>2   1
          1>0   10    1>2   1
          2>0   10    2>1   10
```

3.1 Unweighted analysis

Cladogram 9

8 trees retained. Strict consensus of 8 trees with Bremer support (ital.) above node number

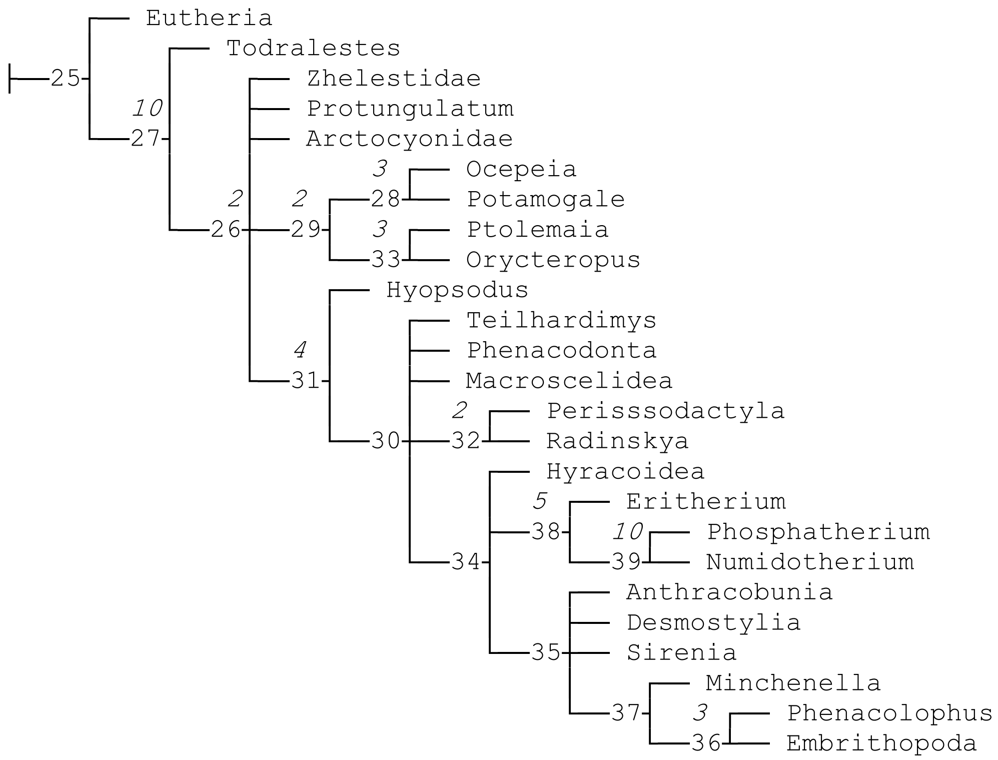

Tree length: 666. Retention index: 54.4. Consistency Index: 36.8.

Synapomorphies of Cladogram 9, consensus of 8 trees
(Node numbers refer to nodes in consensus)

Important note: For correspondence with character description (Part I) and character summary list (Part II), number *n* of characters ***should be read as n+1*** because TNT starts numbering characters from 0.

Eutheria :
All trees:
No autapomorphies:

Zhelestidae :
All trees:
Char. 47: 0 --> 1
Char. 50: 0 --> 1
Char. 68: 0 --> 1
Some trees:
Char. 11: 1 --> 0
Char. 21: 0 --> 1
Char. 45: 0 --> 1
Char. 76: 1 --> 0
Char. 82: 0 --> 1
Char. 83: 0 --> 1
Char. 136: 0 --> 1

Protungulatum :
All trees:
Char. 27: 0 --> 1
Char. 61: 1 --> 2
Char. 143: 0 --> 2
Some trees:
Char. 10: 0 --> 1
Char. 35: 0 --> 1
Char. 92: 0 --> 1

Arctocyonidae :
All trees:
Char. 30: 0 --> 1
Some trees:
Char. 131: 0 --> 1
Char. 155: 0 --> 1

Ocepeia :
All trees:
Char. 6: 0 --> 1
Char. 9: 0 --> 1
Char. 12: 0 --> 3
Char. 20: 0 --> 1
Char. 22: 1 --> 0
Char. 30: 0 --> 1
Char. 31: 0 --> 2
Char. 33: 0 --> 1
Char. 36: 0 --> 1
Char. 37: 0 --> 1
Char. 47: 0 --> 1
Char. 51: 0 --> 1
Char. 52: 01 --> 2
Char. 62: 0 --> 1
Char. 66: 0 --> 1
Char. 68: 0 --> 1
Char. 73: 0 --> 1
Char. 97: 0 --> 1
Char. 98: 0 --> 2
Char. 100: 0 --> 2
Char. 111: 1 --> 0
Char. 117: 0 --> 2
Char. 119: 01 --> 3
Char. 121: 0 --> 1
Char. 125: 0 --> 1
Char. 133: 1 --> 2
Char. 160: 0 --> 1
Char. 168: 0 --> 1
Char. 170: 1 --> 2
Some trees:
Char. 150: 1 --> 0

Teilhardimys :
All trees:
Char. 16: 0 --> 1
Char. 20: 0 --> 1
Char. 22: 1 --> 0
Char. 30: 0 --> 1
Char. 53: 2 --> 3
Char. 74: 0 --> 1
Char. 75: 0 --> 2
Some trees:
Char. 21: 1 --> 0
Char. 25: 01 --> 2
Char. 26: 0 --> 1
Char. 28: 0 --> 1
Char. 29: 0 --> 1
Char. 31: 2 --> 0
Char. 33: 0 --> 1
Char. 40: 1 --> 0
Char. 77: 1 --> 2
Char. 112: 1 --> 0
Char. 115: 0 --> 1

Phenacodonta :
All trees:
Char. 83: 1 --> 2
Char. 98: 0 --> 1
Char. 110: 1 --> 0
Some trees:
Char. 37: 0 --> 1
Char. 38: 1 --> 0
Char. 78: 1 --> 2
Char. 91: 1 --> 0
Char. 97: 0 --> 1
Char. 100: 0 --> 1
Char. 106: 2 --> 1
Char. 119: 0 --> 2
Char. 131: 0 --> 1
Char. 163: 1 --> 0

Perisssodactyla :
All trees:
Char. 83: 1 --> 2
Some trees:
Char. 91: 1 --> 0
Char. 142: 0 --> 1
Char. 155: 0 --> 1

Radinskya :
All trees:
Char. 104: 0 --> 3
Char. 112: 1 --> 2
Char. 124: 1 --> 0

Hyopsodus :
All trees:
Char. 5: 0 --> 1
Char. 7: 0 --> 1
Char. 10: 01 --> 2
Char. 12: 0 --> 1
Char. 28: 0 --> 1
Char. 69: 0 --> 1
Char. 158: 0 --> 1
Char. 162: 0 --> 1
Some trees:
Char. 38: 1 --> 0
Char. 64: 0 --> 1
Char. 65: 0 --> 1
Char. 119: 0 --> 2
Char. 129: 0 --> 1
Char. 132: 1 --> 0
Char. 136: 0 --> 1
Char. 146: 0 --> 1
Char. 150: 0 --> 1
Char. 151: 0 --> 1
Char. 163: 1 --> 0

Todralestes :
All trees:
Char. 9: 0 --> 1
Char. 52: 01 --> 2
Char. 96: 0 --> 1
Some trees:
Char. 10: 0 --> 1
Char. 18: 0 --> 1
Char. 45: 0 --> 1
Char. 104: 0 --> 1

Ptolemaia :
All trees:
Char. 110: 1 --> 2
Char. 111: 1 --> 2
Char. 136: 0 --> 1
Some trees:
Char. 45: 0 --> 1
Char. 58: 1 --> 0
Char. 61: 1 --> 0

Potamogale :
All trees:
Char. 5: 0 --> 1
Char. 7: 0 --> 1
Char. 38: 1 --> 0
Char. 45: 01 --> 2
Char. 69: 0 --> 1
Char. 70: 1 --> 0
Char. 87: 1 --> 0
Char. 93: 1 --> 0
Char. 99: 0 --> 1
Char. 103: 0 --> 1
Char. 121: 0 --> 2
Char. 124: 0 --> 1
Char. 133: 1 --> 3
Char. 134: 0 --> 1
Char. 135: 1 --> 2
Char. 136: 0 --> 1
Char. 138: 0 --> 2
Char. 154: 0 --> 1
Char. 159: 0 --> 1
Some trees:
Char. 58: 1 --> 0
Char. 61: 1 --> 0
Char. 101: 1 --> 0
Char. 146: 0 --> 1
Char. 167: 0 --> 1

Orycteropus :
All trees:
Char. 7: 0 --> 3
Char. 9: 0 --> 2
Char. 58: 01 --> 2
Char. 69: 0 --> 2
Char. 73: 0 --> 1
Char. 76: 1 --> 2
Char. 81: 1 --> 0
Char. 116: 0 --> 1
Char. 118: 0 --> 1
Char. 120: 0 --> 1
Char. 125: 0 --> 1
Char. 133: 1 --> 0
Char. 134: 0 --> 1
Char. 137: 1 --> 2
Char. 158: 0 --> 1
Some trees:
Char. 52: 1 --> 0
Char. 59: 1 --> 0
Char. 146: 0 --> 1

Macroscelidea :
All trees:
Char. 17: 0 --> 1
Char. 50: 0 --> 1
Char. 79: 0 --> 1
Some trees:
Char. 7: 0 --> 1
Char. 25: 012 --> 0
Char. 26: 0 --> 1
Char. 29: 01 --> 0
Char. 31: 02 --> 1
Char. 33: 01 --> 0
Char. 35: 1 --> 0
Char. 40: 01 --> 1
Char. 65: 0 --> 2
Char. 70: 1 --> 0
Char. 77: 12 --> 1
Char. 105: 1 --> 0
Char. 115: 0 --> 1
Char. 123: 0 --> 1

Char. 134: 0 --> 1
Char. 146: 0 --> 1
Char. 148: 0 --> 1
Char. 150: 0 --> 1
Char. 168: 0 --> 1

Hyracoidea :
All trees:
Char. 8: 1 --> 0
Char. 39: 123 --> 0
Char. 51: 0 --> 1
Char. 53: 2 --> 1
Char. 74: 0 --> 1
Char. 75: 0 --> 2
Char. 93: 2 --> 1
Char. 98: 0 --> 2
Char. 100: 01 --> 2
Char. 102: 1 --> 0
Char. 112: 1 --> 2
Char. 148: 0 --> 2
Char. 167: 0 --> 2
Some trees:
Char. 11: 1 --> 0
Char. 15: 0 --> 1
Char. 65: 0 --> 2
Char. 132: 1 --> 0
Char. 136: 1 --> 0
Char. 159: 0 --> 1

Anthracobunia :
All trees:
Char. 30: 0 --> 1
Char. 79: 0 --> 1
Char. 104: 0 --> 3
Char. 134: 02 --> 1
Some trees:
Char. 0: 1 --> 0
Char. 38: 1 --> 0
Char. 47: 0 --> 12
Char. 54: 1 --> 0
Char. 61: 2 --> 1
Char. 69: 1 --> 0
Char. 82: 1 --> 2
Char. 111: 0 --> 3

Desmostylia :
All trees:
Char. 47: 0 --> 2
Char. 53: 2 --> 1
Char. 58: 2 --> 1
Char. 122: 1 --> 2
Char. 123: 0 --> 1
Some trees:
Char. 11: 0 --> 1
Char. 15: 1 --> 0
Char. 21: 1 --> 0
Char. 69: 1 --> 0
Char. 82: 1 --> 0
Char. 83: 1 --> 0

Sirenia :
All trees:
Char. 8: 1 --> 0
Char. 12: 0 --> 2
Char. 13: 0 --> 1
Char. 44: 1 --> 0
Char. 70: 1 --> 0
Char. 76: 1 --> 0
Char. 81: 1 --> 0
Char. 121: 0 --> 2
Char. 127: 1 --> 2
Char. 134: 02 --> 3
Char. 138: 1 --> 2
Char. 139: 0 --> 1
Char. 159: 0 --> 1
Char. 170: 1 --> 0
Some trees:
Char. 50: 0 --> 2
Char. 74: 0 --> 2
Char. 77: 12 --> 0
Char. 89: 2 --> 1
Char. 137: 0 --> 1
Char. 167: 0 --> 1

Phenacolophus :
All trees:
Char. 1: 1 --> 0
Char. 11: 0 --> 1
Char. 15: 1 --> 0
Char. 18: 1 --> 0
Char. 29: 1 --> 0
Char. 35: 1 --> 0
Char. 47: 0 --> 12
Char. 110: 1 --> 0
Some trees:
Char. 74: 0 --> 2
Char. 77: 12 --> 0
Char. 134: 2 --> 0

Minchenella :
All trees:
Char. 16: 0 --> 1
Char. 45: 0 --> 2
Char. 46: 1 --> 2
Char. 106: 1 --> 2
Char. 114: 1 --> 0

Embrithopoda :
All trees:
Char. 3: 0 --> 1
Char. 26: 0 --> 1
Char. 31: 2 --> 1
Char. 34: 1 --> 2
Char. 62: 0 --> 1
Char. 93: 12 --> 0
Char. 100: 2 --> 3
Char. 102: 1 --> 0
Char. 104: 0 --> 2
Some trees:
Char. 50: 0 --> 2
Char. 77: 012 --> 2

Eritherium :
All trees:
Char. 42: 01 --> 2
Char. 43: 01 --> 2
Char. 46: 1 --> 2
Char. 48: 2 --> 1
Char. 82: 1 --> 0
Char. 83: 1 --> 0
Char. 98: 0 --> 1
Some trees:
Char. 31: 0 --> 1
Char. 78: 1 --> 0

Phosphatherium :
All trees:
Char. 58: 2 --> 1
Char. 98: 0 --> 1
Char. 119: 0 --> 2
Char. 170: 2 --> 1

Numidotherium :
All trees:
Char. 7: 2 --> 3
Char. 9: 0 --> 2
Char. 21: 0 --> 1
Char. 51: 0 --> 1
Char. 52: 01 --> 3
Char. 54: 0 --> 1
Char. 68: 0 --> 2
Char. 71: 0 --> 1
Char. 74: 0 --> 1
Char. 75: 0 --> 1
Char. 99: 0 --> 1
Char. 112: 1 --> 2
Char. 114: 1 --> 2
Char. 117: 0 --> 1
Char. 120: 0 --> 1
Char. 123: 0 --> 1
Char. 126: 0 --> 1
Char. 135: 1 --> 2
Char. 138: 0 --> 1
Char. 148: 0 --> 1
Char. 150: 0 --> 1
Char. 162: 0 --> 1
Char. 171: 0 --> 1
Some trees:
Char. 37: 1 --> 0
Char. 43: 1 --> 0
Char. 97: 1 --> 0
Char. 156: 0 --> 1
Char. 157: 1 --> 2

Node 26 :
All trees:
Char. 110: 0 --> 1
Char. 111: 0 --> 1
Some trees:
Char. 24: 0 --> 1
Char. 40: 0 --> 1
Char. 87: 0 --> 1
Char. 90: 0 --> 1

Node 27 :
All trees:
No synapomorphies

Node 28 :
All trees:
Char. 50: 0 --> 2
Char. 53: 2 --> 3
Char. 92: 0 --> 1
Char. 93: 2 --> 1
Char. 135: 0 --> 1
Char. 147: 0 --> 1
Some trees:
Char. 64: 0 --> 1
Char. 90: 1 --> 0
Char. 102: 1 --> 0
Char. 170: 0 --> 1

Node 29 :
All trees:
Char. 10: 01 --> 3
Char. 71: 0 --> 1
Char. 77: 01 --> 2
Some trees:
Char. 65: 0 --> 1
Char. 104: 0 --> 12

Node 30 :
All trees:
Char. 48: 0 --> 1
Char. 78: 0 --> 12
Char. 80: 0 --> 1
Char. 82: 01 --> 2
Char. 107: 0 --> 1
Char. 121: 0 --> 2
Char. 124: 0 --> 1
Some trees:
Char. 41: 0 --> 1
Char. 58: 1 --> 2
Char. 77: 0 --> 12
Char. 83: 0 --> 1
Char. 88: 0 --> 1
Char. 92: 0 --> 1
Char. 106: 0 --> 2
Char. 112: 0 --> 1

Node 31 :
All trees:
Char. 39: 0 --> 1
Char. 96: 0 --> 2
Some trees:
Char. 21: 0 --> 1
Char. 31: 1 --> 2
Char. 35: 0 --> 1
Char. 59: 0 --> 1
Char. 133: 1 --> 2
Char. 154: 0 --> 1
Char. 170: 01 --> 2

Node 32 :
All trees:
Char. 108: 0 --> 1
Char. 170: 2 --> 0
Some trees:
Char. 88: 1 --> 2
Char. 104: 1 --> 0
Char. 116: 1 --> 0
Char. 132: 1 --> 0
Char. 136: 1 --> 0
Char. 159: 0 --> 1

Node 33 :
All trees:
Char. 26: 0 --> 2
Char. 48: 0 --> 1
Char. 49: 0 --> 2
Char. 115: 0 --> 2

Node 34 :
Paenungulata
All trees:
Char. 27: 0 --> 1
Char. 60: 0 --> 1
Char. 114: 0 --> 1

Char. 121: 2 --> 0
Char. 160: 0 --> 1
Some trees:
Char. 1: 0 --> 1
Char. 18: 0 --> 1
Char. 21: 1 --> 0
Char. 25: 1 --> 2
Char. 28: 0 --> 1
Char. 36: 1 --> 0
Char. 69: 0 --> 1
Char. 116: 01 --> 1
Char. 129: 0 --> 1
Char. 131: 0 --> 1
Char. 134: 0 --> 2
Char. 167: 1 --> 0

Node 35 :
All trees:
Char. 51: 0 --> 1
Char. 106: 2 --> 1
Some trees:
Char. 23: 0 --> 1
Char. 54: 0 --> 1
Char. 61: 1 --> 2
Char. 104: 12 --> 0
Char. 138: 0 --> 1

Node 36 :
All trees:
Char. 24: 2 --> 3
Char. 32: 0 --> 1
Char. 43: 12 --> 0
Char. 97: 0 --> 1
Char. 98: 0 --> 2
Char. 100: 01 --> 2

Node 37 :
All trees:
Char. 25: 2 --> 1
Char. 28: 1 --> 0
Char. 40: 0 --> 1
Char. 91: 1 --> 0
Char. 101: 1 --> 0
Char. 109: 1 --> 0
Some trees:
Char. 38: 1 --> 0
Char. 111: 0 --> 3

Node 38 :
All trees:
Char. 7: 01 --> 2
Char. 12: 0 --> 1
Char. 16: 0 --> 1
Char. 20: 0 --> 1
Char. 40: 0 --> 2
Char. 89: 2 --> 1
Some trees:
Char. 0: 0 --> 1
Char. 3: 0 --> 1
Char. 15: 01 --> 0
Char. 82: 2 --> 1
Char. 102: 1 --> 2
Char. 135: 0 --> 1
Char. 144: 0 --> 1

Node 39 :
All trees:
Char. 3: 1 --> 2
Char. 6: 01 --> 2
Char. 10: 012 --> 3
Char. 24: 2 --> 3
Char. 32: 0 --> 1
Char. 34: 1 --> 2
Char. 35: 1 --> 0
Char. 46: 1 --> 0
Char. 50: 0 --> 2
Char. 85: 0 --> 1
Char. 88: 1 --> 3
Char. 92: 1 --> 0
Char. 93: 2 --> 3
Char. 106: 2 --> 3
Char. 111: 01 --> 3
Char. 134: 2 --> 3
Char. 139: 0 --> 1

3.2 Implied weighting (standard) analysis

Cladogram 10

1 trees retained.

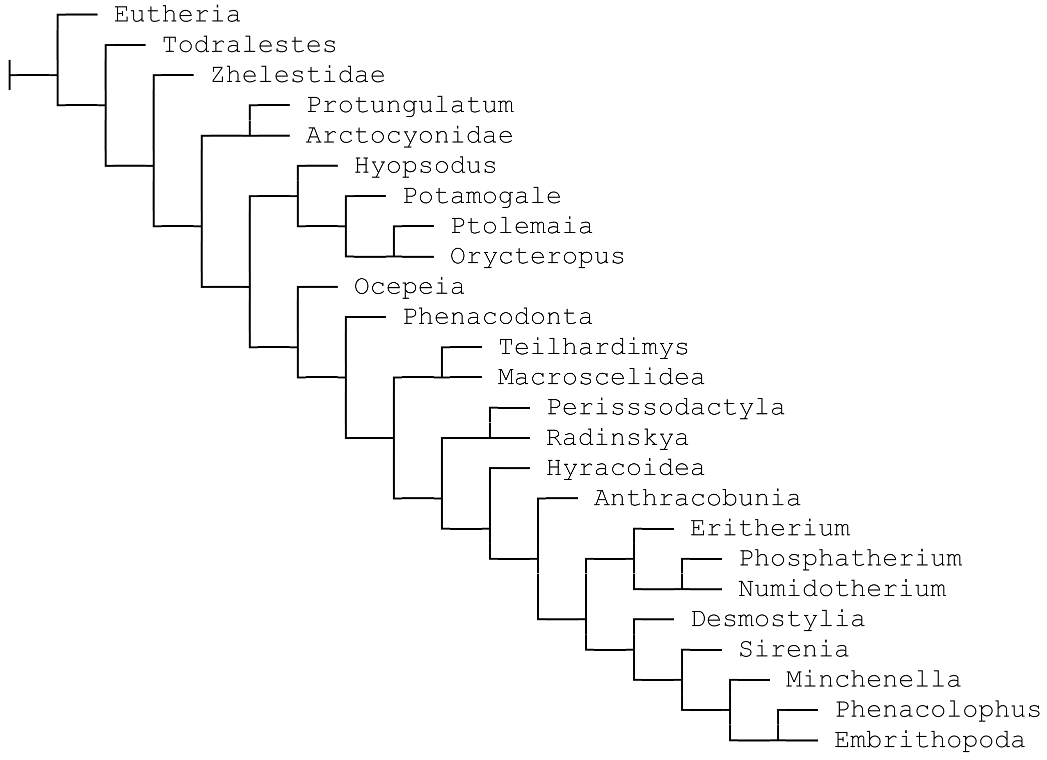

Tree length: 671. Retention index: 53.8; Consistency Index: 36.5

3.3 Unweighted and partitioned analysis: Dental and mandibular characters

Cladogram 11

1 trees retained.

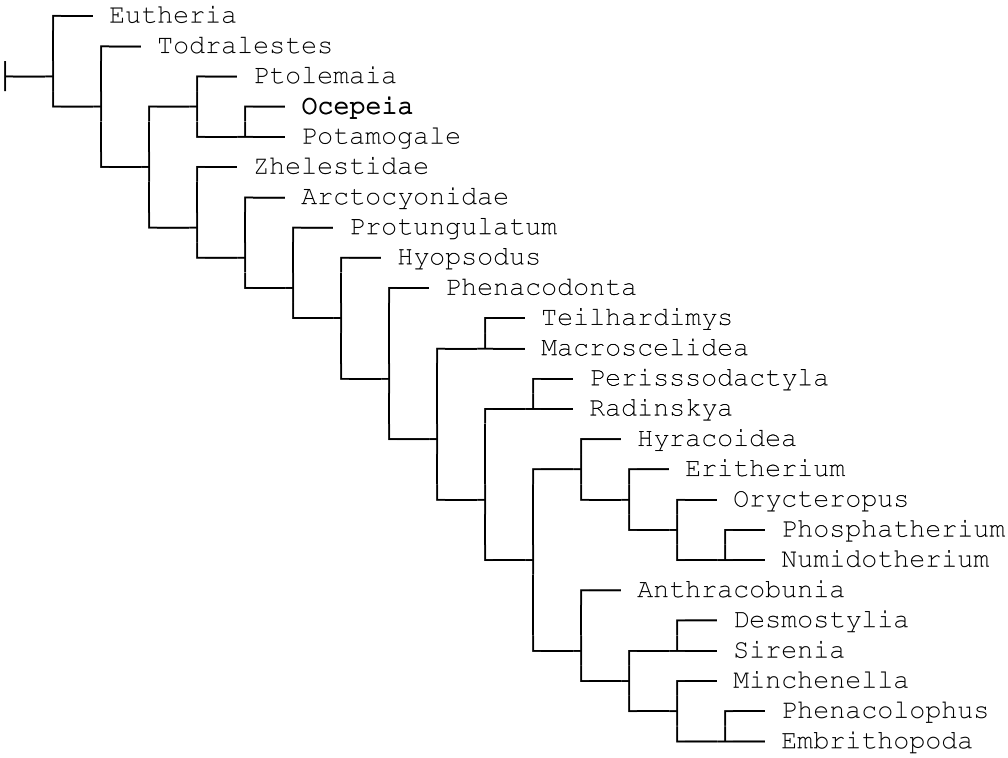

Tree length: 474. Retention index: 58.1; Consistency Index: 36.1

3.4 Unweighted and partitioned analysis: Skull characters

Cladogram 12

```
640 trees retained. Strict consensus of 640 trees (Tree length = 165)
   ┌── Eutheria
   │  ┌── Zhelestidae
   │  ├── Protungulatum
   │  ├── Arctocyonidae
   │  ├── Ocepeia
   │  ├── Teilhardimys
   │  ├── Phenacodonta
├──┤  ├── Perisssodactyla
   │  ├── Radinskya
   │  ├── Hyopsodus
   │  ├── Todralestes
   │  ├── Ptolemaia
   │  ├── Potamogale
   └──┼── Orycteropus
      ├── Macroscelidea
      ├── Hyracoidea
      ├── Anthracobunia
      ├── Desmostylia
      ├── Sirenia
      ├── Phenacolophus
      ├── Minchenella
      ├── Embrithopoda
      ├── Eritherium
      ├── Phosphatherium
      └── Numidotherium
```

4. Constrained analysis on matrix with ordered characters

Characters related to the selenodont pattern of upper molars (ectoloph dilambdodont, characters 98, 99, 101) are overweighted (weight x 8) in order to test relationships of *Ocepeia* with Paenungulata, and resulting topology and characters transformations.

Character weights (Numbering N-1)

	+0	+1	+2	+3	+4	+5	+6	+7	+8	+9
0	1	1	1	1	1	1	1	1	1	1
10	1	1	1	1	1	1	1	1	1	1
20	1	1	1	1	1	1	1	1	1	1
30	1	1	1	1	1	1	1	1	1	1
40	1	1	1	1	1	1	1	1	1	1
50	1	1	1	1	1	1	1	1	1	1
60	1	1	1	1	1	1	1	1	1	1
70	1	1	1	1	1	1	1	1	1	1
80	1	1	1	1	1	1	1	1	1	1
90	1	1	1	1	1	1	1	8	8	1
100	8	1	1	1	1	1	1	1	1	1
110	1	1	1	1	1	1	1	1	1	1
120	1	1	1	1	1	1	1	1	1	1
130	1	1	1	1	1	1	1	1	1	1
140	1	1	1	1	1	1	1	1	1	1
150	1	1	1	1	1	1	1	1	1	1
160	1	1	1	1	1	1	1	1	1	1
170	1	1	1	1	1					

Cladogram 13

2 trees retained. Strict consensus of 2 trees (0 taxa excluded)

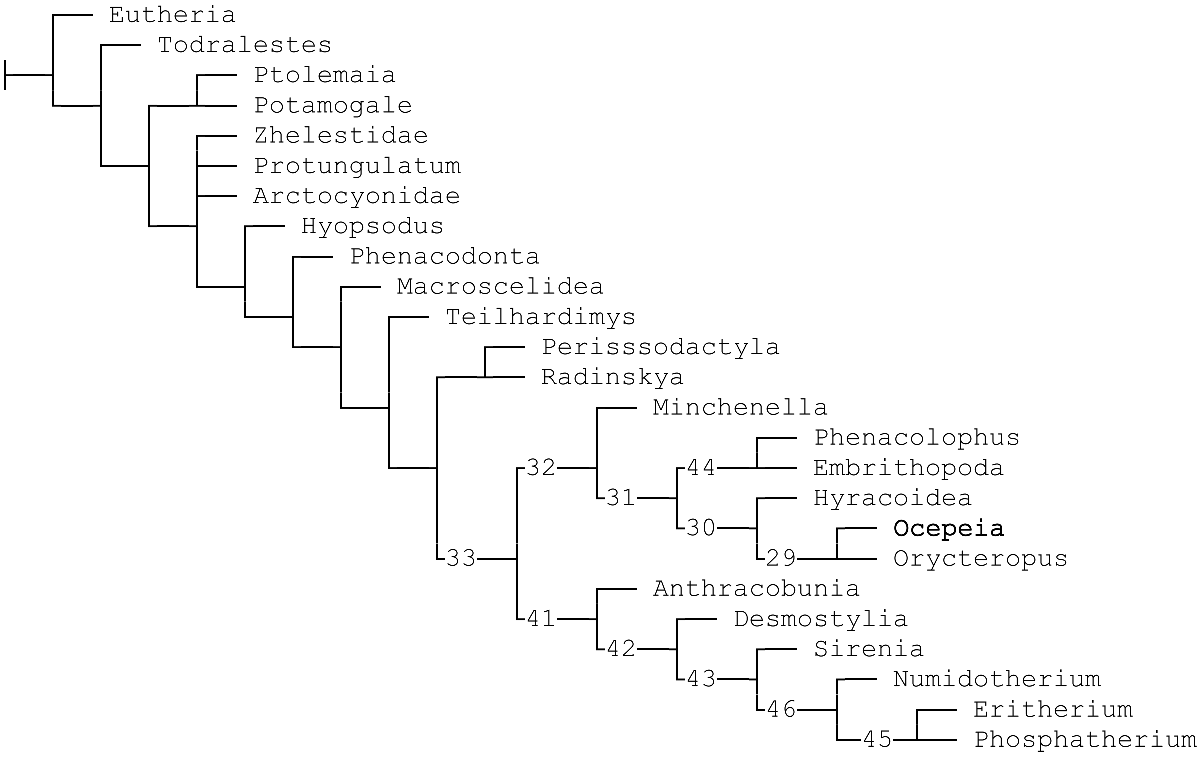

Tree length: 762. Retention index: 53.2; Consistency Index: 37,5

Bremer supports (from 100 trees, cut 0)

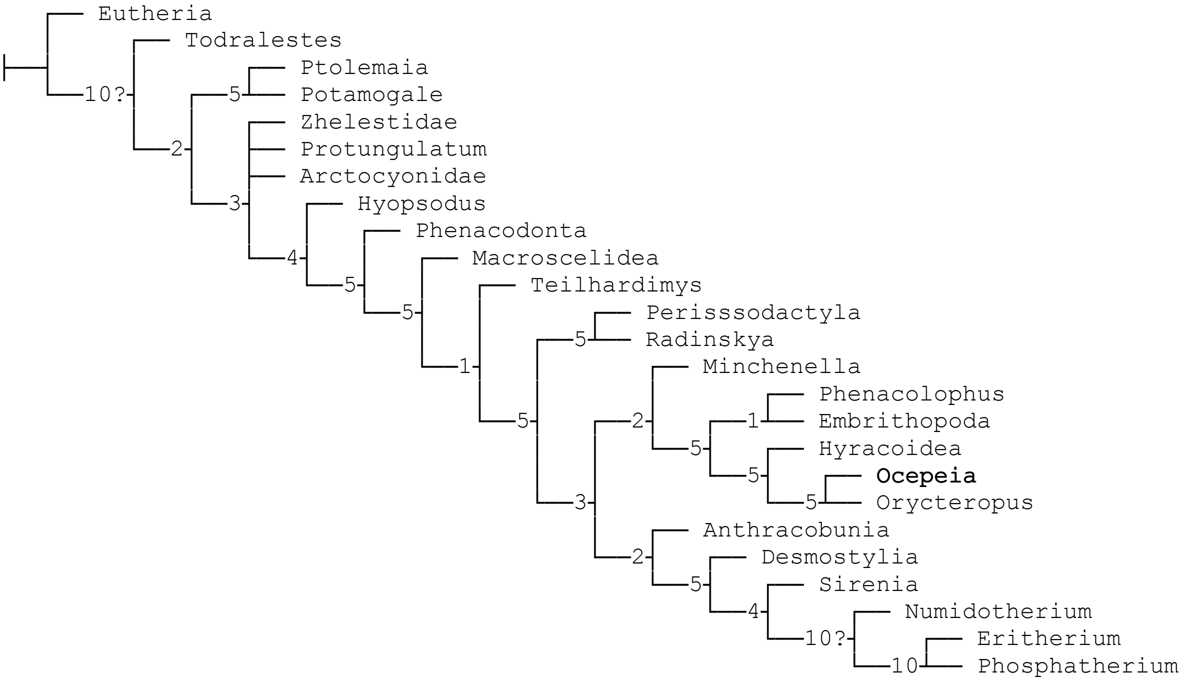

Diagnose of nodes of cladogram 13 : Synapomorphies

Important note: For correspondence with character description (Part II) and character summary list (Part III), number n of characters ***should be read as n+1*** because TNT starts numbering characters from 0.

Eutheria :
All trees:
No autapomorphies:

Zhelestidae :
All trees:
Char. 10: 1 --> 0
Char. 47: 0 --> 1
Char. 50: 0 --> 1
Char. 68: 0 --> 1
Some trees:
Char. 11: 1 --> 0
Char. 21: 0 --> 1
Char. 76: 1 --> 0
Char. 82: 0 --> 1
Char. 83: 0 --> 1
Char. 163: 1 --> 0

Protungulatum :
All trees:
Char. 27: 0 --> 1
Char. 61: 1 --> 2
Char. 143: 0 --> 2
Some trees:
Char. 35: 0 --> 1
Char. 92: 0 --> 1

Arctocyonidae :
All trees:
Char. 30: 0 --> 1
Some trees:
Char. 131: 0 --> 1
Char. 136: 1 --> 0
Char. 155: 0 --> 1

Ocepeia :
All trees:
Char. 7: 1 --> 0
Char. 47: 0 --> 1
Char. 48: 1 --> 0
Char. 50: 0 --> 2
Char. 52: 0 --> 2
Char. 58: 2 --> 1
Char. 69: 1 --> 0
Char. 116: 1 --> 0
Char. 117: 0 --> 2
Char. 121: 0 --> 1
Char. 140: 0 --> 1
Char. 147: 0 --> 1
Char. 161: 0 --> 1
Char. 162: 0 --> 1
Char. 167: 2 --> 0
Char. 168: 0 --> 1

Teilhardimys :
All trees:
Char. 16: 0 --> 1
Char. 20: 0 --> 1
Char. 21: 1 --> 0
Char. 22: 1 --> 0
Char. 25: 1 --> 2
Char. 28: 0 --> 1
Char. 30: 0 --> 1
Char. 53: 2 --> 3
Char. 74: 0 --> 1
Char. 75: 0 --> 2
Char. 112: 1 --> 0
Some trees:
Char. 31: 2 --> 0

Phenacodonta :
All trees:
Char. 10: 1 --> 0
Char. 37: 0 --> 1
Char. 78: 1 --> 2
Char. 83: 1 --> 2
Char. 97: 0 --> 1
Char. 98: 0 --> 1
Char. 100: 0 --> 1
Char. 110: 1 --> 0
Char. 131: 0 --> 1

Perisssodactyla :
All trees:
Char. 83: 1 --> 2
Char. 91: 1 --> 0
Char. 104: 1 --> 0
Char. 155: 0 --> 1

Radinskya :
All trees:
Char. 104: 1 --> 3
Char. 112: 1 --> 2
Char. 124: 1 --> 0

Hyopsodus :
All trees:
Char. 5: 0 --> 1
Char. 7: 0 --> 1
Char. 10: 1 --> 2
Char. 12: 0 --> 1
Char. 28: 0 --> 1
Char. 65: 0 --> 1
Char. 69: 0 --> 1
Char. 132: 1 --> 0
Char. 146: 0 --> 1
Char. 150: 0 --> 1
Char. 151: 0 --> 1
Char. 158: 0 --> 1
Char. 162: 0 --> 1

Todralestes :
All trees:
Char. 9: 0 --> 1
Char. 18: 0 --> 1
Char. 52: 1 --> 2
Char. 96: 0 --> 1

Ptolemaia :
All trees:
Char. 18: 0 --> 1
Char. 26: 0 --> 2
Char. 38: 1 --> 2
Char. 48: 0 --> 1
Char. 49: 0 --> 2
Char. 80: 0 --> 1
Char. 82: 0 --> 1
Char. 83: 0 --> 1
Char. 91: 0 --> 1
Char. 93: 2 --> 3
Char. 101: 0 --> 1
Char. 110: 1 --> 2
Char. 111: 1 --> 2
Char. 115: 0 --> 2

Potamogale :
All trees:
Char. 7: 0 --> 1
Char. 16: 0 --> 1
Char. 38: 1 --> 0
Char. 45: 1 --> 2
Char. 46: 1 --> 2
Char. 50: 0 --> 2
Char. 53: 2 --> 3
Char. 64: 0 --> 1
Char. 65: 1 --> 2
Char. 69: 0 --> 1
Char. 92: 0 --> 1
Char. 93: 2 --> 0
Char. 99: 0 --> 1
Char. 103: 0 --> 1
Char. 133: 1 --> 3
Char. 134: 1 --> 0
Char. 135: 0 --> 2
Char. 138: 0 --> 2
Char. 146: 0 --> 1
Char. 147: 0 --> 1
Char. 159: 0 --> 1

Orycteropus :
All trees:
Char. 6: 1 --> 2
Char. 7: 1 --> 3
Char. 9: 01 --> 2
Char. 26: 0 --> 2
Char. 49: 0 --> 2
Char. 51: 1 --> 0
Char. 59: 1 --> 0
Char. 69: 1 --> 2
Char. 76: 1 --> 2
Char. 81: 1 --> 0
Char. 115: 0 --> 2
Char. 118: 0 --> 1
Char. 120: 0 --> 1
Char. 122: 1 --> 4
Char. 123: 0 --> 1
Char. 133: 2 --> 0
Char. 134: 1 --> 0
Char. 137: 1 --> 2
Char. 146: 0 --> 1
Char. 150: 0 --> 1
Char. 158: 0 --> 1
Char. 160: 1 --> 0
Char. 166: 0 --> 2
Char. 170: 12 --> 0
Char. 171: 0 --> 1

Macroscelidea :
All trees:
Char. 7: 0 --> 1
Char. 17: 0 --> 1
Char. 31: 02 --> 1
Char. 50: 0 --> 1
Char. 65: 0 --> 2
Char. 70: 1 --> 0
Char. 79: 0 --> 1
Char. 134: 1 --> 0
Char. 146: 0 --> 1
Char. 148: 0 --> 1
Char. 150: 0 --> 1
Char. 168: 0 --> 1
Some trees:
Char. 35: 1 --> 0

Hyracoidea :
All trees:
Char. 8: 1 --> 0
Char. 25: 1 --> 2
Char. 28: 0 --> 1
Char. 40: 1 --> 0
Char. 53: 2 --> 1
Char. 104: 1 --> 2
Char. 112: 1 --> 2
Char. 125: 1 --> 0
Char. 132: 1 --> 0
Char. 134: 1 --> 2
Char. 145: 0 --> 1
Char. 148: 0 --> 2
Char. 151: 1 --> 0
Char. 155: 0 --> 1
Char. 159: 0 --> 1

Anthracobunia :
All trees:
Char. 30: 0 --> 1
Char. 43: 1 --> 2
Char. 79: 0 --> 1
Char. 104: 1 --> 3

Desmostylia :
All trees:
Char. 47: 01 --> 2
Char. 53: 2 --> 1
Char. 58: 2 --> 1
Char. 78: 1 --> 0
Char. 82: 1 --> 0
Char. 83: 1 --> 0
Char. 122: 1 --> 2
Char. 157: 1 --> 2

Sirenia :
All trees:
Char. 8: 1 --> 0
Char. 12: 1 --> 2
Char. 43: 1 --> 2
Char. 65: 0 --> 2
Char. 70: 1 --> 0
Char. 74: 0 --> 2
Char. 76: 1 --> 0
Char. 77: 12 --> 0
Char. 81: 1 --> 0
Char. 127: 0 --> 2
Char. 138: 1 --> 2
Char. 159: 0 --> 1
Char. 166: 0 --> 2
Char. 170: 12 --> 0
Char. 174: 2 --> 0

Phenacolophus :
All trees:
Char. 11: 0 --> 1
Char. 15: 1 --> 0
Char. 18: 1 --> 0
Char. 29: 1 --> 0
Char. 35: 1 --> 0
Char. 47: 0 --> 12
Char. 74: 0 --> 2
Char. 77: 2 --> 0
Char. 90: 0 --> 1
Char. 104: 1 --> 0
Char. 110: 1 --> 0

Minchenella :
All trees:
Char. 16: 0 --> 1
Char. 17: 0 --> 1
Char. 43: 01 --> 2
Char. 45: 0 --> 2
Char. 104: 1 --> 0

Embrithopoda :
All trees:
Char. 3: 0 --> 1
Char. 17: 0 --> 1
Char. 26: 0 --> 1
Char. 31: 2 --> 1
Char. 34: 1 --> 2
Char. 50: 0 --> 2
Char. 93: 1 --> 0
Char. 100: 2 --> 3
Char. 104: 1 --> 2
Char. 134: 1 --> 2

Eritherium :
All trees:
Char. 31: 0 --> 1
Char. 42: 1 --> 2
Char. 43: 1 --> 2
Char. 46: 01 --> 2
Char. 48: 2 --> 1
Char. 50: 2 --> 0
Char. 78: 1 --> 0
Char. 82: 1 --> 0
Char. 83: 1 --> 0
Char. 111: 3 --> 1
Char. 134: 3 --> 2
Char. 139: 1 --> 0

Phosphatherium :
All trees:
No autapomorphies:

Numidotherium :
All trees:
Char. 7: 2 --> 3
Char. 9: 0 --> 2
Char. 43: 1 --> 0
Char. 52: 0 --> 3
Char. 68: 0 --> 2
Char. 71: 0 --> 1
Char. 74: 0 --> 1
Char. 75: 0 --> 1
Char. 112: 1 --> 2
Char. 114: 1 --> 2
Char. 117: 0 --> 1
Char. 120: 0 --> 1
Char. 135: 1 --> 2
Char. 148: 0 --> 1
Char. 150: 0 --> 1
Char. 157: 1 --> 2
Char. 162: 0 --> 1
Char. 171: 0 --> 1

Node 26 :
All trees:
Char. 40: 0 --> 1
Char. 52: 1 --> 0
Char. 58: 0 --> 1
Char. 61: 0 --> 1
Some trees:
Char. 44: 0 --> 1

Node 27 :
All trees:
Char. 24: 0 --> 1
Char. 110: 0 --> 1
Char. 111: 0 --> 1

Node 28 :
All trees:
No synapomorphies

Node 29 :
All trees:
Char. 6: 0 --> 1
Char. 10: 0 --> 3
Char. 12: 0 --> 3
Char. 15: 1 --> 0
Char. 16: 0 --> 1
Char. 44: 1 --> 0
Char. 48: 2 --> 1
Char. 60: 1 --> 0
Char. 71: 0 --> 1
Char. 73: 0 --> 1
Char. 124: 1 --> 0
Char. 129: 1 --> 0
Char. 154: 1 --> 0
Char. 157: 1 --> 0

Node 30 :
All trees:
Char. 21: 1 --> 0
Char. 23: 1 --> 0
Char. 37: 0 --> 1
Char. 39: 3 --> 0
Char. 41: 3 --> 2
Char. 136: 1 --> 0
Char. 137: 0 --> 1

Node 31 :
All trees:
Char. 93: 2 --> 1
Char. 97: 0 --> 1
Char. 98: 0 --> 2
Char. 100: 0 --> 2

Node 32 :
All trees:
Char. 7: 0 --> 1
Char. 40: 0 --> 1
Char. 90: 1 --> 0
Char. 91: 1 --> 0

Node 33 :
Paenungulata
All trees:
Char. 27: 0 --> 1
Char. 36: 1 --> 0
Char. 39: 2 --> 3
Char. 41: 2 --> 3
Char. 60: 0 --> 1
Char. 125: 0 --> 1
Char. 151: 0 --> 1
Char. 160: 0 --> 1

Node 34 :
All trees:
Char. 10: 1 --> 0
Char. 15: 0 --> 1
Char. 23: 0 --> 1
Char. 24: 1 --> 2
Char. 34: 0 --> 1
Char. 39: 1 --> 2
Char. 41: 1 --> 2
Char. 48: 1 --> 2
Char. 51: 0 --> 1
Char. 89: 1 --> 2
Char. 113: 0 --> 1

Node 35 :
All trees:
Char. 25: 0 --> 1
Char. 29: 0 --> 1
Char. 33: 0 --> 1
Char. 40: 1 --> 0
Char. 77: 1 --> 2
Char. 105: 0 --> 1

Node 36 :
All trees:
Char. 18: 0 --> 1
Char. 89: 0 --> 1
Char. 104: 0 --> 1
Char. 116: 0 --> 1
Char. 137: 1 --> 0
Char. 140: 1 --> 0

Node 37 :
All trees:
Char. 48: 0 --> 1
Char. 77: 0 --> 1
Char. 78: 0 --> 1
Char. 80: 0 --> 1
Char. 82: 01 --> 2
Char. 107: 0 --> 1
Char. 112: 0 --> 1
Char. 121: 0 --> 2
Char. 124: 0 --> 1
Some trees:
Char. 58: 1 --> 2
Char. 83: 0 --> 1
Char. 92: 0 --> 1

Node 38 :
All trees:
Char. 39: 0 --> 1
Char. 59: 0 --> 1
Char. 96: 0 --> 2
Some trees:
Char. 21: 0 --> 1
Char. 31: 1 --> 2
Char. 101: 0 --> 1

Node 39 :
All trees:
Char. 88: 1 --> 2
Char. 108: 0 --> 1
Char. 116: 1 --> 0
Char. 132: 1 --> 0
Char. 136: 1 --> 0
Char. 159: 0 --> 1
Char. 170: 12 --> 0

Node 40 :
All trees:
Char. 10: 1 --> 3
Char. 59: 0 --> 1
Char. 65: 0 --> 1
Char. 71: 0 --> 1
Char. 77: 0 --> 2
Char. 104: 01 --> 2
Char. 123: 0 --> 1

Node 41 :
All trees:
Char. 25: 1 --> 2
Char. 28: 0 --> 1
Char. 106: 2 --> 1
Char. 109: 0 --> 1

Node 42 :
All trees:
Char. 0: 0 --> 1
Char. 10: 0 --> 1
Char. 15: 1 --> 0
Char. 31: 2 --> 0

Char. 99: 0 --> 1
Char. 102: 1 --> 2

Node 43 :
All trees:
Char. 12: 0 --> 1
Char. 44: 1 --> 0
Char. 50: 0 --> 2
Char. 69: 0 --> 1
Char. 89: 2 --> 1
Char. 137: 0 --> 1
Char. 139: 0 --> 1

Node 44 :
All trees:
Char. 0: 0 --> 1
Char. 24: 2 --> 3
Char. 32: 0 --> 1

Node 45 :
All trees:
Char. 37: 0 --> 1
Char. 38: 1 --> 2
Char. 42: 0 --> 1
Char. 51: 1 --> 0
Char. 54: 1 --> 0
Char. 97: 0 --> 1
Char. 98: 0 --> 1
Char. 99: 1 --> 0
Char. 138: 1 --> 0

Node 46 :
All trees:
Char. 3: 0 --> 12
Char. 6: 0 --> 12
Char. 7: 01 --> 2
Char. 10: 1 --> 23
Char. 20: 0 --> 1
Char. 23: 1 --> 0
Char. 30: 0 --> 1
Char. 40: 0 --> 2
Char. 62: 0 --> 2
Char. 104: 01 --> 2
Char. 118: 0 --> 2
Char. 135: 0 --> 1
Char. 143: 0 --> 2
Char. 152: 0 --> 1
Char. 161: 0 --> 1
Char. 167: 1 --> 0
Char. 169: 1 --> 0

Part IV.
References

1. Gheerbrant E (2009) Paleocene emergence of elephant relatives and the rapid radiation of African ungulates. Proceedings of the National Academy of Sciences of the USA 106:10717-10721.

2. Gheerbrant E, Sudre J, Tassy P, Amaghzaz M, Bouya B, Iarochene M (2005a) Nouvelles données sur *Phosphatherium escuilliei* (Mammalia, Proboscidea) de l'Eocène inférieur du Maroc, apports à la phylogénie des Proboscidea et des ongulés lophodontes. Geodiversitas 27: 239-333.

3. Tabuce R, Marivaux L, Adaci M, Bensalah M, Hartenberger JL, Mahboubi M, Mebrouk F, Tafforeau P, Jaeger JJ (2007) Early Tertiary mammals from North Africa reinforce the molecular Afrotheria clade. Proceedings of the Royal Society B, 274: 1159 – 1166.

4. Sen S, Heintz E (1979) *Palaeoamasia kansui* Ozansoy 1966, embrithopode (Mammalia) de l’Eocène d’Anatolie. Annales de Paléontologie 65:73–91.

5. Simons EL, Holroyd PA, Bown TM (1991) Early tertiary elephant-shrews from Egypt and the origin of the Macroscelidea. Proceedings of the National Academy of Sciences USA 88: 9734-9737.

6. Domning DP, Ray C, Mckenna M C (1986) Two new Oligocene desmostylians and a discussion of tethytherian systematics. Smithsonian Contribution to Paleobiology 59: 1-56.

7. Wible JR, Rougier GW, Novacek MJ, Asher RJ (2009) The eutherian mammal *Maelestes gobiensis* from the Late Cretaceous of Mongolia and the phylogeny of Cretaceous Eutheria. Bulletin of the American Museum of Natural History 327: 1–123.

8. Seiffert E (2007) A new estimate of afrotherian phylogeny based on simultaneous analysis of genomic, morphological, and fossil evidence. BMC Evolutionary Biology 7: 224.

9. Court N (1992) Cochlea anatomy of *Numidotherium koholense* : auditory acuity in the oldest known proboscidean. Lethaia 25:211-215.

10. McKenna MC, Manning E (1977) Affinities and Paleobiogeographic significance of the Mongolian Paleogene genus *Phenacolophus*. Géobios Mém Spécial 1: 61-85.

11. Pickford M, Senut B, Morales J, Mein P, Sanchez I M, (2008) Mammalia from the Lutetian of Namibia. Memoir of the Geological Survey of Namibia. 20: 465–514.

12. Barrow E, Seiffert ER, Simons EL (2010) A primitive hyracoid (Mammalia, Paenungulata) from the early Priabonian (Late Eocene) of Egypt. Journal of Systematic Palaeontology 8: 213–244.

13. Cox PG (2006) Character evolution in the orbital region of the Afrotheria. Journal of Zoology 269: 514-526.

14. Asher RJ, Maree S, Bronner G, Bennett NC, Bloomer P, Czechowski P, Meyer M, Hofreiter M (2012) A phylogenetic estimate for golden moles (Mammalia, Afrotheria, Chrysochloridae). BMC Evolutionary Biology, 10:69.

15. Thewissen JGM, Domning DP (1992) The role of phenacodontids in the origin of the modern orders of ungulate mammals. Journal of Vertebrate Paleontology 12, 494-504.

16. Domning DP (1994) A phylogenetic analysis of the Sirenia. In: Whitmore FC, Berta A, Deméré TA (eds) Contribution in Marine Mammal Paleontology Honoring. Jr. Proc. San Diego Soc. Nat. Hist., 29, 177-189.

17. Russell DE (1964) Les Mammifères paléocènes d'Europe. Mémoires du Muséum National d'Histoire Naturelle, série C, Sciences de la Terre 13: 1-324.

18. Froehlich D W (2002) Quo vadis *eohippus*? The systematics and taxonomy of the early Eocene equids (Perissodactyla). Zoological Journal of the Linnean Society 134:141-256.

19. Savage DE, Domning DP, Thewissen JGM (1994) Fossil Sirenia of the West Atlantic and Carribean Region. V. The most primitive known sirenian, *Prorastomus sirenoides* Owen, 1855. Journal of Vertebrate Paleontology 14:427-449

20. Court N, Jaeger JJ (1991) Anatomy of the periotic bone in the Eocene Proboscidean *Numidotherium koholense*: An example of parallel evolution in the inner ear of tethytheres. Comptes Rendus de l'Académie des Sciences de Paris 312, II: 559-565.

21. Court N (1990) Periotic anatomy of *Arsinoitherium* (Mammlia, Embrithopoda) and its phylogenetic implications. Journal of Vertebrate Paleontology 10:170-182.

22. Geisler JH , Theodor JM , Uhen MD, Foss SE (2007) Phylogenetic relationships of cetaceans to terrestrial artiodactyls. In Prothero DR, Foss SE, editors. The evolution of artiodactyls. Baltimore MD: Johns Hopkins University Press. 19–31.

23. O'Leary MA (2010) Anatomical and phylogenetic study of the osteology of the petrosal of extant and extinct artiodactylans (Mammalia) and relatives. Bulletin of the American Museum ofNatural History 335:1–206.

24. O'Leary MA, Patel BA, Coleman MN (2012) Endocranial petrosal anatomy of *Bothriogenys* (Mammalia, Artiodactyla, Anthracotheriidae), and petrosal volume and density comparisons among aquatic and terrestrial artiodactyls and outgroup. Journal of Paleontology 86: 44–50.

25. Benoit J, Orliac M, Tabuce R (2013) The petrosal of *Chambius* (Macroscelidea, Afrotheria) from the Eocene of Djebel Chambi (Tunisia) and the evolution of the ear region in elephant-shrews. Journal of Systematic Palaeontology, DOI: 10.1080/14772019.2012.713400.

26. Benoit J, Adnet S, El Mabrouk E, Khayati H, Ben Haj Ali M, et al. (2013) Cranial Remain from Tunisia Provides New Clues for the Origin and Evolution of Sirenia (Mammalia, Afrotheria) in Africa. PLoS ONE 8(1): e54307. doi:10.1371/journal.pone.0054307

27. Court N (1994) The periotic of *Moeritherium* (Mammalia, Proboscidea) : homology or homoplasy in the ear region of Tethytheria McKenna, 1975 ? Zoological Journal of the Linnean Society 112:13-28.

28. Ekdale EG, Rowe T (2011) Morphology and variation within the bony labyrinth of zhelestids (Mammalia, Eutheria) and other therian mammals. Journal of Vertebrate Paleontology 31: 658–675.

29. Goloboff PA, Farris JS, Nixon KC (2008) TNT, a free program for phylogenetic analysis. *Cladistics* 24: 774-786.
